# Enzyme-independent role of EZH2 in regulating cell cycle progression via the SKP2-KIP/CIP pathway

**DOI:** 10.1038/s41598-024-64338-4

**Published:** 2024-06-11

**Authors:** Tania Colon, Ziyue Kou, Byeong Hyeok Choi, Franklin Tran, Edwin Zheng, Wei Dai

**Affiliations:** Division of Environmental Medicine, Department of Medicine Grossman School of Medicine, New York University, 341 East 25th Street, New York, NY 10010 USA

**Keywords:** Cancer, Cell biology, Molecular biology

## Abstract

While EZH2 enzymatic activity is well-known, emerging evidence suggests that EZH2 can exert functions in a methyltransferase-independent manner. In this study, we have uncovered a novel mechanism by which EZH2 positively regulates the expression of SKP2, a critical protein involved in cell cycle progression. We demonstrate that depletion of EZH2 significantly reduces SKP2 protein levels in several cell types, while treatment with EPZ-6438, an EZH2 enzymatic inhibitor, has no effect on SKP2 protein levels. Consistently, EZH2 depletion leads to cell cycle arrest, accompanied by elevated expression of CIP/KIP family proteins, including p21, p27, and p57, whereas EPZ-6438 treatment does not modulate their levels. We also provide evidence that EZH2 knockdown, but not enzymatic inhibition, suppresses SKP2 mRNA expression, underscoring the transcriptional regulation of SKP2 by EZH2 in a methyltransferase-independent manner. Supporting this, analysis of the Cancer Genome Atlas database reveals a close association between EZH2 and SKP2 expression in human malignancies. Moreover, EZH2 depletion but not enzymatic inhibition positively regulates the expression of major epithelial-mesenchymal transition (EMT) regulators, such as ZEB1 and SNAIL1, in transformed cells. Our findings shed light on a novel mechanism by which EZH2 exerts regulatory effects on cell proliferation and differentiation through its methyltransferase-independent function, specifically by modulating SKP2 expression.

## Introduction

Enhancer of Zeste Homolog 2 (EZH2) is a histone methyltransferase and a key component of the Polycomb Repressive Complex 2 (PRC2). It plays a crucial role in epigenetic regulation by catalyzing the trimethylation of histone H3 on lysine 27 (H3K27me3), which leads to gene silencing^1,2^. During the cell cycle, EZH2 is involved in the regulation of cell proliferation and differentiation^3,4^. It modulates the expression of various cell cycle-related genes, including cyclins, cyclin-dependent kinases (CDKs), and Rb protein. EZH2 promotes G1/S phase transition by silencing the expression of CDK inhibitors (CKIs), such as p21 and p27, leading to increased CDK activity and cell cycle progression^5,6^. EZH2 has been implicated in oncogenesis due to its role in promoting uncontrolled cell proliferation^7–9^. Aberrant EZH2 activity, which can be caused by overexpression or gain-of-function mutations, has been observed in various types of cancers, including prostate, breast, and lymphoma^10^. As a result, EZH2 has emerged as a potential therapeutic target for cancer treatment. Some studies have also suggested that EZH2 functions through enzyme-independent mechanisms^11^ further highlighting its importance and multiple roles in oncogenesis.

We have been studying the nuclear function of PTEN^12–15^. Where we showed that downregulation of either PTEN or CBX8 induced H3K27me3 epigenetic marker in mitotic cells, suggesting a regulatory relationship between nuclear PTEN and EZH2^13^. This prompted us further to study the EZH2 protein in the cell cycle progression, an area lacking sufficient exploration. S-phase kinase-associated protein 2 (SKP2) is an F-box protein and an essential component of the SCF (SKP1-CUL1-F-box) E3 ubiquitin ligase complex. It is involved in cell cycle regulation by targeting various proteins for ubiquitin-mediated degradation. The primary function of SKP2 is to control the G1/S phase transition by promoting the degradation of cyclin-dependent kinase inhibitors (CKIs) such as p21, p27, and p57^16,17^. SKP2 is overexpressed in various cancers, and its overexpression has been correlated with poor prognosis, tumor progression, and metastasis^18,19^. The oncogenic role of SKP2 is not only limited to the degradation of CKIs but also extends to other targets, such as E-cadherin, FOXO1, and RASSF1A, which are involved in processes like cell adhesion, apoptosis, and tumor suppression^20,21^. Moreover, SKP2 can modulate the expression of EMT and enhance metastasis^22^.

SKP2 is regulated at both transcriptional and post-translational levels. Several transcription factors, such as E2F1, c-Myc, and HIF-1α, regulate SKP2 gene expression^23–25^. Furthermore, post-translational modifications, including phosphorylation by kinases like CDK2 and AKT, can modulate SKP2 stability and function^26,27^. Anaphase promoting complex/cyclosome (APC/C) plays a major role in regulating the stability of SKP2^28^. In late mitosis and early G1 phase, APC/C associates with Cdh1, forming the active APC/C^Cdh1^ complex, which targets SKP2 for ubiquitination and subsequent degradation^28^. The regulation of SKP2 is considered a target for developing anticancer drugs to overcome resistance^22^.

Recent studies have revealed an enzyme-independent function of EZH2 in the regulation of various biological processes. For instance, it has been demonstrated that a deficiency in EZH2 globally impairs the translation process and reduces the efficiency of internal ribosome entry site (IRES)-dependent translation initiation^29^. Moreover, EZH2 has been identified as interacting with MYC, facilitating its stabilization independently of its methyltransferase activity^30^. Furthermore, EZH2 has been shown to act as a transcription activator, exhibiting roles that are both polycomb- and methylation-independent^31^. Although studies have demonstrated that EZH2 protein plays a role in regulating the cell cycle^32,33^, whether it involves non-enzymatic functions remains unexplored.

In our preliminary studies, we have demonstrated that knocking down EZH2 with siRNAs leads to increased CKI expression and a “G2/M-like” arrest. However, treatment with EPZ-6438 (Tazemetostat), an FDA-approved drug that acts as a potent selective EZH2 inhibitor, does not produce the same effects, suggesting an enzyme-independent role for EZH2 in regulating cell cycle progression. Further investigation revealed that EZH2 is involved in the positive regulation of SKP2, which controls CKI degradation. EZH2 physically interacts with SKP2, and its depletion via RNAi reduces SKP2 mRNA levels. In contrast, the enzymatic inhibitor EPZ-6438 does not affect SKP2 mRNA levels, again indicating a catalytically independent role for EZH2. These findings demonstrate the existence of an undescribed function of EZH2, highlighting the importance of further exploring its independent functions. Our work contributes to a better understanding of the role that EZH2 plays in cell cycle progression, underscoring the methyltransferase-independent function over its enzymatic activity. Therefore, targeting the methyltransferase-independent function of EZH2 holds promise as a new therapeutic strategy for cancer patients in the clinic.

## Results

### EZH2 knockdown induces expression of CIP/KIP family CKIs

**Figure 1:**
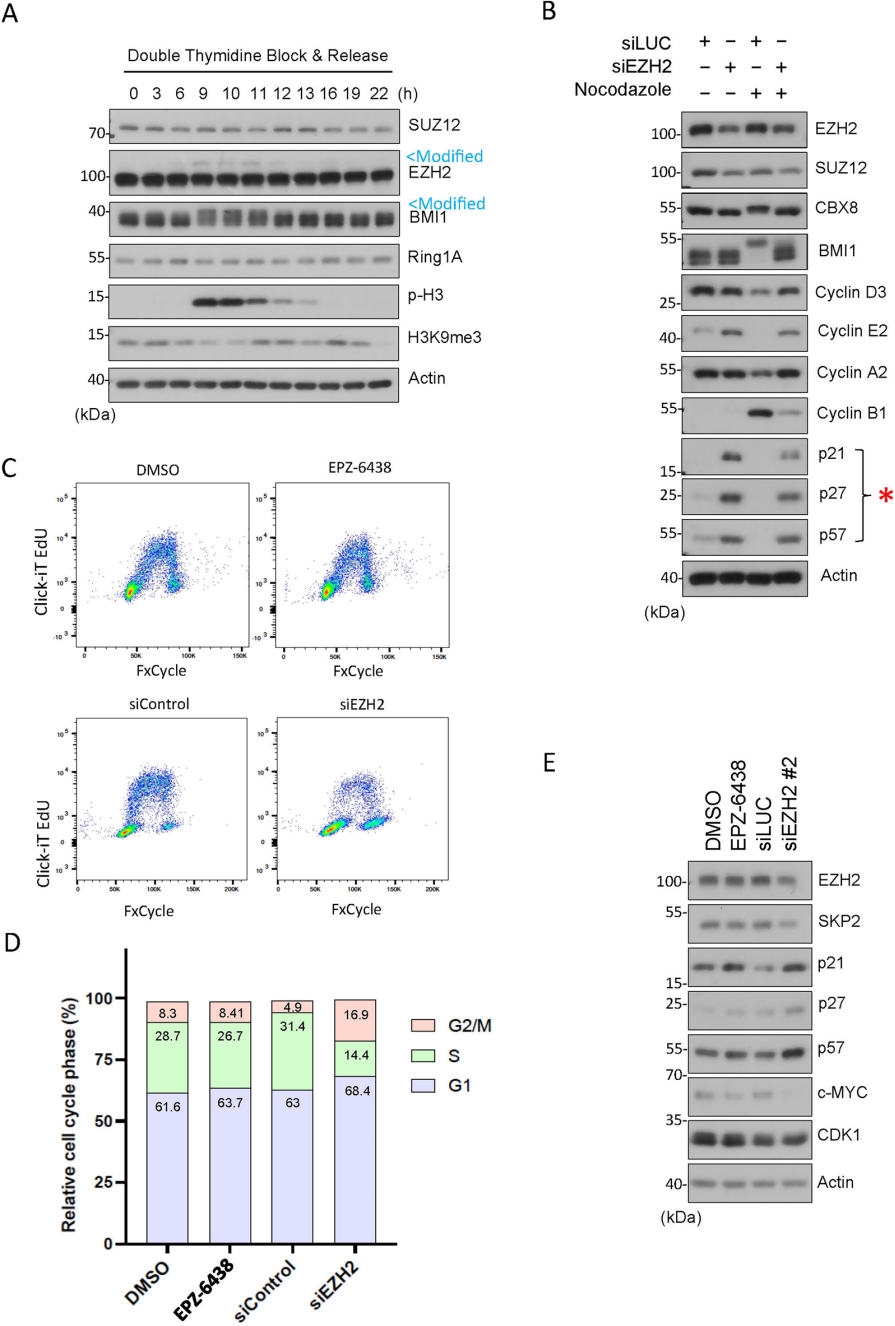
EZH2 knockdown induces expression of CIP/KIP family CKIs. (**A**) HeLa cells synchronized by double-thymidine block were released into the cell cycle. Cell lysates post release were blotted for PRC2 components (e.g., SUZ12, EZH2, BMI1, Ring1A), and p-H3, H3K9me3 and β-actin. Modified EZH2 and BMI1 with slow mobility are marked (in blue). (**B**) HeLa cells transfected with EZH2 or CNTL (siLuc) siRNAs for 48 h were collected, and cell lysates were blotted for various cell cycle proteins as indicated. (**C**) HeLa cells were treated with EPZ-6438 (an EZH2 inhibitor) or vehicle (DMSO) for 48 hor transfected with EZH2 (siEZH2) or CNTL (siControl) siRNAs for 48 h. Cells of both treatments were then stained with Click-iT EdU and FxCycle Violet (Invitrogen) for flow cytometry. (**D**) Cell cycle distributions (based on DNA content only) of various treatments as shown in (**B**) were summarized. (**E**) HeLa cells were treated with EPZ-6438 or vehicle (DMSO) for 48 h or transfected with an independent sequence targeting EZH2 (siEZH2) or CNTL (siControl) siRNAs for 48 h were collected, and cell lysates were blotted for various cell cycle proteins as indicated.

We have previously reported that PTEN physically interacts with CBX8, a component of polycomb repressive complex 1 (PRC1), and that both PTEN and CBX8 have an impact on histone modifications during mitosis. Specifically, we observe that H3K27me3 signals are undetectable in mitotic cells and that downregulation of either PTEN or CBX8 results in a significant induction of H3K27me3^13^, suggesting that PTEN and CBX8 suppress this epigenetic modification during mitosis. Given that EZH2, a major component of PRC2, plays a significant role in modifying H2K27me3^34^, we determined whether EZH2 and other PRC components were post-translationally modified during mitosis. We noticed that EZH2 and BMI1, but not SUZ12 and Ring1A, displayed slow-mobility bands on denaturing gel, which coincided with increased phospho-H3 a mitotic marker, suggesting their post-translational modifications during mitosis (Fig. 1A, marked by arrow Modified). Moreover, we observed that H3K9me3 signals were low in mitosis, inversely correlated with phospho-H3 (p-H3, Fig. 1A). Consistent with previous findings, H3K9me3 exhibits lower expression during mitosis due to likely to masking by p-H3^35^. The observed modifications are therefore strongly associated with cells undergoing mitosis. These findings prompted us to further study the role of EZH2 in regulating cell cycle progression.

The cell cycle is a highly regulated process that comprises a cascade of cyclins (cyclin D, E, A, and B) that oscillate in expression throughout the cell cycle, reaching peaks at specific points. Cyclins bind to specific CDKs, to form cyclin-CDK complexes, which activate and subsequently phosphorylate target proteins, leading to the cell cycle progression^36^. This process is regulated by CKIs, such as the Cip/Kip family proteins p21, p27, and p57, preventing cell-cycle progression by inhibiting the catalytic activity of cyclin-CDK complexes^37^. EZH2, a major epigenetic modulator, is known to play a role in regulating the cell cycle^32,33^. To further determine the underlying mechanism of EZH2 in cell cycle progression, we transfected HeLa cells with specific EZH2 siRNAs in the presence or absence of nocodazole, a mitotic blocker. We observed that EZH2 knockdown induced expression of CDK inhibitors (CKIs) including p21, p27 and p57 (Fig. 1B, marked by *). Although nocodazole treatment completely suppressed expression of the CIP/KIP family proteins, EZH2 knockdown largely prevented the reduction of these proteins (Fig. 1B). Expression of various cyclins was also affected by EZH2 knockdown. In particular, after nocodazole treatment, EZH2 knockdown retained cyclin E2 but decreased cyclin B1 (Fig. 1B). This outcome contrasts with the expected reduction in cyclin E and increased expression of cyclin B1, a mitotic marker typically observed in nocodazole-treated cells. We conducted a CCK-8 assay to determine the number of viable cells. We observe that EZH2 knockdown, but not its enzymatic inhibition, decreased cell viability (Fig. S1). Flow cytometric analysis showed that increased expression of CKIs after knockdown of EZH2 was associated with a reduced S phase cell population with a concomitant increase of “G2/M-like” cells (Fig. 1C and D). Given the cyclin B level was greatly reduced which was coupled with high levels of cyclin D and cyclin A2 (Fig. 1B), we reasoned that these “G2/M-like” cells are tetraploid/polyploid with G1 characteristics. To eliminate the possibility that the observed upregulation in the expression of CKIs is a result of an off-target effect, we employed alternative sequences of siRNAs targeting EZH2. Again, EZH2 knockdown led to upregulated expression of CKIs, including p21, p27, and p57 (Fig. 1E). To further confirm the effect of EZH2 knockdown, we determined expression of c-Myc in cells transfected with EZH2 siRNAs as it has been shown that EZH2 physically interacts with c-Myc and contributes to its stabilization, independently of its methyltransferase activity^30^. Consistent with the report, EZH2 knockdown is associated with the reduction of c-Myc expression (Fig. 1E).

**Figure 2:**
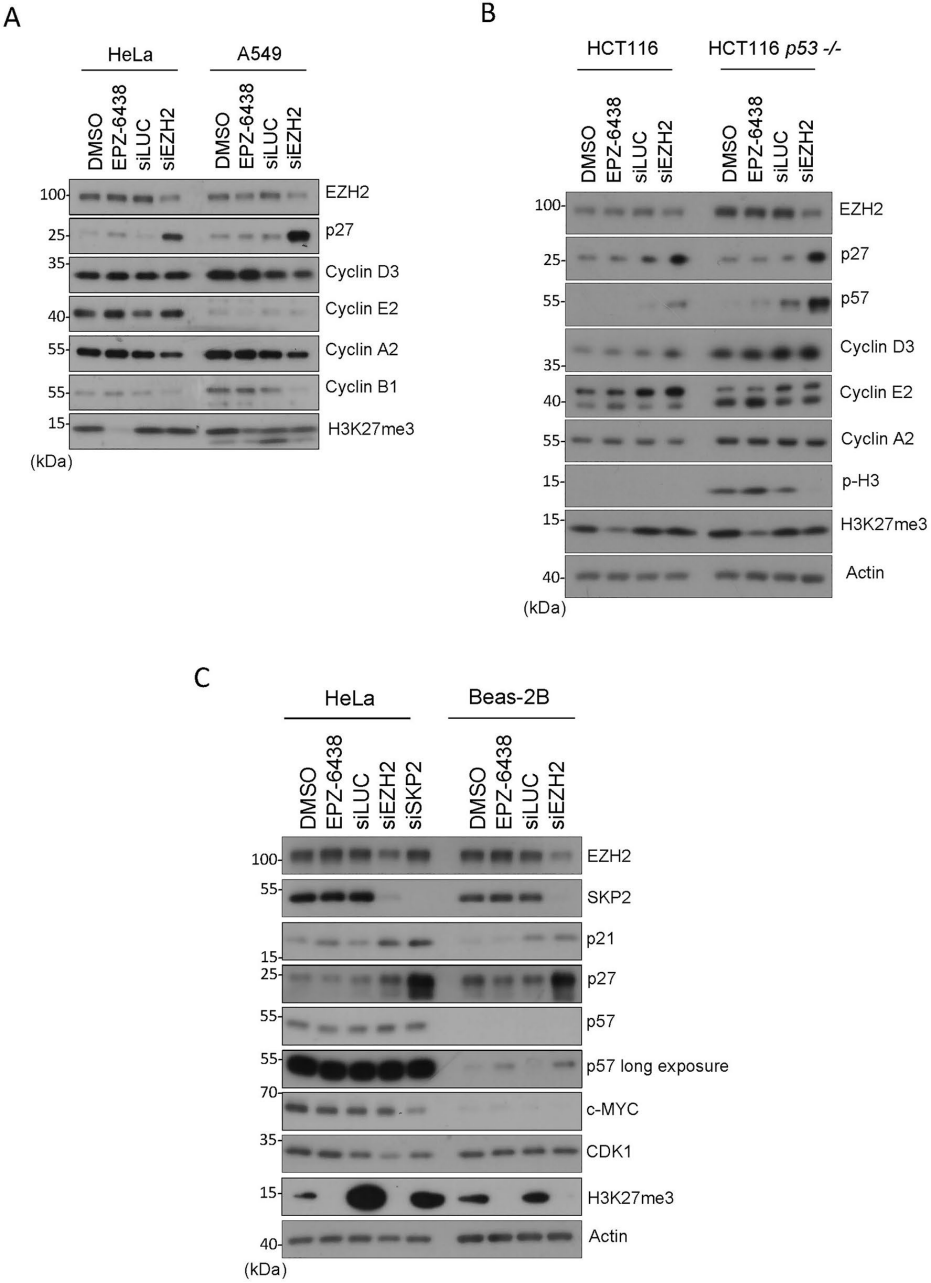
EZH2 knockdown induces expression of KIP family proteins in various cell lines. (**A**) HeLa and A549 cells were treated with EPZ-6438 (1 µM) or DMSO for 48 h or transfected with EZH2, or control (siLuc), siRNAs for 48 h. Cell lysates were then blotted for EZH2, p27, cyclin D3, cyclin E2, cyclin A2, cyclin B1, and H3K27me3. (**B**) HCT116 (WT or p53-null) cells were treated with EPZ-6438 (1 µM) or vehicle, or transfected with EZH2, or control (siLuc), siRNAs for 48 h. Cell lysates were blotted for various cell cycle markers as shown. (**C**) HeLa and non-transformed BEAS-2B cells were treated with EPZ-6438 (1 µM) or DMSO for 48 h or transfected with EZH2, SKP2 or control (siLuc), siRNAs for 48 h. Cell lysates were then blotted for various proteins as shown.

To eliminate the possibility that induction of CKI expression after knockdown of EZH2 was specific to HeLa cells, we also carried out the same experiments in other cell types. We observed that induced EZH2 knockdown induced expression of p27 in A549 (lung carcinoma) and HCT116 (colon carcinoma) cells (Fig. 2A and B). On the other hand, the absence of p53 did not affect the induction of p27 after EZH2 knockdown whereas it boosted the induction of p57 (Fig. 2B). Significantly, inhibition of EZH2 enzymatic activity via treatment with EPZ-6438 did not induce expression of p27 in HeLa, A549 or HCT116 cells (Fig. 2A and B). Likewise, EZH2 inhibition did not induce p57 in HCT116 in the presence or absence of p53 (Fig. 2B). EPZ-6438 was effective because H3K27me3 signals were either abolished or significantly reduced after treatment (Fig. 2A and B).

To determine whether the enzyme-independent role of EZH2 in CKI expression also occurred in non-transformed cells, we carried out these experiments in BEAS-2B cells. We showed that EZH2 knockdown, but not its enzymatic inhibition, resulted in an upregulation of p21, p27, and p57 (Fig. 2C). One major enzyme regulating the stability of KIP family proteins is SKP2^38^. As expected, SKP2 knockdown via siRNAs led to upregulation of p21, p27, and p57 (Fig. 2C). Therefore, these combined results strongly suggest that EZH2 regulates the KIP family proteins via an enzyme-independent mechanism.

Taken together, our results strongly suggest that the primary effect of EZH2 knockdown is cell cycle arrest. On the other hand, we need to bear in mind that EZH2 is a major epigenetic modulator. Thus, an alteration of EZH2 expression could potentially disrupt molecular processes, indirectly modulating expression of cyclins and CKIs, and causing cell cycle arrest.

### EZH2 knockdown, but not its inhibition, downregulates SKP2

**Figure 3:**
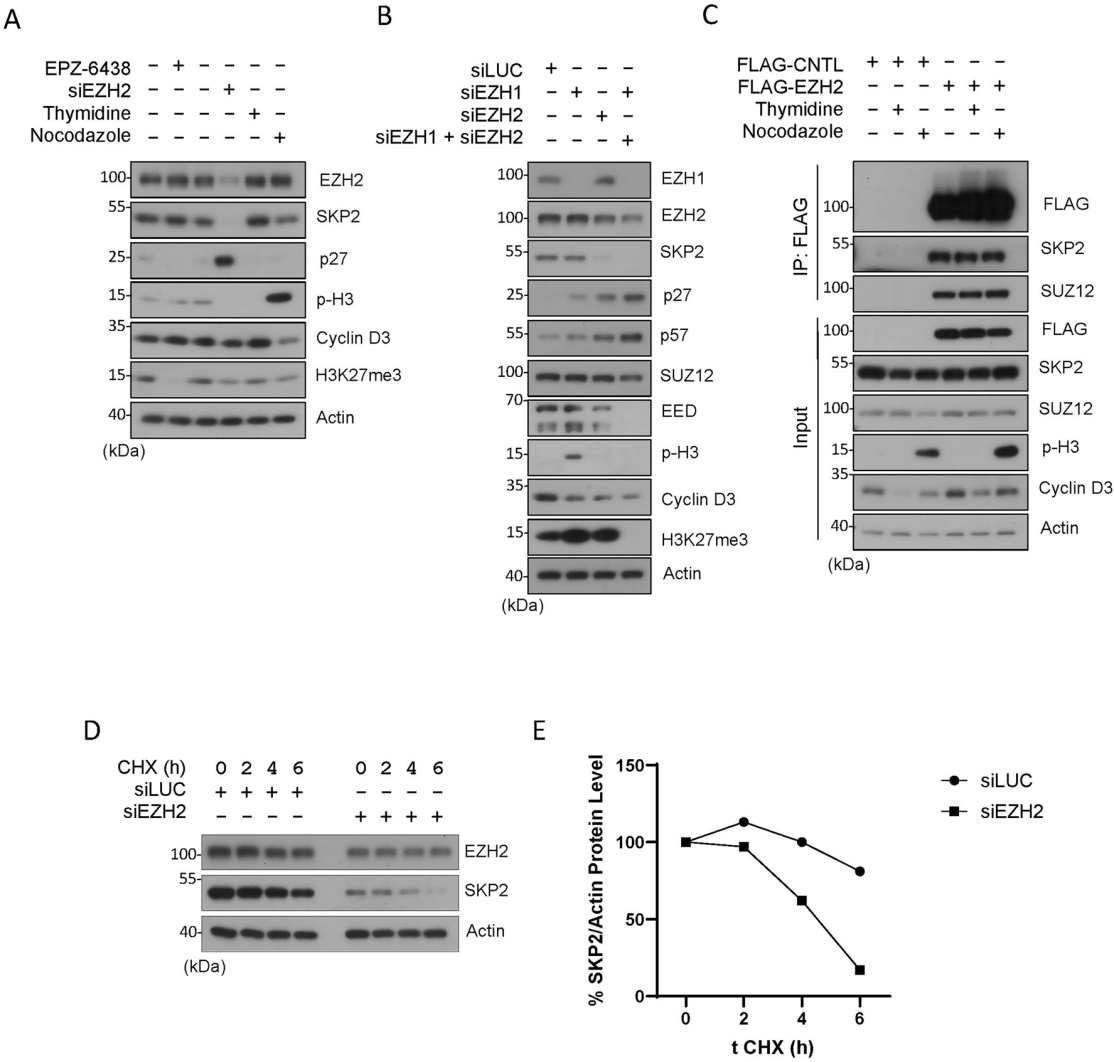
EZH2 knockdown but not enzymatic inhibition modulates SKP2 protein expression. (**A**) HeLa cells were treated with EPZ-6438 (1 µM) or transfected with EZH2 siRNAs (siEZH2) for 48 h, or synchronized with the thymidine block or by nocodazole treatment for 18 h. Cell lysates were then blotted for various cell cycle markers as shown. (**B**) HeLa cells were transfected with EZH1 siRNAs or EZH2 siRNAs or both for 48 h. Equal amounts of cell lysates were then blotted for polycomb group proteins (EZH1, EZH2, SUZ12 and EED), histone modifications (p-H3 and H3K27me3), and cell cycle markers including SKP2, p27 and p57. (**C**) HeLa cells were transfected with FLAG-tagged EZH2 or vector control (CNTL) for 24 h. Asynchronized cells or cells synchronized by thymidine block (G1/S) or treatment with nocodazole (M) were collected and lysed. Equal amounts of cell lysates were immuno-precipitated with the anti-FLAG antibody. Immunoprecipitants, along with cell lysate inputs of various treatments, were blotted for FLAG, SKP2, SUZ12, phospho-H3, cyclin D3, and β-actin. (**D**) HeLa cells transfected with EZH2 or CNTL (siLuc) siRNAs for 24 h. Cells treated with 50 µg/ml of cycloheximide (CHX), a protein-synthesis inhibitor, were collected at the indicated times. Cell lysates were blotted for EZH2 and SKP2 as indicated. (**E**) The graph reflects EZH2 and SKP2 band density relative to the β-actin level quantified by ImageJ.

SKP2, or S-phase kinase-associated protein 2, is an F-box protein that plays a critical role in regulating cell cycle progression, particularly during the transition from the G1 to the S phase^39^. The primary function of SKP2 is to mediate the degradation of several key cell cycle regulatory proteins, including p21, p27 and p57^38^. Thus, we determined whether EZH2 inhibition or knockdown would modulate expression of SKP2. Surprisingly, we noted that knockdown of EZH2 greatly suppressed expression of SKP2, which was associated with induction of p27 (Fig. 3A). However, inhibition of EZH2 by treatment with EPZ-6438 neither suppressed SKP2 nor induced p27 (Fig. 3A). As expected, EPZ-6438 completely abolished H3K27me3. These observations again indicate an enzyme-independent function of EZH2 in the regulation of SKP2 and CIP/KIP family CKIs. They are also consistent with our observations that EZH2 knockdown induced G2/M-like cells (Fig. 1C and D) as it is well known that SKP2 depletion induces tetraploid and polyploid cells both in vivo and in vitro^40,41^.

### EZH1 does not have a major role in expression of SKP2 and CKIs

Since EZH1 and EZH2 are highly homologous proteins that share several key similarities and functions^1,42^. Specifically, EZH1 and EZH2 catalyze the trimethylation of histone H3 on lysine 27 (H3K27me3)^34^. Thus, we determined whether EZH1 may also regulate SKP2 and KIP family proteins. Knockdown of EZH2, but not EZH1, upregulated expression of p27 and p57, which was associated with downregulation of SKP2 (Fig. 3B). Knockdown of EZH1 was very efficient compared with EZH2, thus strongly suggesting the absence of a role in regulating SKP2, as well as p27 and p57. Knockdown of both EZH1 and EZH2, but not individually, completely abolished H3K27me3 signals (Fig. 3B), implying the compensatory enzymatic mechanism between them within the PRC2 group. We also observed a high expression of p-H3 protein, a marker for mitotic cells. No direct link has been previously documented between EZH1 and p-H3 expression. Nevertheless, previous findings demonstrated that EZH1 exhibits high expression in mitotic germs and, in mice, plays a crucial role in differentiation and postmitotic cells, activating myogenic genes during skeletal muscle differentiation^43,44^. Moreover, genes upregulated in *EZH1*^*−/−*^ neuronal cells were significantly associated with gene ontology terms related to mitosis^44^. The observed high expression of p-H3 protein suggests a potential role of EZH1 in mitotic cells; further exploration is needed to uncover its specific functions in this context.

As it remains unknown whether the enzyme-independent regulation of SKP2 by EZH2 is mediated through the protein–protein interaction, we next determined whether EZH2 was physically associated with SKP2 during the cell cycle. Pull-down analysis revealed that ectopically expressed EZH2 specifically interacted with SKP2, as well as SUZ12 a known binding protein partner from the PRC2 (Fig. 3C). The interaction between EZH2 and SKP2 was strong as FLAG-EZH2 significantly enriched SKP2 in the immunoprecipitants as compared with the cell lysate unput (Fig. 3C). On the other hand, there was no significant difference in their interaction during interphase and mitosis as seen in thymidine and nocodazole treatments, respectively (Fig. 3C).

EZH2 knockdown by two independent sequences of siRNAs caused downregulation of SKP2 protein (Fig. 1B and E). To investigate if EZH2 could promote the SKP2 protein stability, a cycloheximide (CHX) chase time-course experiment was performed. It was found that EZH2 knockdown by siRNAs shortened the half-life of SKP2 protein in HeLa cells (Fig. 3D–E). Consistent with this observation, ectopic expression of EZH2 led to elevated expression of the SKP2 protein (Figs. S3A–B).

### EZH2 knockdown downregulates SKP2 mRNA, which is associated with EMT

**Figure 4:**
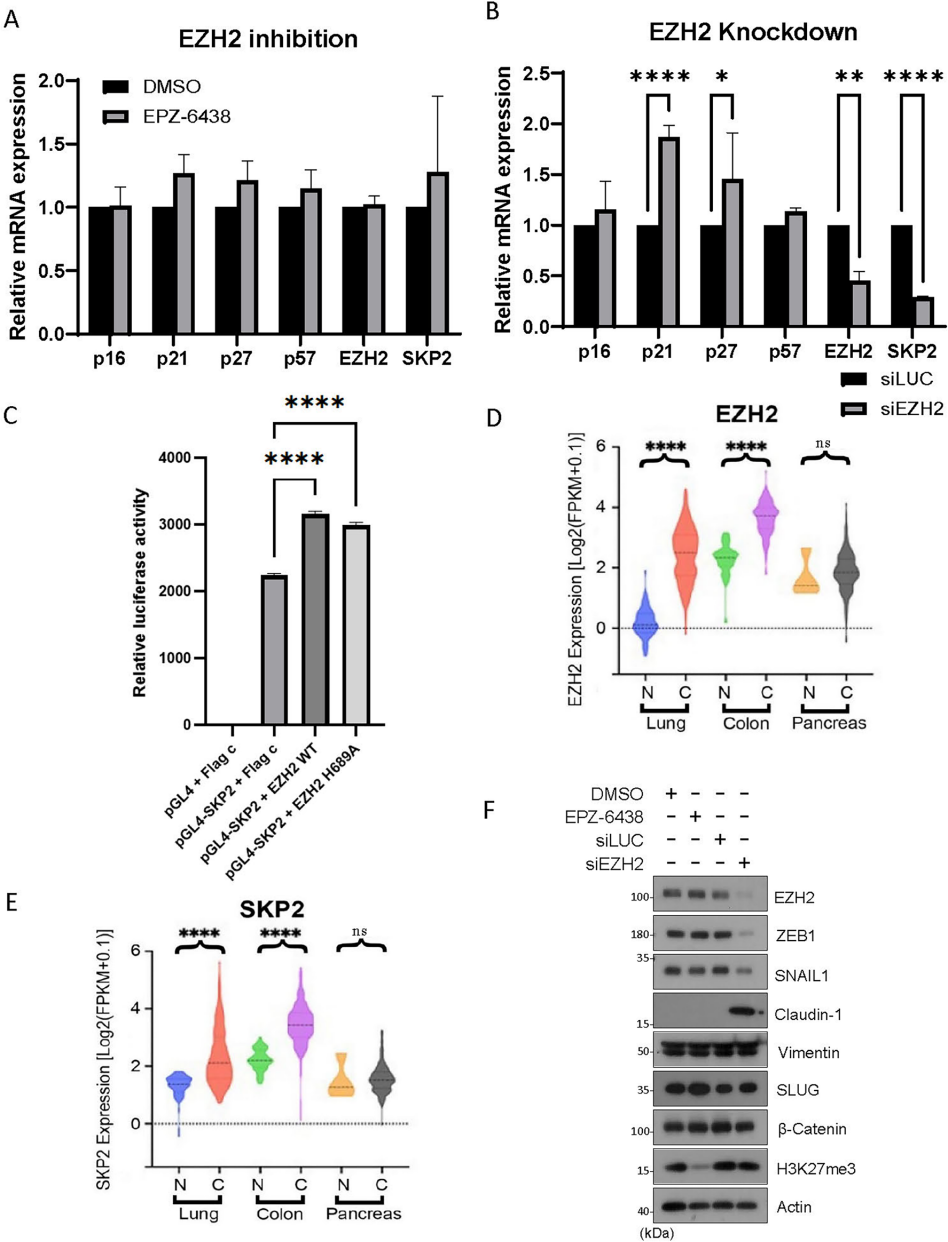
EZH2 knockdown downregulates SKP2 mRNA and modulates EMT. (**A**) HeLa cells were treated EPZ-6438 (1 µM) or vehicle (DMSO) for 48 h or (**B**) transfected with EZH2 or control siRNA (siLuc) for 48 h. Total RNAs were extracted from cells of both experiments and subjected to quantitative PCR analysis. Genes being analyzed include p16, p21, p27, and p57, as well as EZH2 and SKP2. Significance was analyzed using PRISM software as described in Methods. Error bars indicate triplicates. (**C**) Transient luciferase assay detection of the activity of the SKP2 promoter. HEK293T cells were co-transfected with the pGL4 construct containing the full‑length SKP2 promoter driving the luciferase gene and EZH2 WT or EZH2^H689A^. Following 24 h transfection, luciferase activity was determined. All data shown are the mean ± SEM from experiments performed in triplicate. (**D** and **E**) Violin plots reporting the relative expressions of EZH2 and SKP2 in paired adenocarcinomas (designated as **C**) or matched normal tissues (designated as N) among lung, colon and pancreatic cancer patients’ groups from the “Cancer Genome Atlas” database. *P* values were computed using the Wilcoxon rank sum test and adjusted using the Benjamini–Hochberg method. ****, adjusted *P* value < 0.0001; ns represents *P* > 0.05. (**F**) HeLa cells were treated EPZ-6438 (1 µM) or vehicle (DMSO) or transfected with EZH2 (siEZH2) or control (siLuc) siRNAs for 48 h. Equal amounts of cell lysates were then blotted with various EMT markers and β-actin.

EZH2 is known to epigenetically silence target genes. To determine whether EZH2 might transcriptionally influence SKP2 expression, we treated cells with EPZ-6438 or transfected them with EZH2 siRNAs or control siRNAs (siLuc) followed by analyzing expression of various relevant genes of interest by real-time PCR. We showed that inhibition of EZH2 by EPZ-6438 did not modulate expression of SKP2 gene or CKI genes including those coding for p16, p21, p27, and p57 (Fig. 4A). However, knockdown of EZH2 significantly suppressed SKP2 expression (Fig. 4B), indicating that EZH2 positively regulates SKP2 gene expression via a non-enzymatic function.

EZH2 has been shown to act as a transcriptional activator, inducing the expression of the androgen receptor gene in a methylation-independent manner^31^. As a result, we investigated the role of EZH2 in the transcriptional regulation of SKP2. A reporter gene construct consisting of luciferase gene driven by an SKP2 full-length promoter was generated and co-transfected with an expression construct of EZH2 or its enzymatic inactive mutant (EZH2^H689A^) into HEK293T cells. The results showed that both EZH2-WT and EZH2^H689A^ significantly increased SKP2 promoter activity, resulting in higher levels of SKP2 expression (Fig. 4C). It is observed that the basal level of SKP2 expression was rather high, which might have hindered the detection of the full promoting activity of EZH2 in this system. Nonetheless, our study suggests that EZH2 positively regulates SKP2 expression at the transcriptional level. Further supporting this, ectopic expression of EZH2 increased SKP2 protein levels (Fig. S3A–B).

Through the analysis of The Cancer Genome Atlas (TCGA) database, we identified a significant correlation between the expression levels of EZH2 and SKP2 in various human malignancies. In normal lung tissues, EZH2 expression was low; however, in both lung and colon cancers, it exhibited a substantial increase (Fig. 4D). Consistently, SKP2 expression was also significantly upregulated in lung and colon cancers (Fig. 4E). Notably, pancreatic cancer did not exhibit elevated expression of EZH2 (Fig. 4D), and similarly, there was no significant increase in SKP2 expression (Fig. 4E). These findings strongly suggest a close regulatory relationship between EZH2 and SKP2 in tumor development.

Increased expression of CKIs is frequently associated with cell differentiation. For example, it has been shown that the upregulation of p27 promotes cell neuronal cell differentiation^45^. In addition, it is well-established that a high expression of SKP2 can influence the modulation of EMT markers^22^. Elevated levels of SKP2 are associated with a more mesenchymal phenotype, while reducing its expression leads to a more epithelial phenotype^46^. We next asked whether EZH2-mediated suppression of CKIs is associated with EMT as cell cycle arrest or reduced cell proliferation in vivo is frequently associated with cell differentiation. We observed that knockdown of EZH2 led to a significant reduction in the expression of ZEB1 and SNAIL-1, which are major transcription factors associated with mesenchymal cells (Fig. 4F). However, the expression of SLUG, another key transcription factor in the EMT process, remained largely unaffected. Concurrently, we noted a substantial increase in the expression of Claudin-1, a marker typically associated with epithelial cells (Fig. 4F). On the other hand, treatment with EPZ-6438 did not exert any significant effects on the expression of ZEB1, SNAIL-1, or Claudin-1 (Fig. 4F). These observations further support the notion that EZH2's involvement in regulating EMT is not mediated through its enzymatic activity but instead involves enzyme-independent functions.

## Discussion

Our current study highlights the enzyme-independent regulation of SKP2 by EZH2. SKP2 is an F-box protein that plays a critical role in cell cycle progression by mediating the degradation of CKIs, including p21, p27, and p57. We demonstrate that knockdown of EZH2 leads to a significant downregulation of SKP2 expression, accompanied by the induction of p27. Importantly, this downregulation of SKP2 is not observed when EZH2 is inhibited using EPZ-6438, suggesting that the enzymatic activity of EZH2 is not involved in this process. These findings thus indicate that EZH2 exerts a non-catalytic role in stabilizing SKP2, which in turn affects the expression levels of CKIs. This enzyme-independent “stabilization” mechanism adds a new layer of complexity to the regulation of SKP2 and expands our understanding of EZH2's multifaceted functions in the cell cycle. Supporting this notion, EZH2 directly binds with MYC oncoprotein, promoting its stabilization independently of its methyltransferase activity^30^. We have provided evidence showing that EZH2 directly interacts with the SKP2 protein, and downregulation of EZH2 resulted in a shortened half-life of SKP2, suggesting that EZH2 might be involved in stabilizing the SKP2 protein. However, further studies are needed to elucidate the exact mechanism by which EZH2 promotes the stabilization of the SKP2 protein. In addition to the potential stabilization of SKP2, our current study also highlights the enzyme-independent transcriptional activation of SKP2 by EZH2. We demonstrate that knockdown of EZH2 significantly suppresses SKP2 mRNA expression, whereas inhibition of EZH2 with EPZ-6438 does not have the same effect. We have also provided evidence that EZH2-WT and EZH2 mutant significantly increases the transcriptional activation of SKP2. This observation suggests that EZH2 positively regulates SKP2 gene expression through a mechanism that is independent of its methyltransferase activity. Supporting this notion, EZH2 can act as a transcription activator, independent of its polycomb- and methylation-associated roles^31^. It’s important to note that the transcriptional activation of SKP2 by EZH2 might imply the involvement of additional molecular interactions or signaling pathways. It is possible that EZH2 interacts with other transcriptional regulators or chromatin modifiers to facilitate the transcriptional activation of SKP2. Further investigation is needed to determine the exact mechanism of SKP2 transcriptional activation by EZH2. These findings highlight the intricate network of regulatory mechanisms through which EZH2 modulates SKP2 expression, extending beyond its canonical enzymatic function and providing a solid foundation for future work.

Moreover, our results reveal that the knockdown of EZH2 leads to the upregulation of CKIs and subsequently impacts the expression of EMT markers. Specifically, we observe a decrease in the expression of ZEB1 and SNAIL-1, accompanied by an increase in Claudin-1 levels. Importantly, these effects are independent of EZH2's enzymatic activity, as inhibition with EPZ-6438 does not replicate the observed changes. These findings provide valuable insights into the intricate functions of EZH2 and its role in regulating EMT, with implications for understanding the underlying mechanisms of cell differentiation and potential therapeutic interventions in diseases where EMT dysregulation occurs.

Overall, our current study presents novel insights into the enzyme-independent mechanisms by which EZH2 regulates the expression and stability of SKP2, as well as the downstream effects on CKIs. By emphasizing the significant role of EZH2 in the regulatory hierarchy, specifically in stabilizing and transcriptionally activating SKP2, we propose that EZH2 plays multifaceted roles in cell proliferation and differentiation. Our findings open up new avenues for further research into the intricate molecular mechanisms underlying EZH2-mediated regulation and highlight the potential therapeutic implications for targeting EZH2 in diseases where dysregulation of cell cycle progression occurs.

## Methods

### Cell culture and treatments

HeLa (cervical carcinoma), A549 (lung carcinoma) and BEAS-2B (human non-tumorigenic lung epithelial) cell lines were obtained from the American Type Culture Collection. HCT116 cells (wild-type and p53-null) were obtained from Dr. Vogelstein at Johns Hopkin University. These cells cultured in DMEM supplemented with 10% fetal bovine serum (FBS, Invitrogen) and antibiotics (100 µg/ml of penicillin and 50 µg/ml of streptomycin sulfate, Invitrogen) at 37 °C under 5% CO_2_. In some experiments, cells were treated with EPZ-6438 (Tazemetostat, Selleck, S7128) (1 µM) or with the vehicle (DMSO) (Fisher BioReagents, 67-68-5).

### siRNAs and transfection

Human ON-TARGETplus SMARTpool oligonucleotides that specifically target EZH2 and EZH1 were purchased from Dharmacon. Their sequences are as follows: 5′-AAGAGGUUCAGACGAGCUGAUUU-3′ and 5′- AAAUCAGCUCGUCUGAACCUCUU-3′ (EZH2 sequence #1); 5′-CGGUGGGACUCAGAAGGCAGUUU-3′ and AAACUGCCUUCUGAGUCCCACCG-3′ (EZH2 sequence #2); 5′-CAGUUGCAUUGGUUCCCA and 5′-AUGGGAACCAAUGCAACUG3′ (EZH1) and 5′-GAAUCUUAGCGGCUACAGATT and 5′-UCUGUAGCCGCUAAGAUUCAG-3′ (SKP2). Pool of siRNAs were transfected into cells with Dharmafect I according to the protocol provided by the supplier. Briefly, cells seeded at 50% confluence in an antibiotic-free culture medium were transfected with siRNA duplexes at a final concentration of 100 nM for 24 h. siRNA controls which include two nucleotide changes from the target sequences were designed as described in previously^47^ and used as negative control for transfection.

### Cell cycle synchronization

HeLa cells were synchronized at the mitosis by thymidine-nocodazole block as described previously^15^. Briefly, cells were treated with 2 mM thymidine (Sigma-Aldrich) for 24 h followed by a 3 h release; these cells were then treated with nocodazole (50 ng/ml; Sigma-Aldrich) for another 18 h. Mitotic shake-off cells were obtained from gentle tapping of cell culture dishes. For nocodazole release, cells were washed three times with 1X PBS, then split onto cell culture dishes with complete medium. Cells were collected by various time point after release. Cells synchronized at mitosis were achieved by treatment with nocodazole for 16 h.

### Protein extraction and immunoblotting

Total cell lysates were prepared in a buffer [50 mM Tris-HCl (pH 7.5), 150 mM NaCl, 1% IGEPAL, 0.1% SDS, and 0.5% sodium deoxycholate] supplemented with a mixture of protease and phosphatase inhibitors. Protein concentrations were measured using the bicinchoninic acid protein assay reagent kit (Pierce Chemical Co). Equal amounts of protein lysates from various samples were used for SDS–PAGE analysis followed by immunoblotting. Antibodies against cyclin D3 (2936), cyclin E2 (4132), cyclin A2 (4656), cyclin B1(12231), p27 (3686), p57 (2557), SUZ12 (3737), EZH1, EZH2 (5246), BMI1(6964), Ring1A (13069), CBX8 (1,696), c-PARP (5625), phospho-H2AX (2577), H2AK119ub (8240), phospho-H3, H3K9me3 (13969), H3K27me3 (9733), SKP2 (2652), YKDDDDK (FLAG Tag) (#14793), ZEB1 (3396), Snail (3879), Claudin-1 (13255), Vimentin (5741), Slug (9585), β-Catenin (8480) and βActin (4970) were purchase from Cell Signaling Technology. Antibodies against p21 (sc-397), p27 (sc-1641), c-MYC (sc-40) and Cdc2 p34 (sc-54) were purchased from Santa Cruz biotechnology. After incubation with the horseradish peroxidase-conjugated secondary antibody rabbit (7074) or mouse (7076) (Cell Signaling Technology), specific signals on the membrane blots were revealed using the Chemiluminescence procedure (Sigma).

### Quantifications of western blots with ImageJ

For western blot quantification, ImageJ was used to measure the density of each band on the blots. All western blot data films are scanned with high resolution (300 dpi) and performed in ImageJ. The relative amount as a ratio of each band relative to the loading control was presented.

### Immunoprecipitation

Human EZH2 expression plasmid (pcDNA3) was purchased from Addgene containing the FLAG tag fused in-frame at the N-terminus of the protein (id #173717: pcDNA3.1_3xFlagEzh2 WT). Transfection of individual cell lines was achieved with either Lipofectamine 2000 (Invitrogen) or Fugene HD (Roche Diagnostics) following the manufacturers’ protocol. Transfection efficiency was estimated to be between 80 and 100% in all cases through co-transfecting a GFP expressing plasmid (Data not shown). Cells were lysed in TBSN buffer [20 mM Tris-Cl (pH 8.0), 150 mM NaCl, 0.5% NP-40, 5 mM EGTA, 1.5 mM EDTA, 0.5 mM Na_3_VO_4_, and 20 mM β-Glycerol phosphate]. The cell lysates were clarified by centrifugation at 15,000 × g for 20 min at 4 °C. Cleared lysates (1 mg) were added to FLAG M2 agarose (Sigma) followed by incubation in the TBSN buffer for 1 h at 4 °C. After incubation, resins were thoroughly washed with the binding buffer and proteins bound to resin eluted in the SDS-PAGE sample buffer. A fraction of eluted sample was also analyzed by SDS-PAGE.

### Cycloheximide chase assay

To visualize protein degradation, CHX (50 µg/ml) (ab120093) was treated, and cells were harvested at different time points followed by western blot analysis.

### Proliferation assay

HeLa cells were seeded in a 96-well plate at an appropriate density, followed by siRNA transfection or EZH2 inhibitor treatment. After 48 h, the CCK-8 (CK04-01, Dojindo) reagent was added, and absorbance was measured following the manufacturer's procedure.

### Flow cytometry

HeLa cells were transfected with EZH2 (siEZH2) or control (siLuc) siRNAs for 48 h or treated with EZH2 inhibitor EPZ-6438 (1 µM) (or with the vehicle) for 48 h, followed by staining with Click-it Plus EdU and FxCycle Violet Stains according to the protocol provided by the manufacturer (Sigma). Cell cycle distributions were evaluated by flow cytometry in conjunction with the Facility Core of the Medical School.

### RNA isolation and real-time qPCR

HeLa cells were treated with EPZ-6438 (1 µM) for 48 h or transfected with EZH2, or control, siRNAs for 48 h. Total cellular RNAs were extracted using TRIzol. Quantitative PCR was performed using the PowerUP SYBR Green Master Mix followed by analysis with QuantStudio Software (Thermo Fisher). Relative mRNA expression was calculated and normalized to GAPDH. Primers for qPCR analyses were as follows: (**p27)** 5′-TGGAGAAGCACTGCAGAGAC-3′ (forward) and 5′-GCGTGTCCTCAGAGTTAGCC-3′ (reverse); (p**57)** 5′-ATGTCCGACGCGTCCCTCC-3′ (forward) and 5′-CGAGTCGCTGTCCACTTCGG3′ (reverse); (**p16)** 5′-ATATGCCTTCCCCCACTACC-3′ (forward) and 5′-CATCATCATGACCTGGATCGC-3′ (reverse); (**EZH2)** 5′-GACCTCTGTCTTACTTGTGGAGC-3′ (forward) and 5′-CGTCAGATGGTGCCAGCAATAG-3′ (reverse); (**PRMT5)** 5′-CTAGACCGAGTACCAGAAGAGG-3′ (forward) and 5′-CAGCATACAGCTTTATCCGCCG3′ (reverse); (**p21)** 5′-AGGGGACAGCAGAGGAAG-3′ (forward) and 5′-GCGTTTGGAGTGGTAGAAATCTG-3′ (reverse); (**SKP2) 5′-**GATGTGACTGGTCGGTTGCTGT-3′ (forward) and 5′-GAGTTCGATAGGTCCATGTGCTG-3′ (reverse); (**GAPDH**) GTCTCCTCTGACTTCAACAGCG (forward) and ACCACCCTGTTGCTGTAGCCAA (reverse).

### Site-directed mutagenesis

pcDNA3.1_3xFlagEZH2 WT plasmid was used to generate the EZH2 enzymatic inactive mutant H689A. Primers used to generate the mutation: Forward: GTTTGGATTTACCGAAGCATTTGCAAAACGAATTTTGTTACCCTTGCG and Reversed: CGCAAGGGTAACAAAATTCGTTTTGCAAATGCTTCGGTAAATCCAAAC. The site-directed mutagenesis was performed using two parallel single-primer reactions following Edelheit et al., 2009 methodology article^48^. The mutation was confirmed by DNA sequencing (Genewiz, https://www.genewiz.com/).

### SKP2 promoter cloning and SKP2‑luciferase vector construction

A 1191 bp SKP2 promoter sequence insert was amplified from HeLa genomic DNA by PCR using Platinum Taq High Fidelity (Cat. No. 11304-011, Invitrogen) with SKP2 promoter primers Forward: ATTAGGTACCCCACTGTTTTCCCGGGGTCACA and Reversed: ATCTAAGCTTCTCGCCTCCCAGATTCCCGC. Primers were generated by the online designing tool Primer3. Restriction enzyme sites Hind III and KnpI were added to the primers. The vector with no promoter pGL4.17 (Cat. No. E6721, Promega) was used to insert the SKP2 promoter sequence. Digestion of DNA was performed with restriction enzymes Hind III (R3104S) and KnpI (R3142S) from New England Bio Labs following the manufacturer's protocol. Isolation of insert and vector performed by QIAquick Gel Extraction Kit (Cat. No. 28704, Qiagen). T4 DNA ligase (Cat. No. 15224-041, Invitrogen) was used for ligation following the manufacturer's protocol. For transformation, MAX Efficiency DH5α chemical competent cells (Cat. No. 18258012, Invitrogen) were used following the manufacturer's protocol. Plasmid extractions were performed by QIAprep Spin Miniprep kit (Cat. No.27106) or HiSpeed Plasmid Maxi Kit (Cat. No. 12663) from Qiagen following manufacturer protocol.

### Luciferase gene reporter assay

HEK293T cells were co-transfected, as mentioned above in the immunoprecipitation section. Dual-Luciferase Reporter Assay System (Cat. No. E1910, Promega) was used for cell lysate preparation and detection of luciferase activity following manufacture protocol. Luminescence measurements were carried out using a Luminoskan (Thermo Scientific) DLR equipped with Skanlt Software 6.1 RE for Microplate Readers, following the manufacturer's protocol. All measurements were normalized to pGL4 + Flag c control and performed in triplicate.

### The cancer genome atlas data retrieval and analysis

RNA-Seq data of cancer patients was obtained in The Cancer Genome Atlas (TCGA, https://portal.gdc.cancer.gov/) database using Qiagen OmicSoft Land Explorer version TCGA_B38_GC33) and graphed using GraphPad PRISM software. For each group, EZH2 and SKP2 expression were calculated as the average of Log2(FPKM + 0.1). Analysis of differential expression levels between patients’ groups was performed using the Wilcoxon rank sum test. Only data from patients’ groups with average abundances higher than 0.1% in at least one group and with Benjamini-Hochberg-adjusted *P* values of < 0.001 were considered statistically significant.

### Statistical analysis

Each experiment was performed at least three times. The data were plotted as the mean ± S.D. One-way ANOVA or Two-way ANOVA PRISM 10 software from GraphPad, San Diego, CA, was used for comparisons and is indicated as follows: *P ≤ 0.05; **P ≤ 0.01; ***P ≤ 0.001.

### Supplementary Information

Supplementary Legends.

Supplementary Figure S1.

Supplementary Figure S2.

Supplementary Figure S3.

Supplementary Figures.

## Data Availability

This study includes no data deposited in external depositories. The authors declare that all data supporting new findings in this article are available upon reasonable request.
