# Author Correction: Enzyme-independent role of EZH2 in regulating cell cycle progression via the SKP2-KIP/CIP pathway

**DOI:** 10.1038/s41598-024-68294-x

**Published:** 2024-07-27

**Authors:** Tania Colon, Ziyue Kou, Byeong Hyeok Choi, Franklin Tran, Edwin Zheng, Wei Dai

**Affiliations:** Division of Environmental Medicine, Department of Medicine Grossman School of Medicine, New York University, 341 East 25th Street, New York, NY 10010 USA

Correction to: *Scientific Reports* 10.1038/s41598-024-64338-4, published online 11 June 2024

In the original version of this Article, Edwin Zheng was omitted from the Author list.

The Author Contributions section now reads:

“T.C.: Data curation, formal analysis, writing; Z.K.: Data curation and project discussion; B.H.C.: Supervision, data analysis, discussion, writing and editing; F.T.: Editing and project discussion; E.Z.: Obtaining and confirming data; W.D.: Conceptualization, supervision, funding acquisition, writing and reviewing.”

The original Article has been corrected.

