# Supplementary material for: Enzyme-independent role of EZH2 in regulating cell cycle progression via the SKP2-KIP/CIP pathway
Source: Sci Rep. 2024 Jun 11;14:13389. doi: 10.1038/s41598-024-64338-4 (PMC11166936; doi:10.1038/s41598-024-64338-4)
Supplement: Supplementary file 1 — Supplementary Legends. [file 41598_2024_64338_MOESM1_ESM.docx]

**Legends For Supplemental Figures**

**Figure S1. Determination of the number of viable cells for cell proliferation.** (A) HeLa cells were plated in 96 well plate and treated with EPZ-6438 (1 µM) or DMSO for 48 h or transfected with siEZH2, or control (siLuc), siRNAs for 48 h. The plate was incubated for 1 hour with CCK8 solution, followed by measuring the absorbance at 450 nm.

**Figure S2. Transient overexpression of EZH2 at the transcriptional level.** (A) HeLa cells were transfected with EZH2 WT plasmid (FLAG-EZH2) or control (FLAG-CNTL) (10 ng) for 24 h. Total RNAs were extracted from cells of both experiments and subjected to quantitative PCR analysis. Genes being analyzed include p16, p21, p27, and p57, as well as EZH2, SKP2 and PRMT5 as a positive control.

**Figure S3. Ectopic Expression of EZH2 is associated with increased SKP2**. (A) HeLa cells transfected with FLAG-EZH2 WT or FLAG-CNTL for 24 h were collected, and cell lysates were blotted for various cell cycle proteins as indicated. Cell lysates were blotted for EZH2 and SKP2 as indicated. (B) The chart indicates EZH2 and SKP2 band density relative to that of β-actin quantified by ImageJ.

Figure 2
