# Supplementary figures and images for: Enzyme-independent role of EZH2 in regulating cell cycle progression via the SKP2-KIP/CIP pathway
Source: Sci Rep. 2024 Jun 11;14:13389. doi: 10.1038/s41598-024-64338-4 (PMC11166936; doi:10.1038/s41598-024-64338-4)

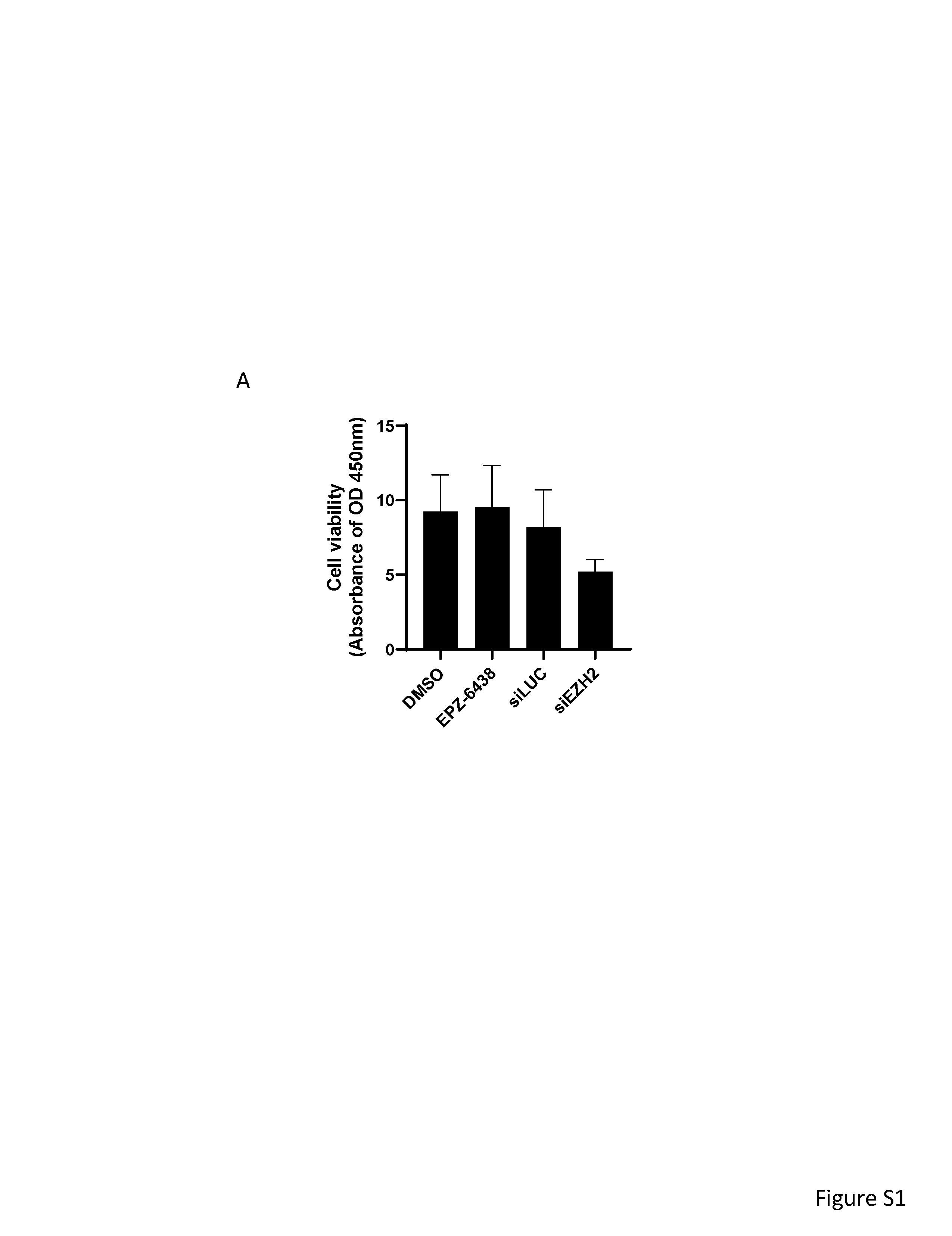

Supplement: Supplementary file 2 — Supplementary Figure S1. [file 41598_2024_64338_MOESM2_ESM.jpg]

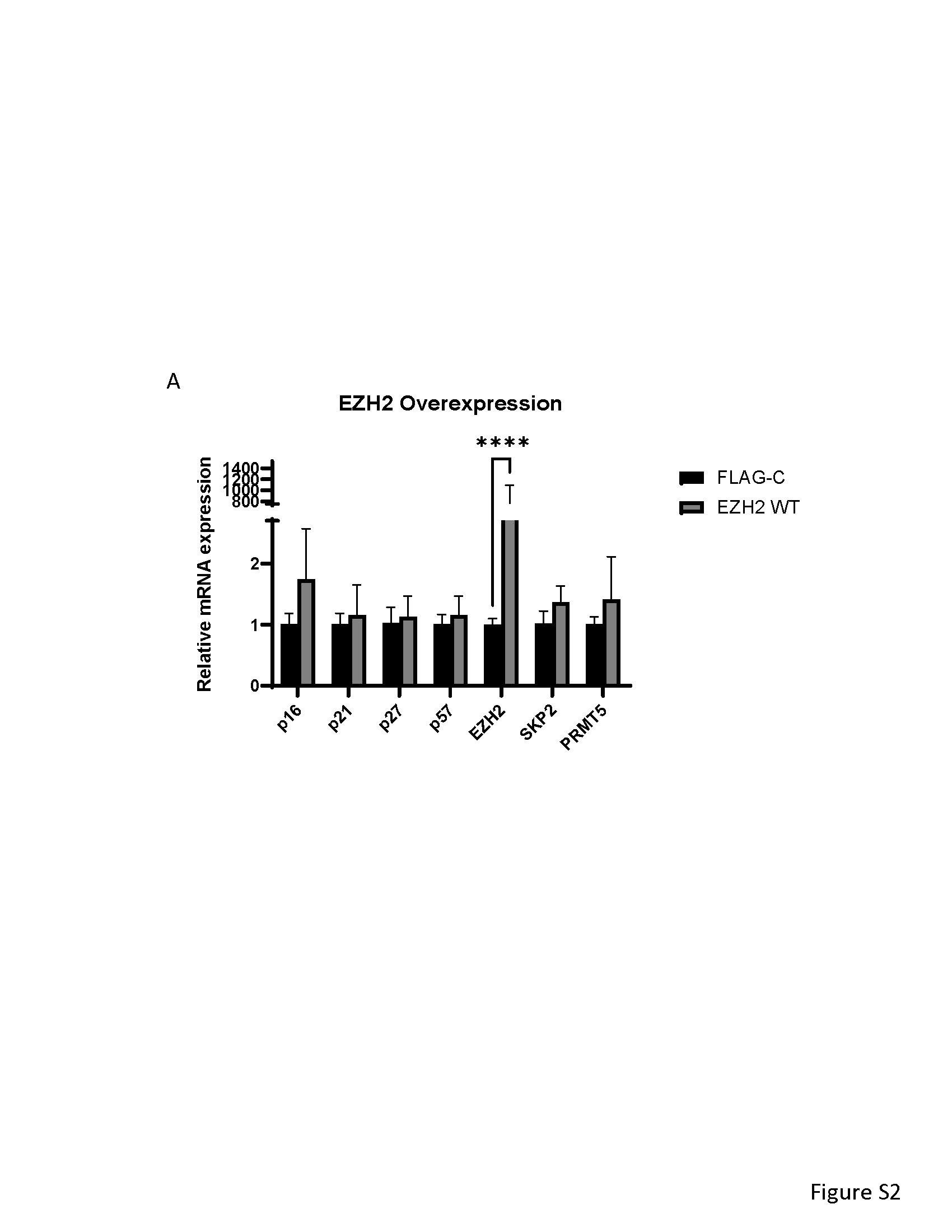

Supplement: Supplementary file 3 — Supplementary Figure S2. [file 41598_2024_64338_MOESM3_ESM.jpg]

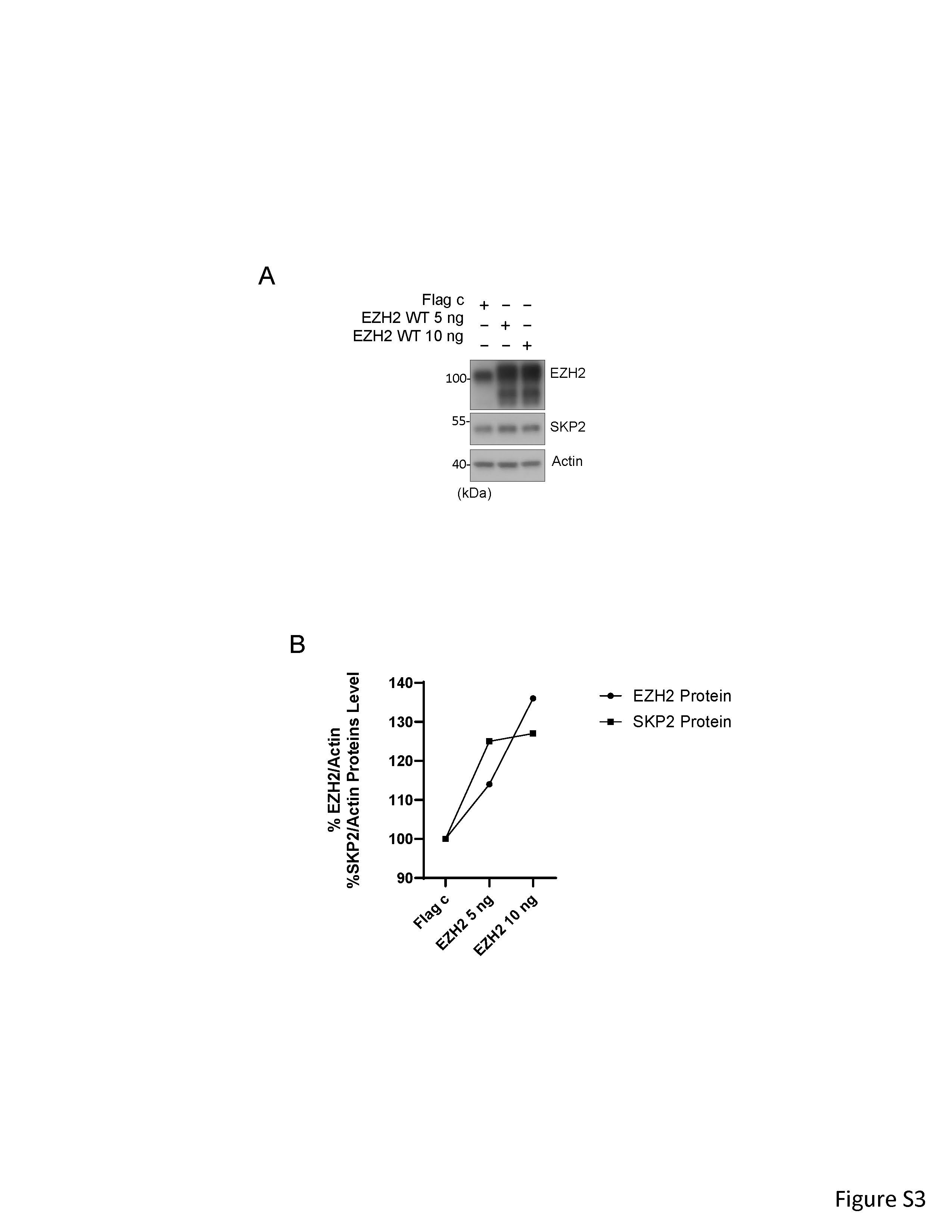

Supplement: Supplementary file 4 — Supplementary Figure S3. [file 41598_2024_64338_MOESM4_ESM.jpg]
