# Supplementary material for: Enzyme-independent role of EZH2 in regulating cell cycle progression via the SKP2-KIP/CIP pathway
Source: Sci Rep. 2024 Jun 11;14:13389. doi: 10.1038/s41598-024-64338-4 (PMC11166936; doi:10.1038/s41598-024-64338-4)
Supplement: Supplementary file 5 — Supplementary Figures. [file 41598_2024_64338_MOESM5_ESM.pdf]

Fig. 1A. Double thymidine block and release.
Exposure #2: proteins used in the manuscript from this exposure: Actin, BMI1, pH3.

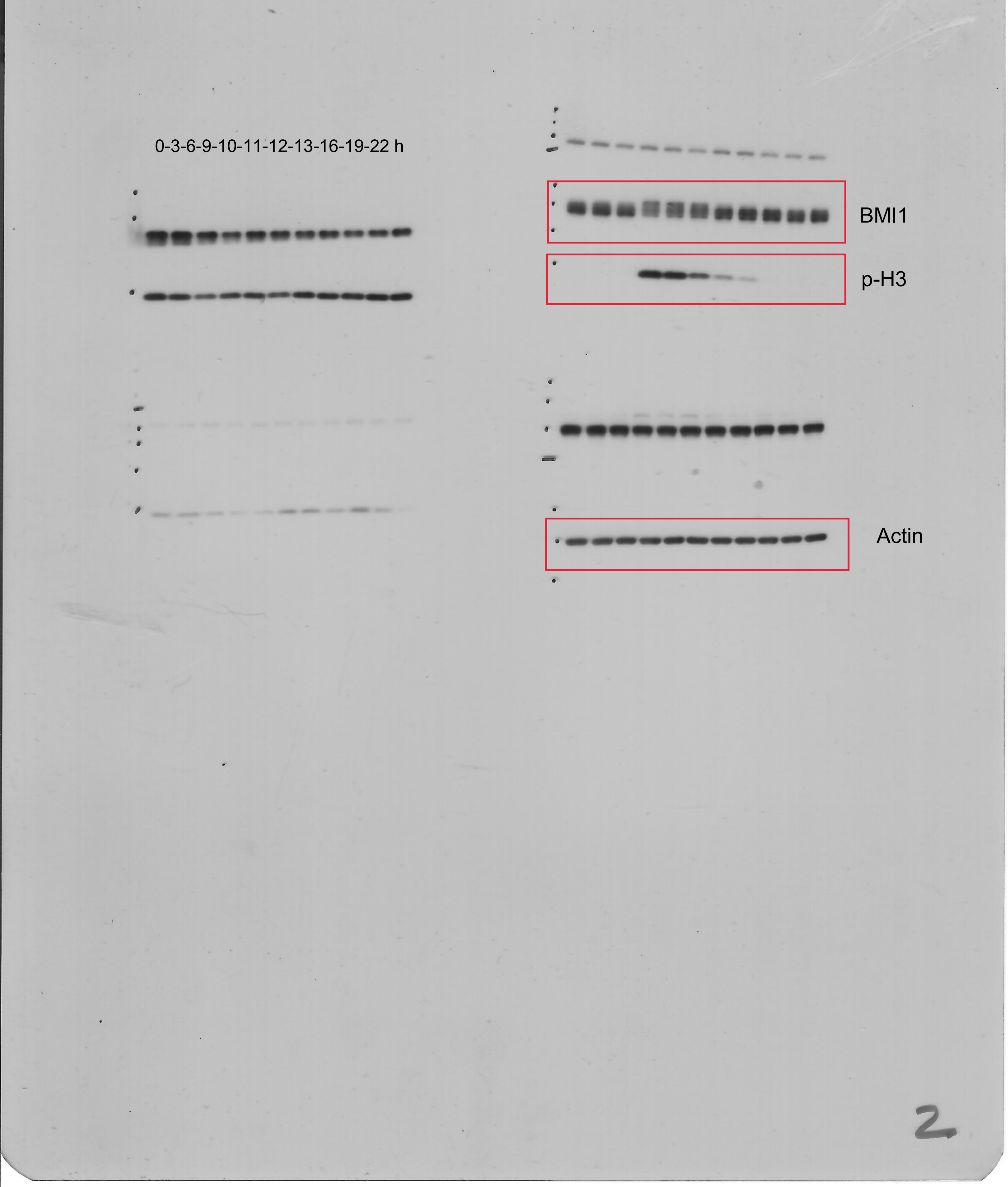

Fig. 1A. Double thymidine block and release.
Exposure #3: proteins used in the manuscript from this exposure: SUZ12, EZH2, H3K9me3.

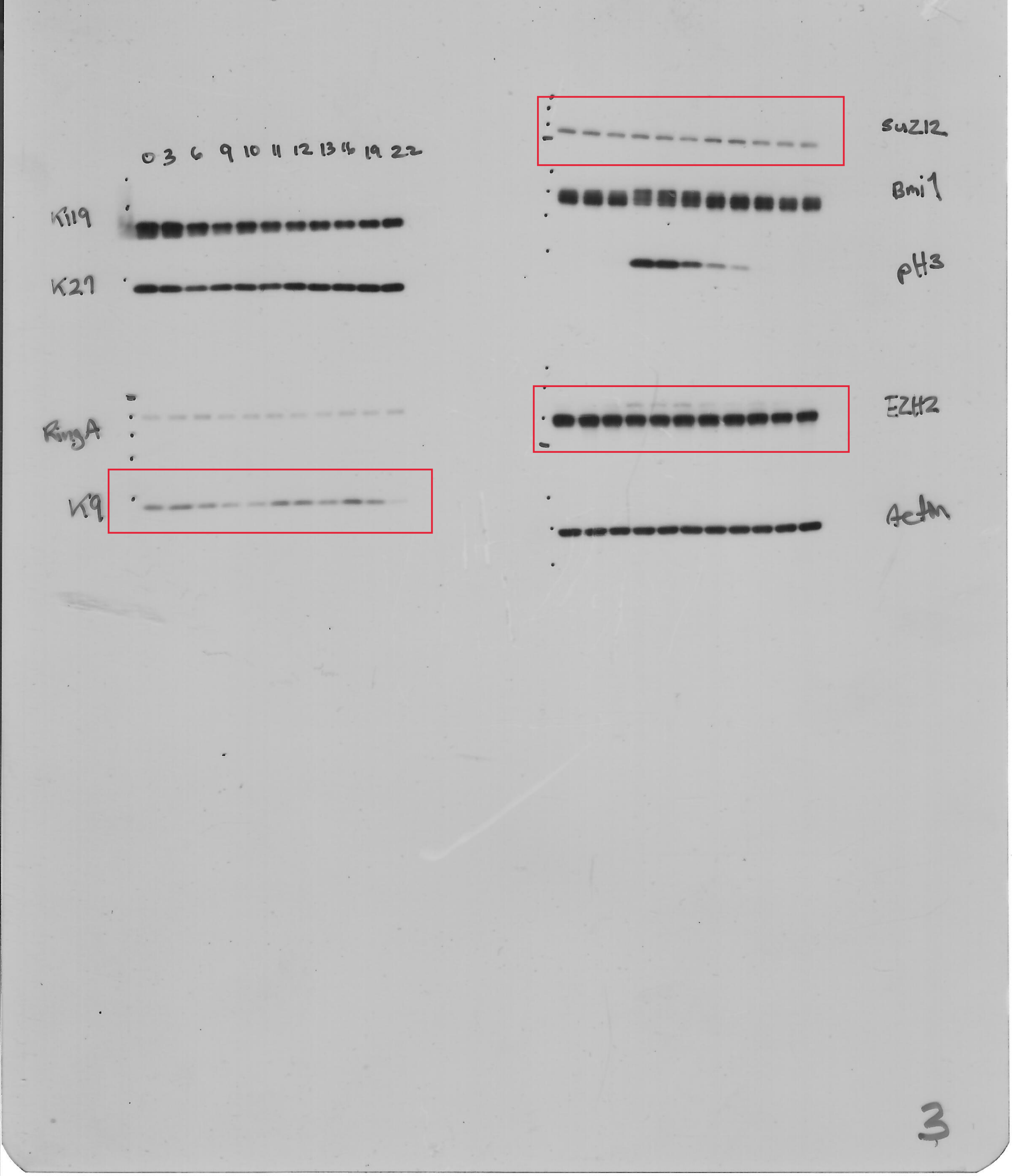

Fig. 1A. Double thymidine block and release.
Exposure #5: proteins used in the manuscript from this exposure: Ring1A.

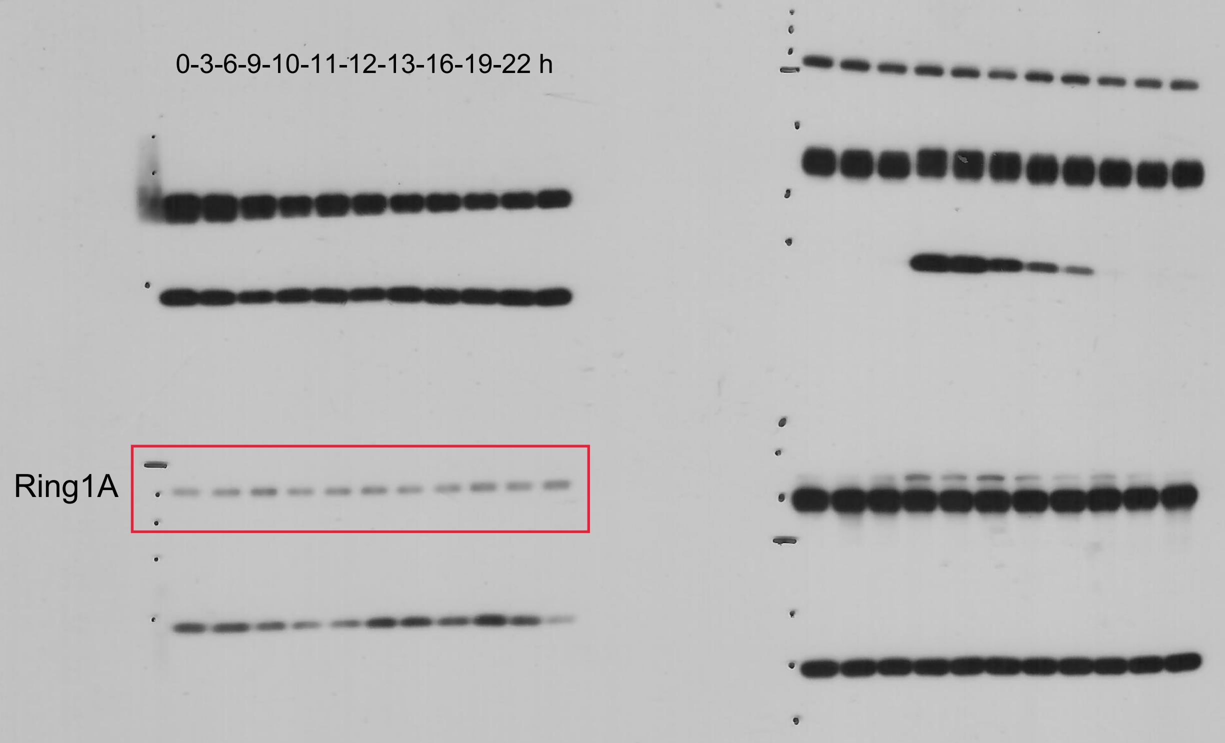

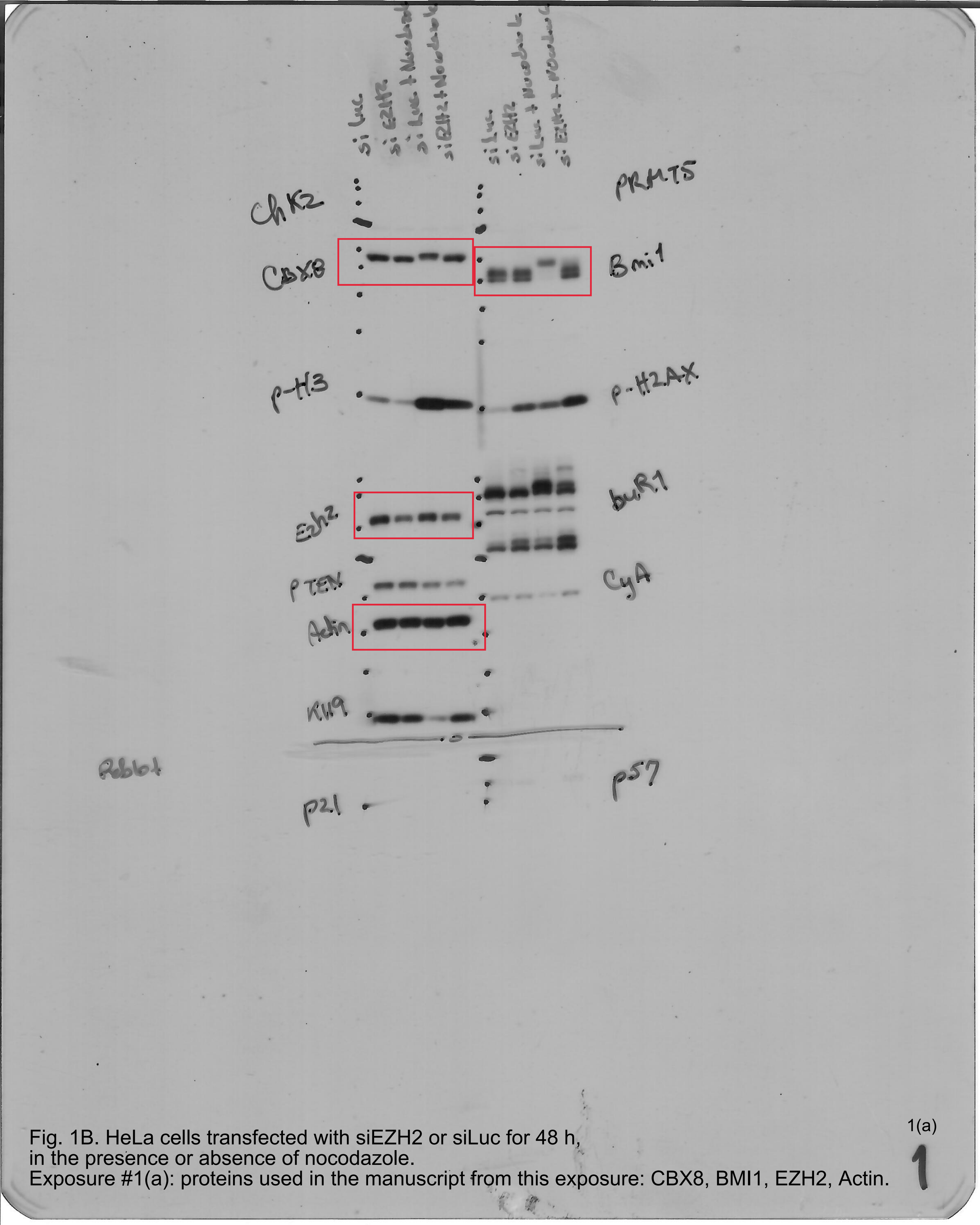

1(a)

Fig. 1B. HeLa cells transfected with siEZH2 or siLuc for 48 h,
in the presence or absence of nocodazole.
Exposure #1(a): proteins used in the manuscript from this exposure: CBX8, BMI1, EZH2, Actin.

Fig. 1B. HeLa cells transfected with EZH2 or siLuc for 48 h,
in the presence or absence of nocodazole.
Exposure #2(a): proteins used in the manuscript from this exposure: Cyclin A, p57.
Samples are ordered in the same way as blot #1(a).

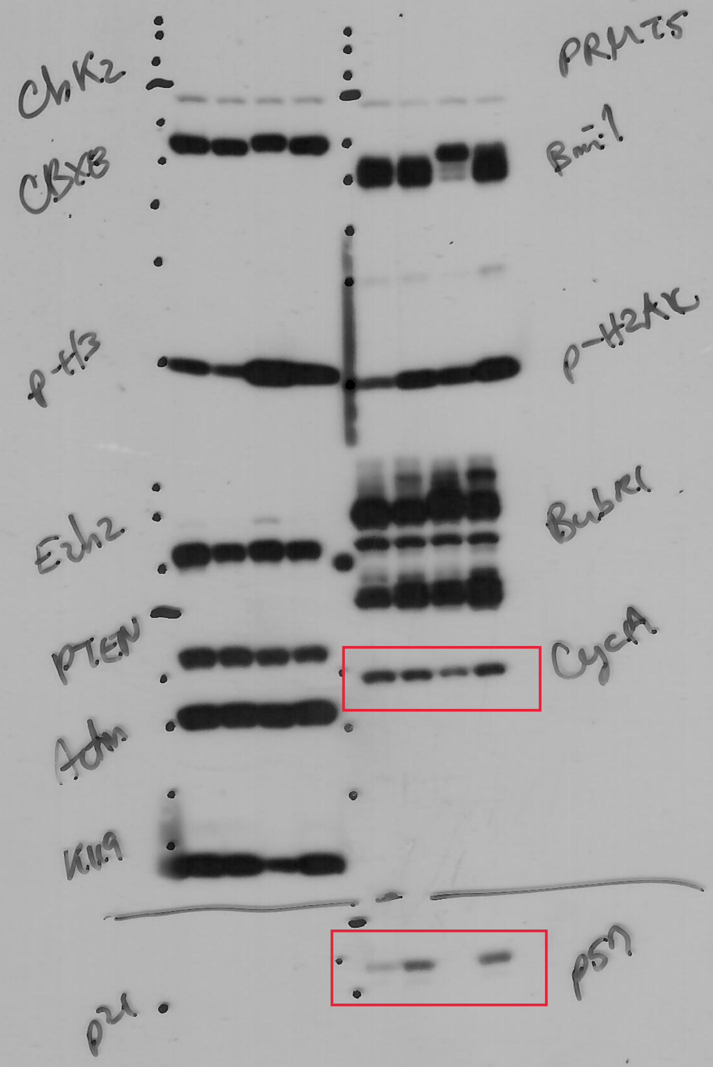

2(a)

Fig. 1B. HeLa cells transfected with EZH2 or siLuc for 48 h,
in the presence or absence of nocodazole
Exposure #3(a): proteins used in the manuscript from this exposure: none.
Samples are ordered in the same way as blot #1(a).

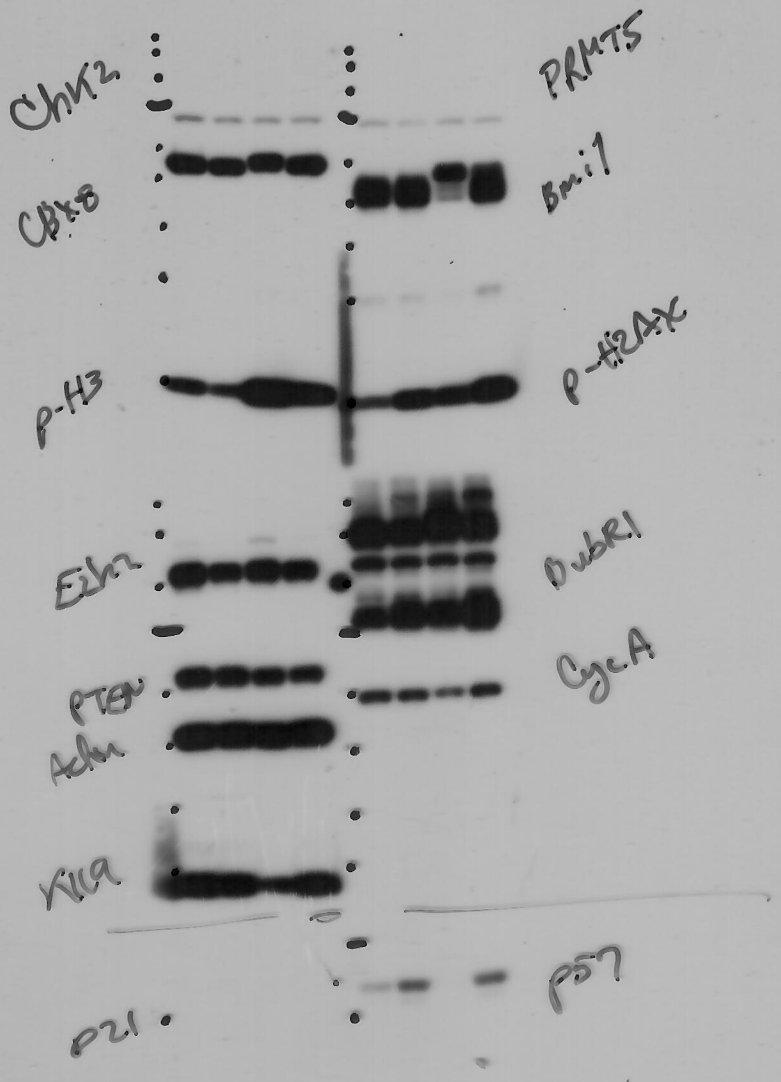

3(a)

3

Fig. 1B. HeLa cells transfected with EZH2 or siLuc for 48 h,
in the presence or absence of nocodazole.
Exposure #6(a): proteins used in the manuscript from this exposure: p21.
Samples are ordered in the same way as blot #1(a).

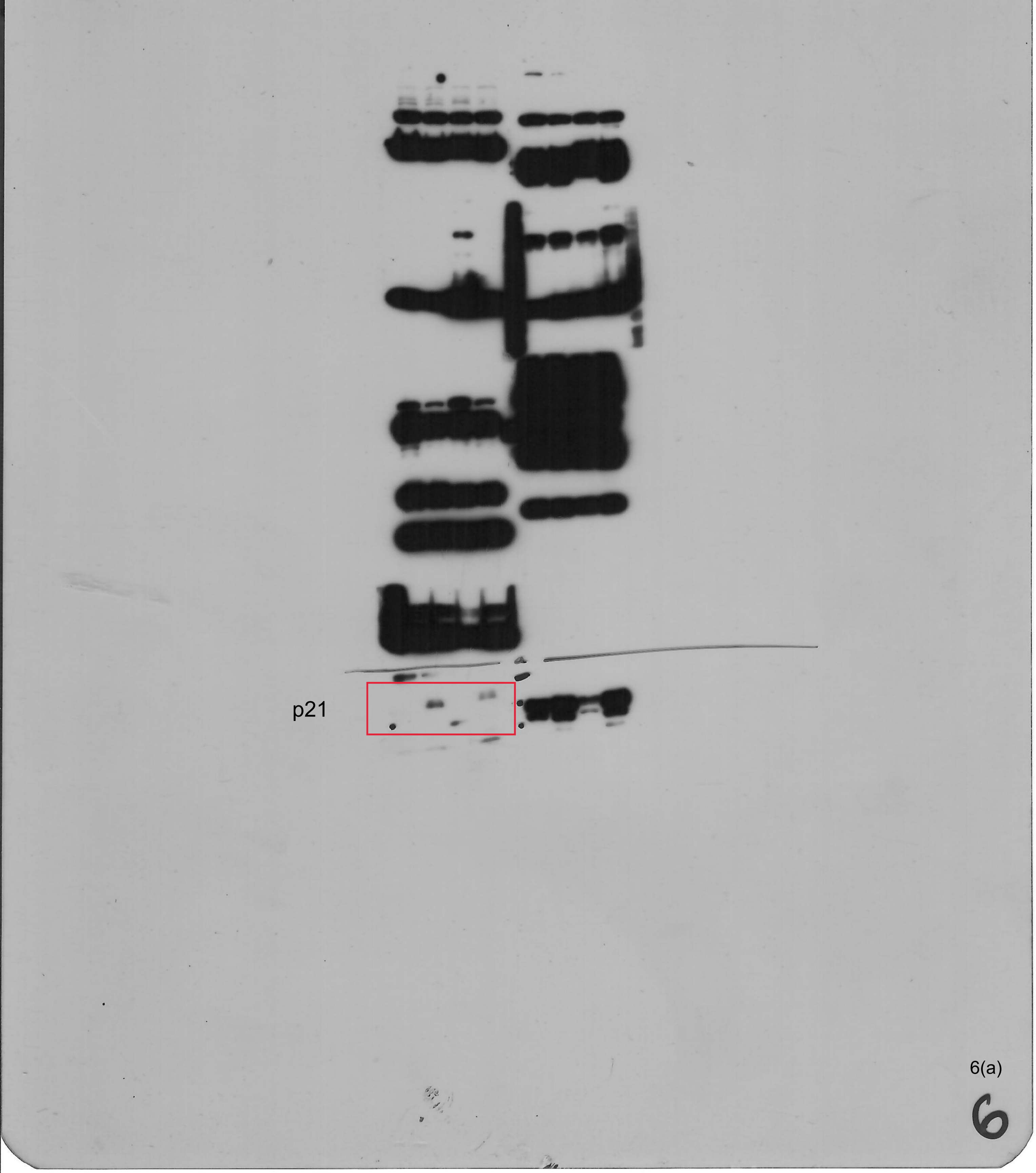

Fig. 1B. HeLa cells transfected with EZH2 or siLuc for 48 h,
in the presence or absence of nocodazole
Exposure #1(b): proteins used in the manuscript from this exposure: Cyclin D3, p27.
Samples are ordered in the same way as blot #1(a).

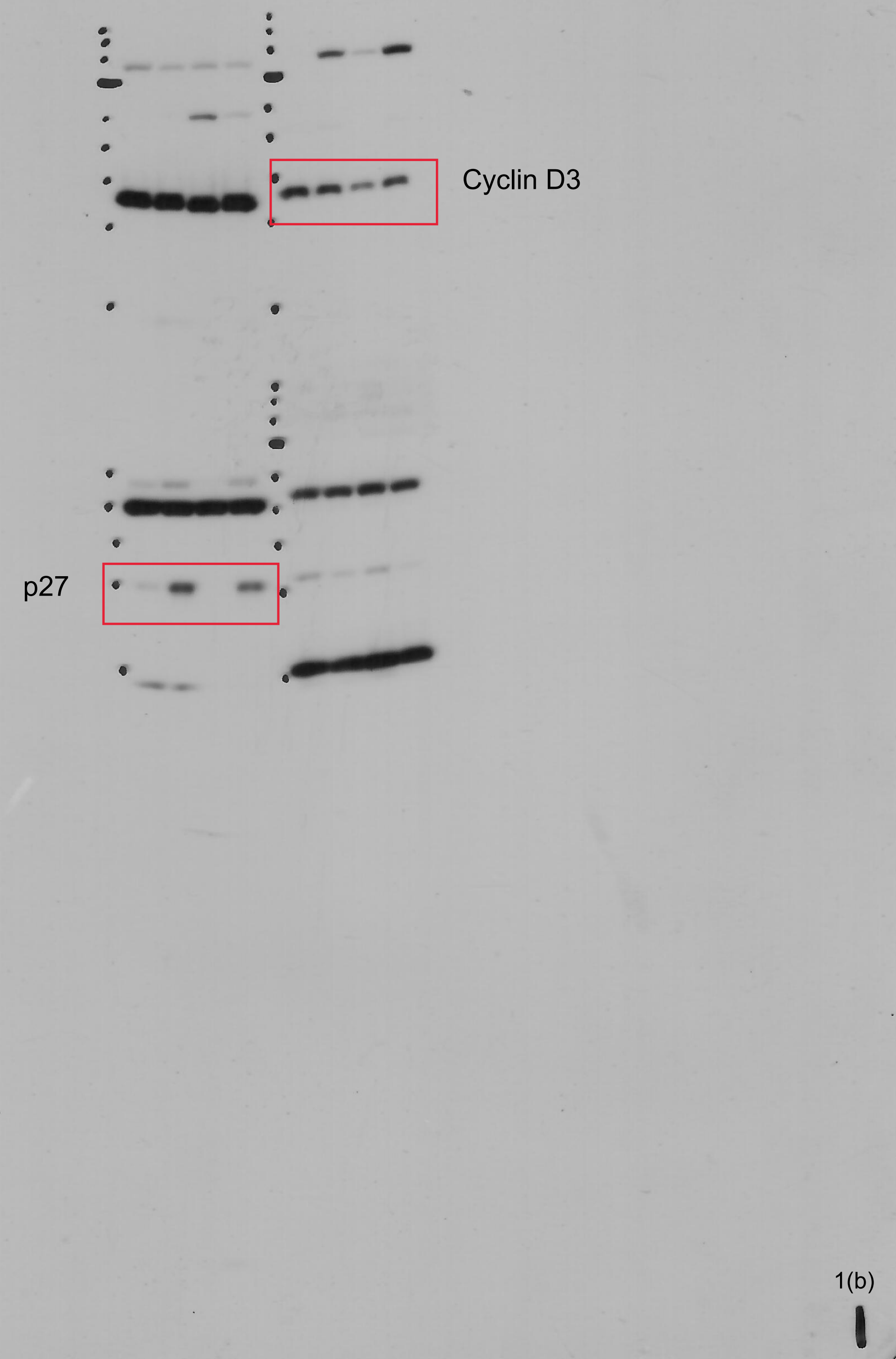

Fig. 1B. HeLa cells transfected with EZH2 or CNTL (siLuc) siRNAs for 48 h, in the presence or absence of nocodazole.
Exposure #2(b): proteins used in the manuscript from this exposure: SUZ12, Cyclin B1.
Samples are ordered in the same way as blot #1(a).

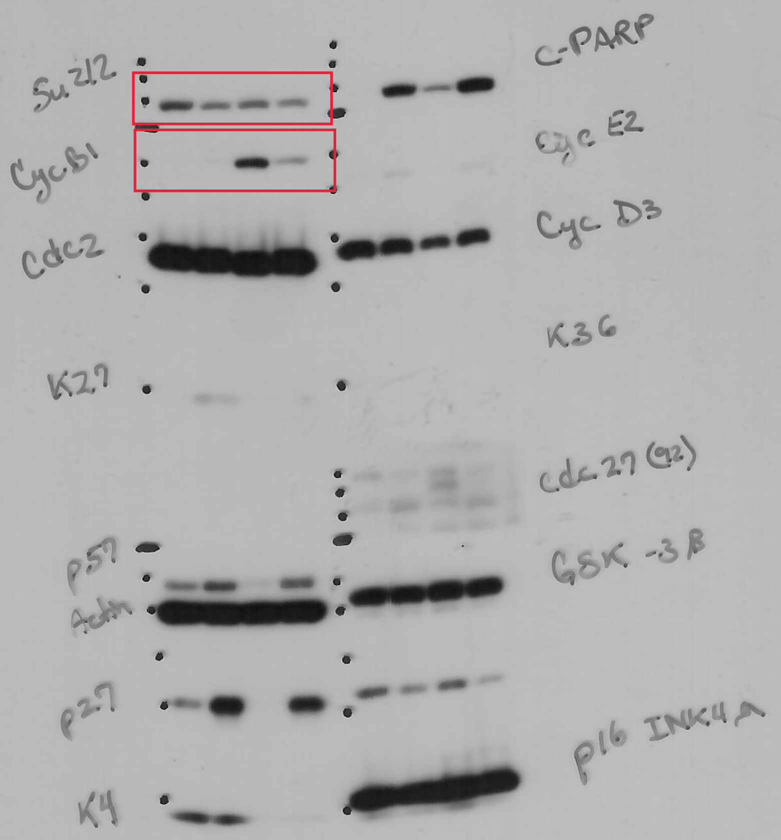

2(b)

2

Fig. 1B. HeLa cells transfected with EZH2 or CNTL (siLuc) siRNAs for 48 h, in the presence or absence of nocodazole.
Exposure #4(b): proteins used in the manuscript from this exposure: Cyclin E2.
Samples are ordered in the same way as blot #1(a).

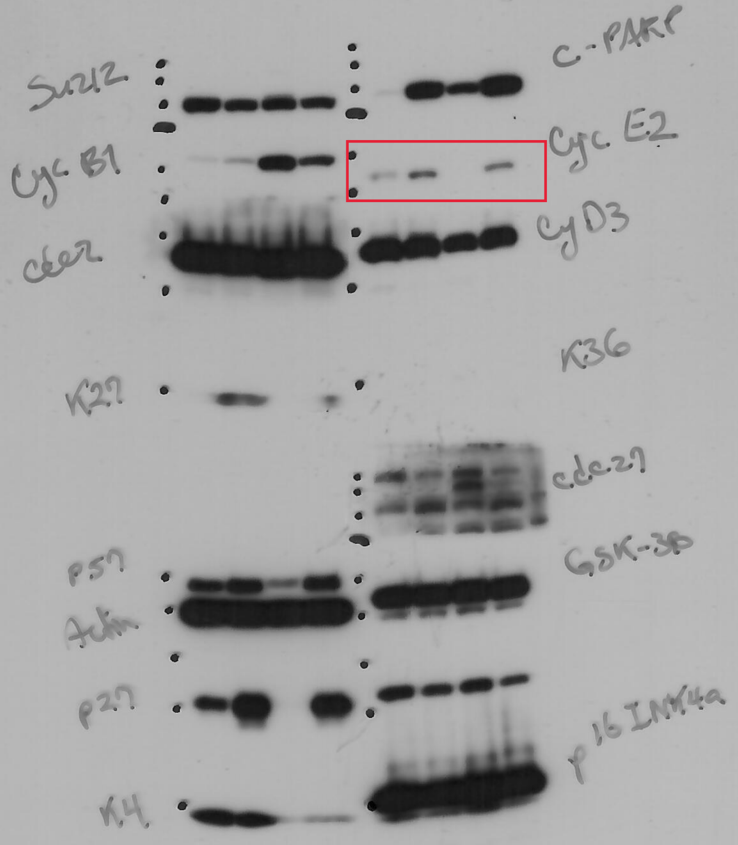

4(b)

4

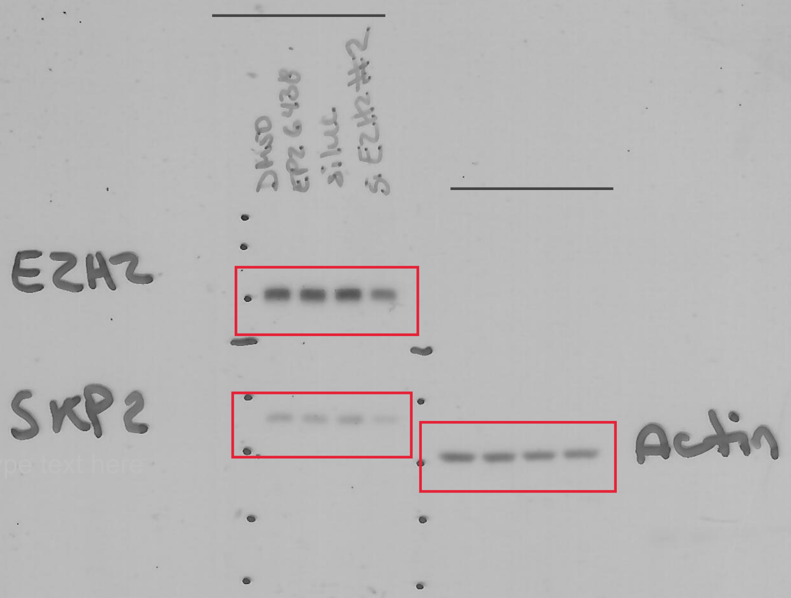

Note: The loading order is the same for all Fig. 1E exposures.

Fig. 1E. HeLa cells were treated with EPZ-6438 or DMSO or transfected with siEZH2 #2 or siLUC for 48 h.
Exposure #1: proteins used in the manuscript from this exposure: EZH2, SKP2, Actin.

12.21.23

1

Fig. 1E. HeLa cells were treated with EPZ-6438 or DMSO or transfected with siEZH2 #2 or siLUC for 48 h.
Exposure #6: proteins used in the manuscript from this exposure: p57, CDK1.

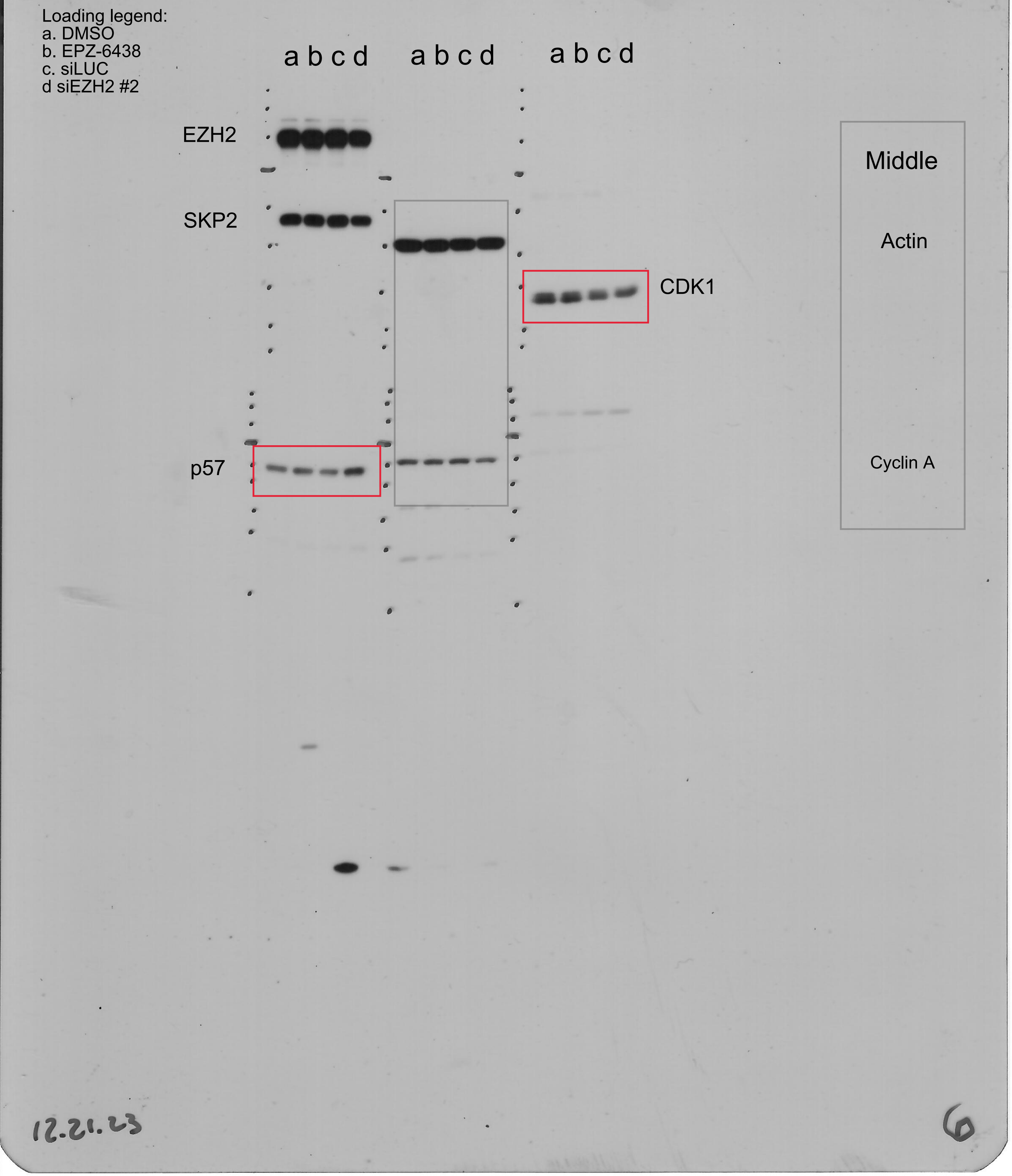

Fig. 1E. HeLa cells were treated with EPZ-6438 or DMSO or transfected with siEZH2 #2 or siLUC for 48 h.
Exposure #8: proteins used in the manuscript from this exposure: p27, c-MYC.

Loading legend:
a. DMSO
b. EPZ-6438
c. siLUC
d siEZH2 #2

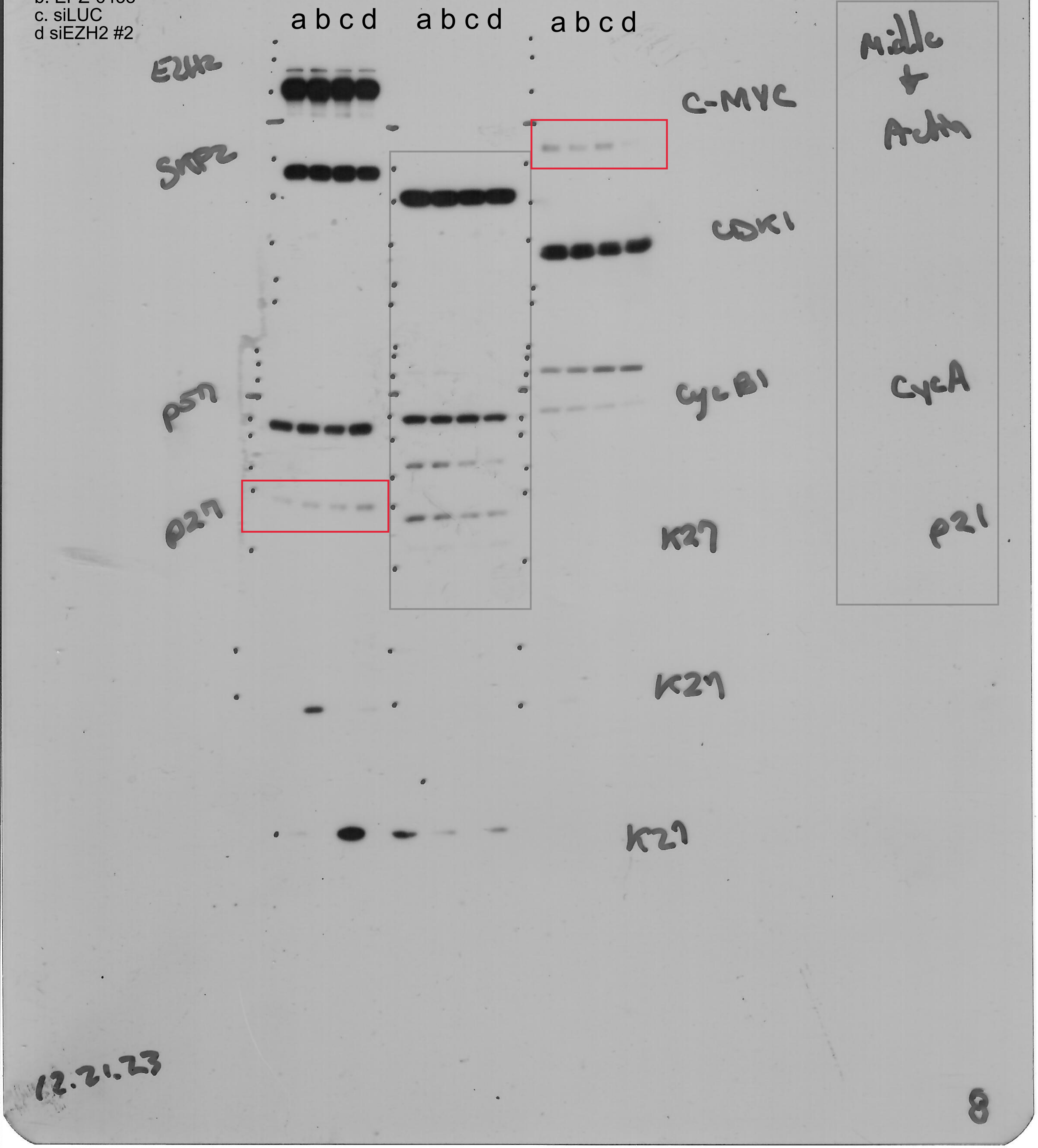

Fig. 1E. HeLa cells were treated with EPZ-6438 or DMSO or transfected with siEZH2 #2 or siLUC for 48 h.

Exposure #9: proteins used in the manuscript from this exposure: p21.

Loading legend:
a. DMSO
b. EPZ-6438
c. siLUC
d siEZH2 #2

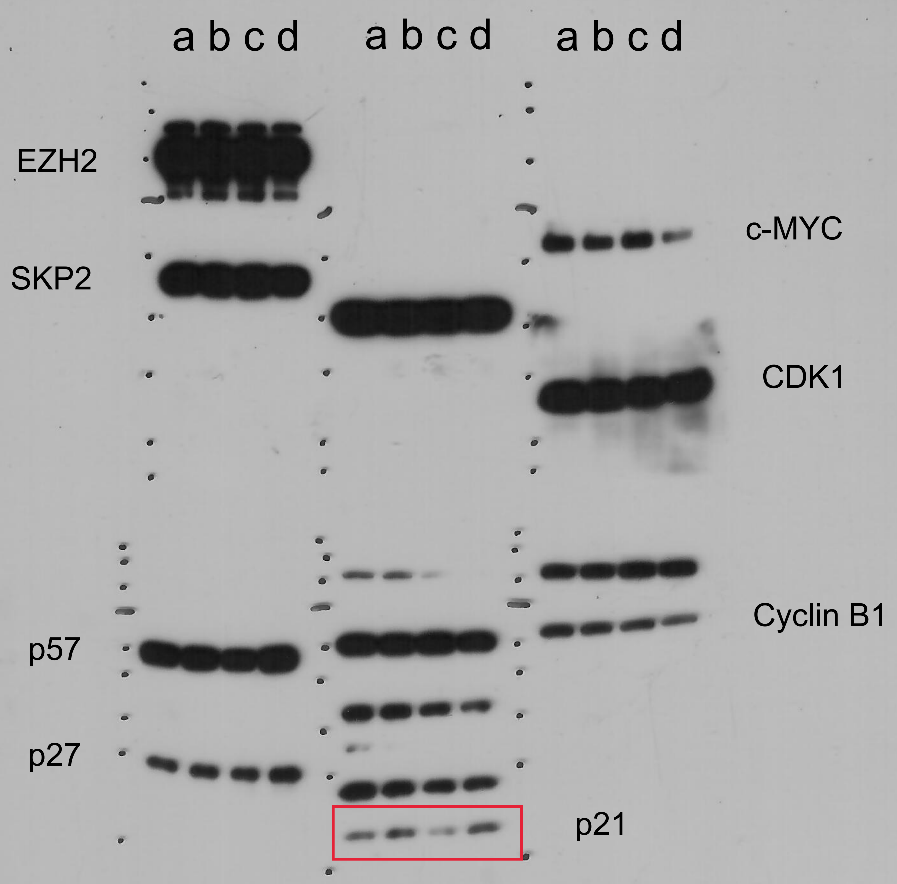

Fig. 2C H3K27me3 reblots:

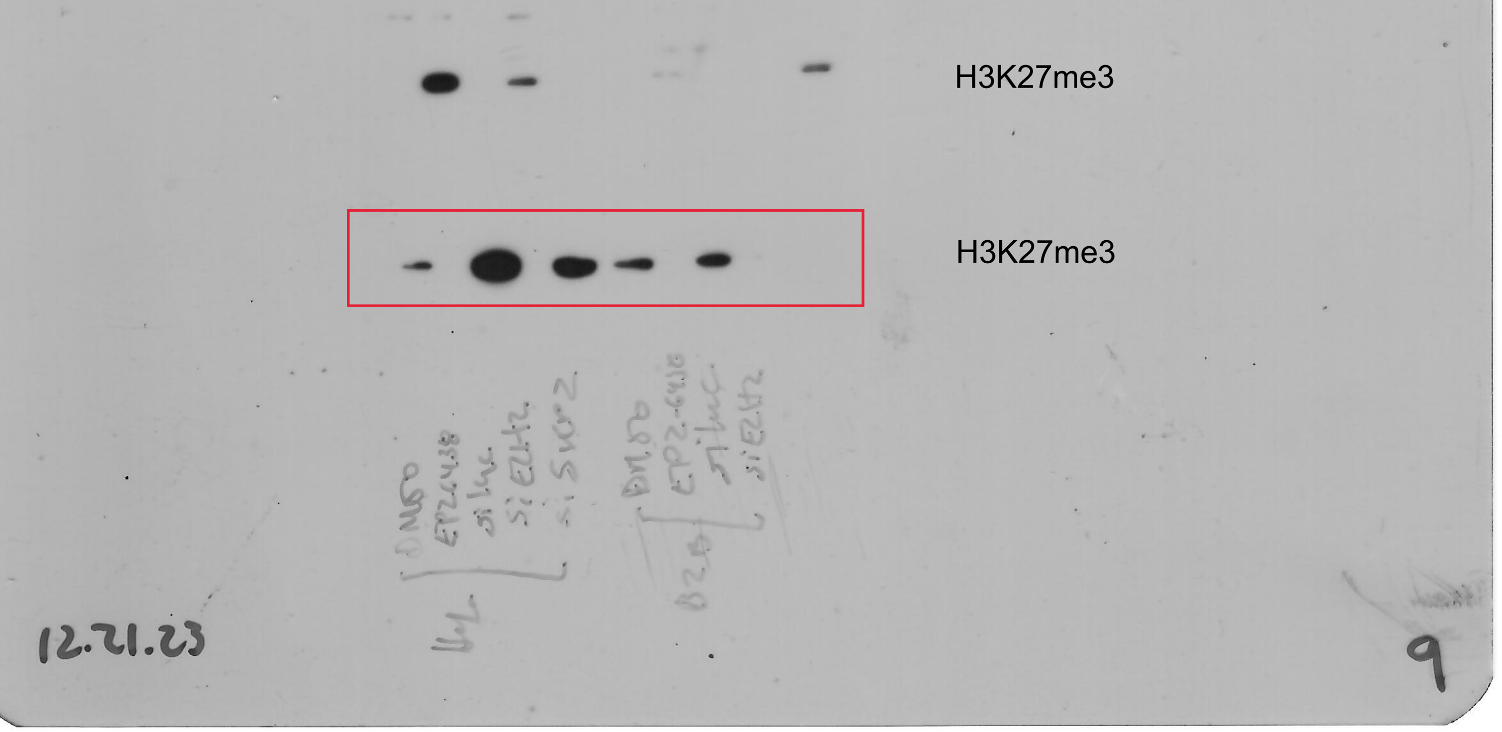

Fig. 1E. HeLa cells were treated with EPZ-6438 or DMSO or transfected with siEZH2 #2 or siLUC for 48 h.
Exposure #10: proteins used in the manuscript from this exposure: none.

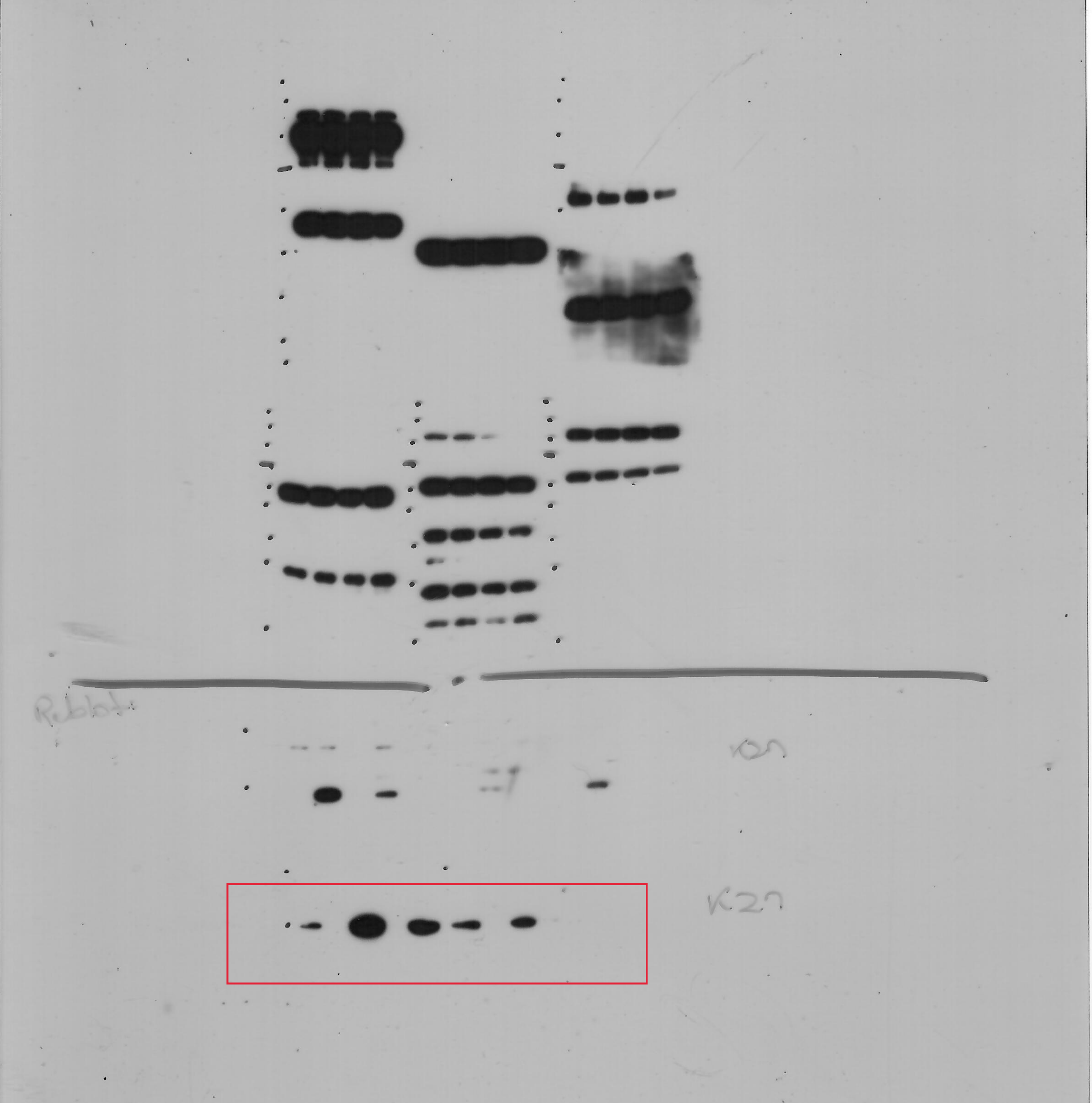

Fig. 2C H3K27me3 reblots:

12.21.23

10

Fig. 2A. HeLa and A549 cells were treated with EPZ-6438 or DMSO or transfected with siEZH2 or siLUC for 48 h.
Exposure #1 and #2: proteins used in the manuscript from these exposure: EZH2, Cyclin D3, H3K27me3.

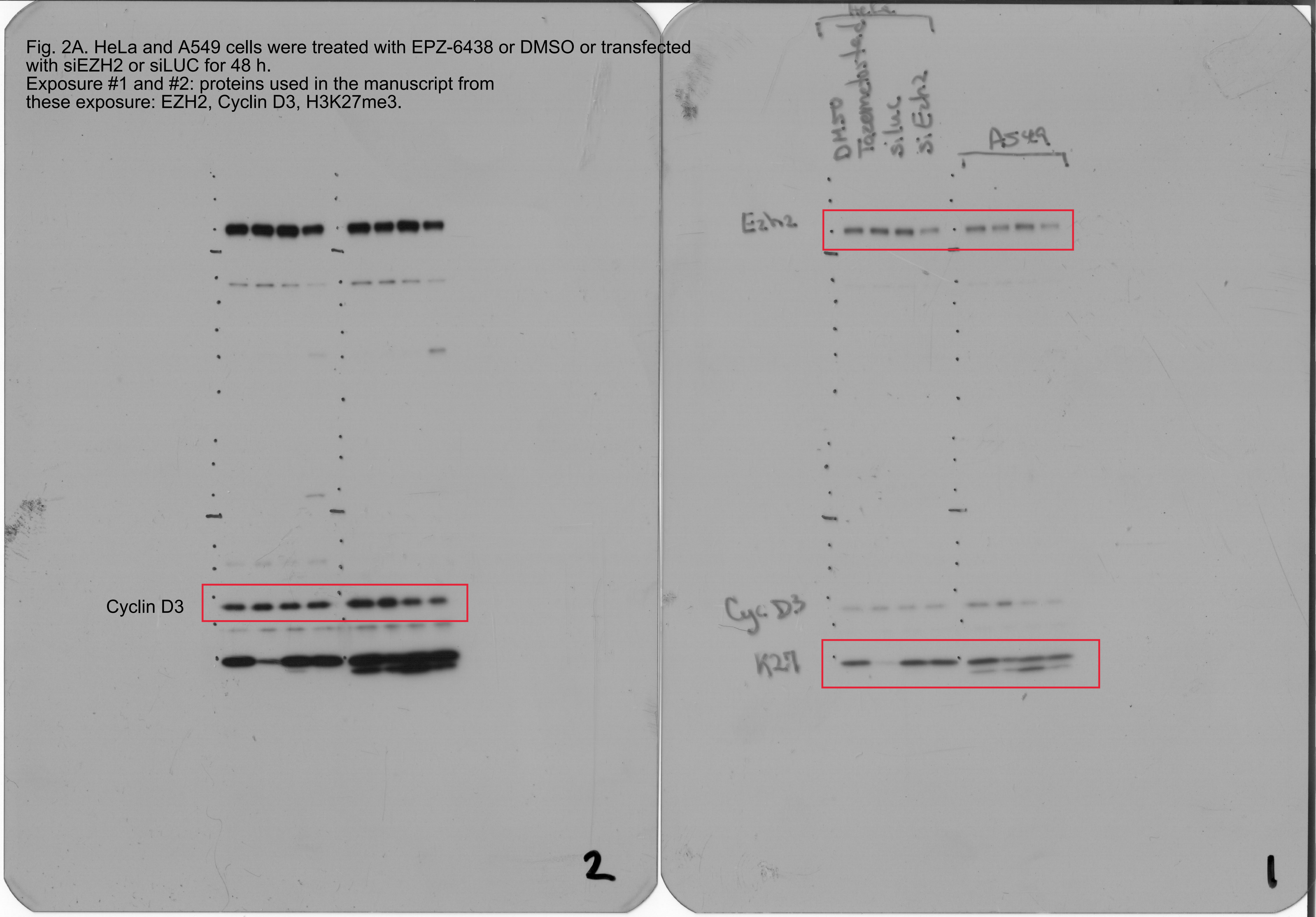

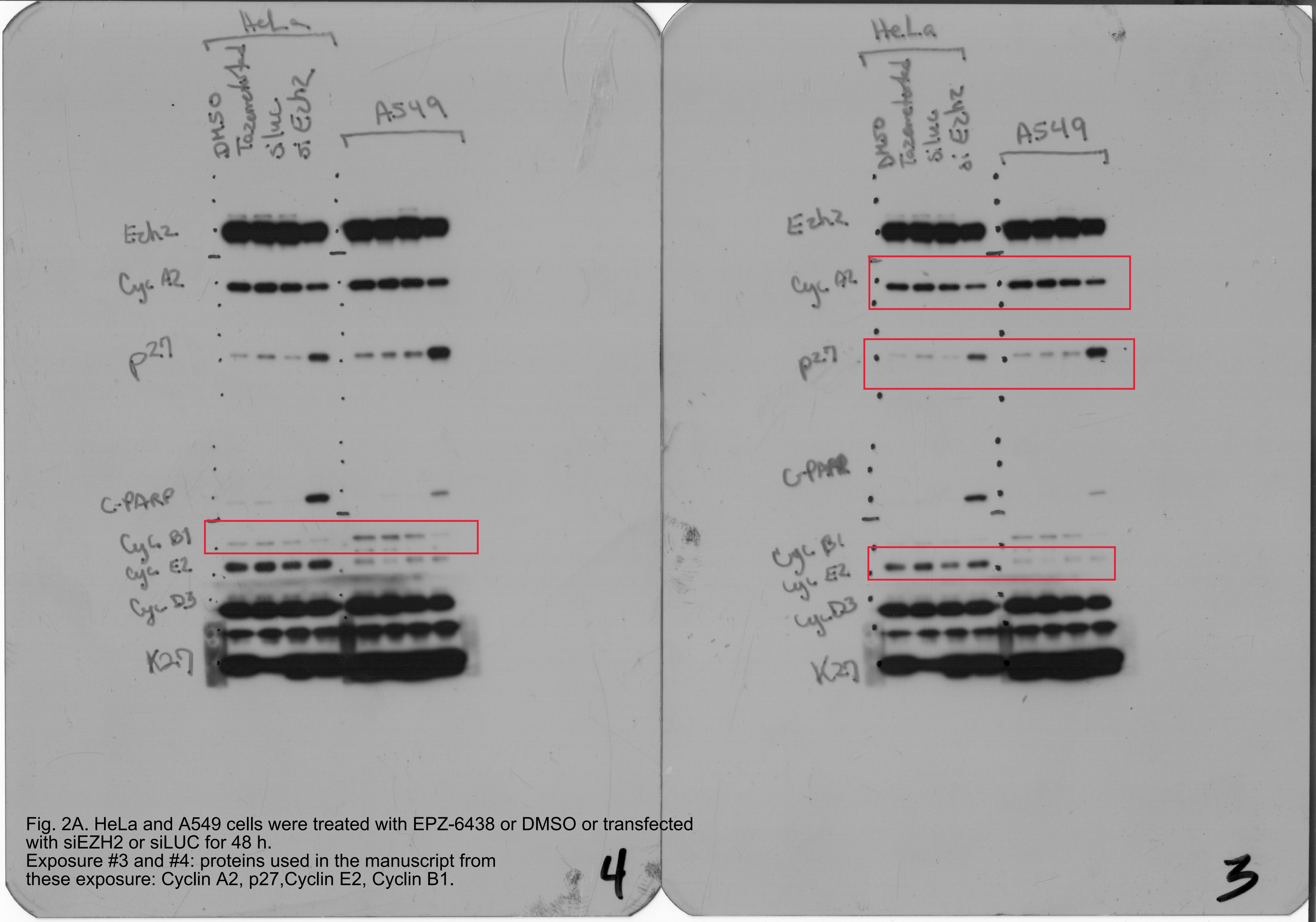

Fig. 2A. HeLa and A549 cells were treated with EPZ-6438 or DMSO or transfected with siEZH2 or siLUC for 48 h.
Exposure #3 and #4: proteins used in the manuscript from these exposure: Cyclin A2, p27,Cyclin E2, Cyclin B1.

Fig. 2B. HCT116 WT and HCT116 p53 null cells were treated with EPZ-6438 or DMSO or transfected with siEZH2 or siLUC for 48 h.
Exposure #1: proteins used in the manuscript from this exposure: Cyclin A2, H3K27me3, EZH2, Actin.

Loading legend:
a. DMSO
b. EPZ-6438 (Tazemetostat)
c. siLUC
d siEZH2

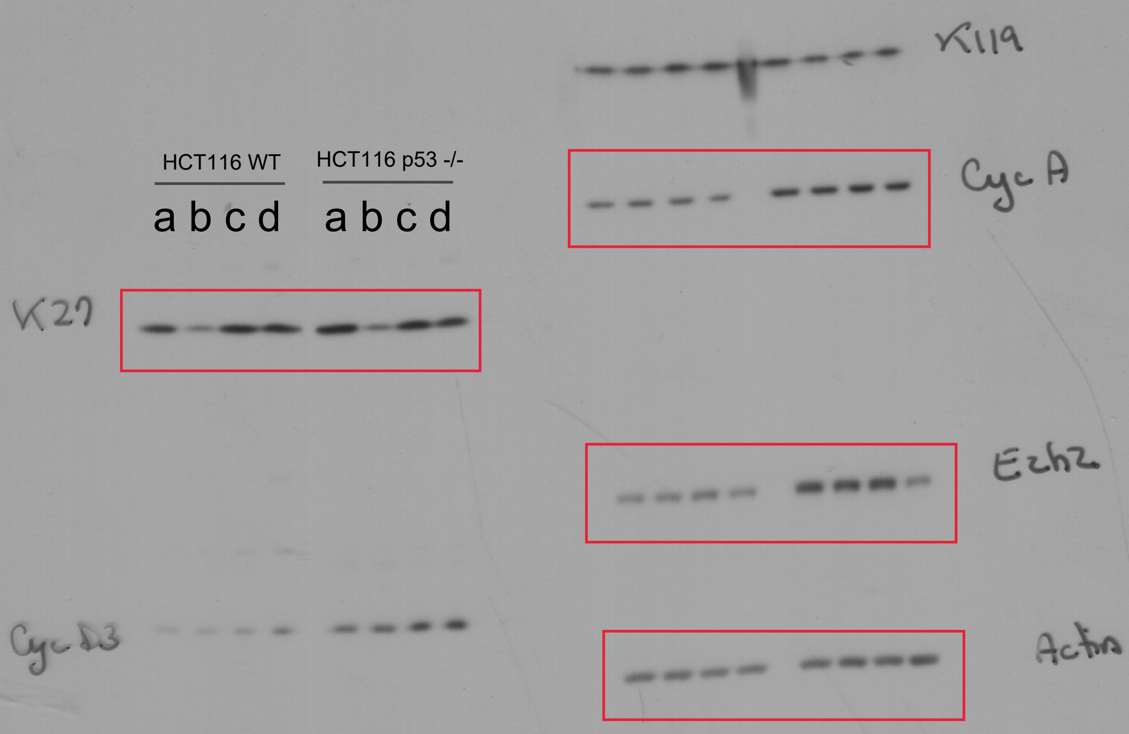

Fig. 2B. HCT116 WT and HCT116 p53 null cells were treated with EPZ-6438 or DMSO or transfected with siEZH2 or siLUC for 48 h.
Exposure #2: proteins used in the manuscript from this exposure: Cyclin D3.

Loading legend:
a. DMSO
b. EPZ-6438 (Tazemetostat)
c. siLUC
d siEZH2

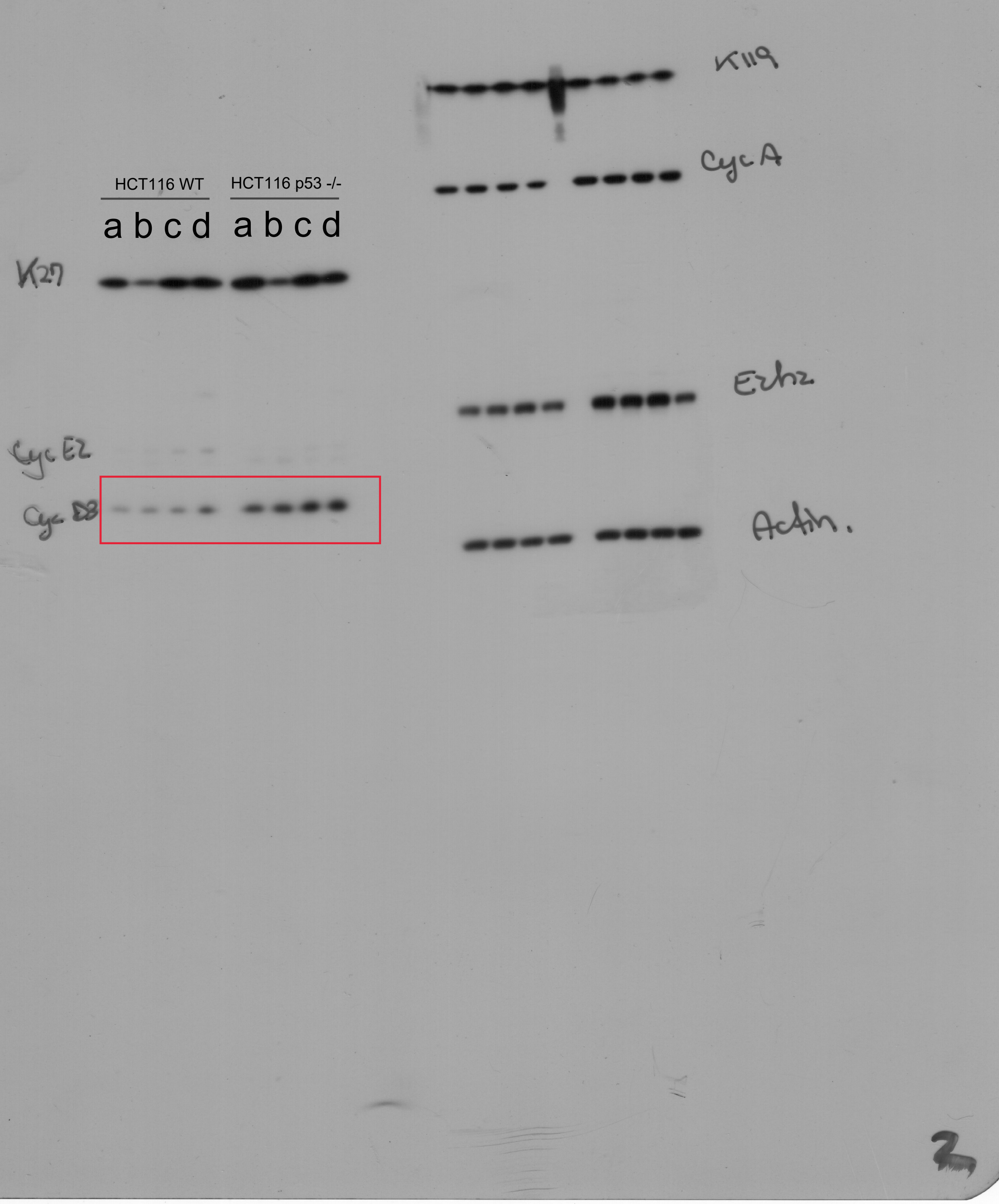

Fig. 2B. HCT116 WT and HCT116 p53 null cells were treated with EPZ-6438 or DMSO or transfected with siEZH2 or siLUC for 48 h.
Exposure #5: proteins used in the manuscript from this exposure: p27, Cyclin E2.

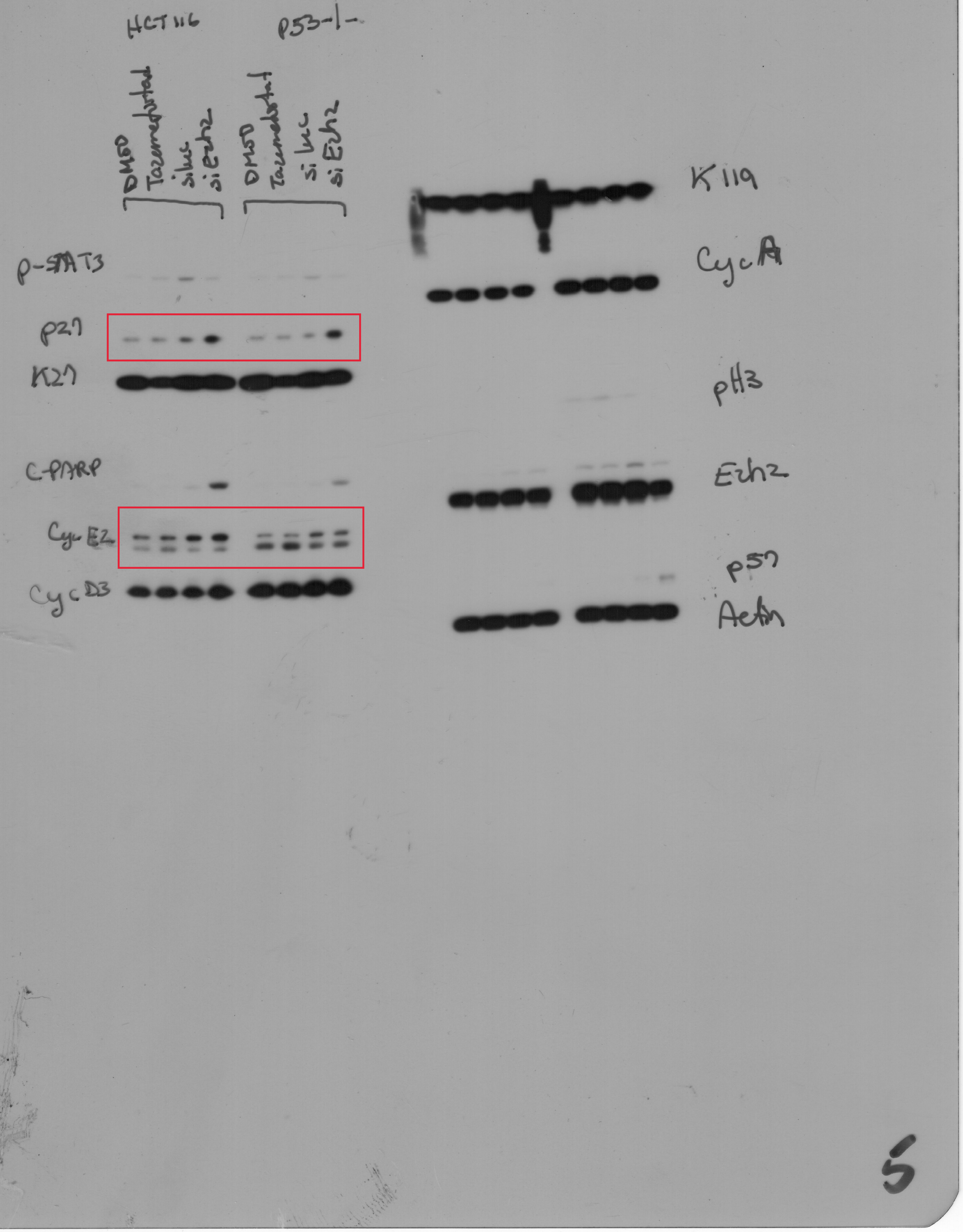

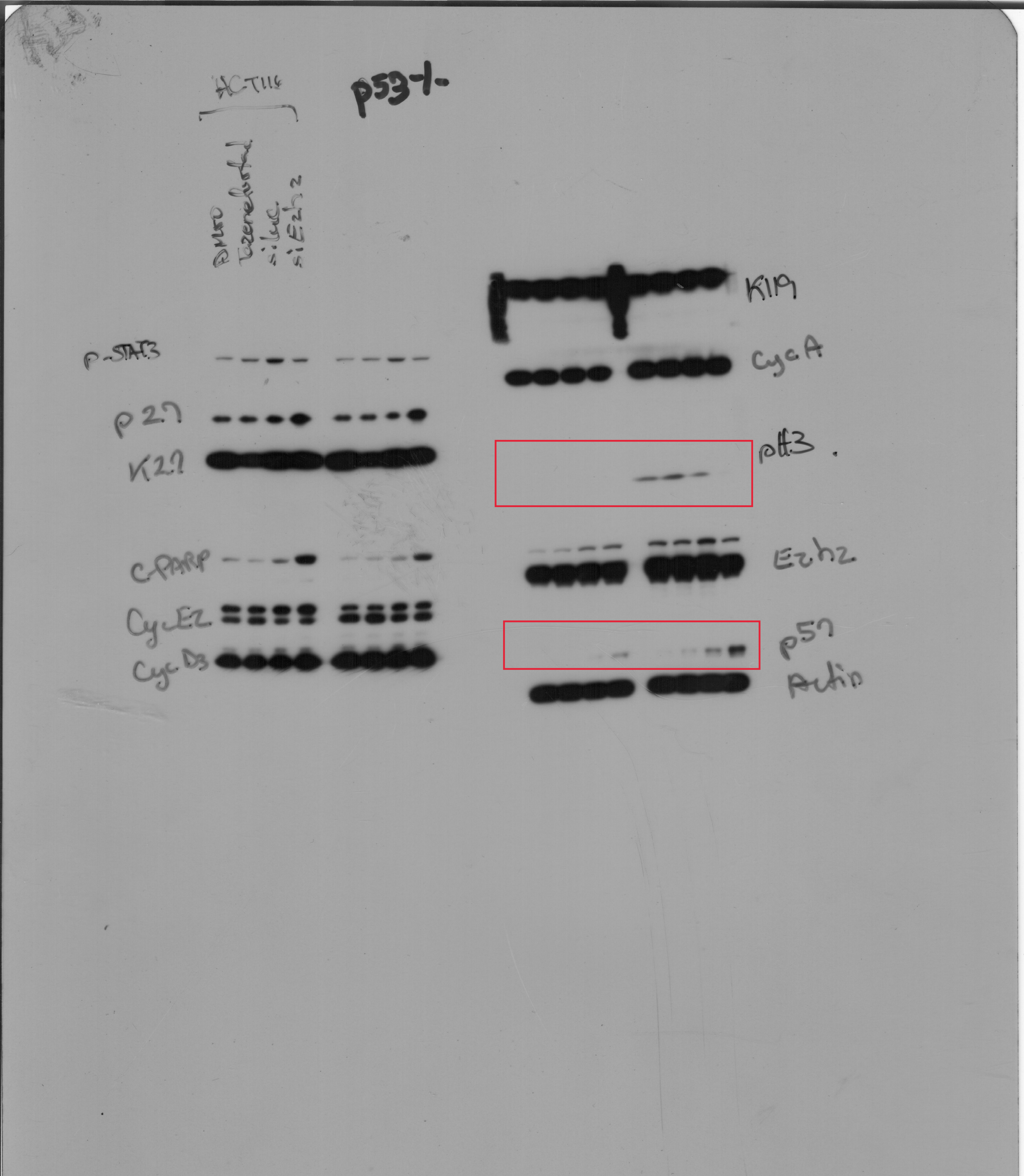

Fig. 2B. HCT116 WT and HCT116 p53 null cells were treated with EPZ-6438 or DMSO or transfected with siEZH2 or siLUC for 48 h.
Exposure #6: proteins used in the manuscript from this exposure: p-H3, p57.

6

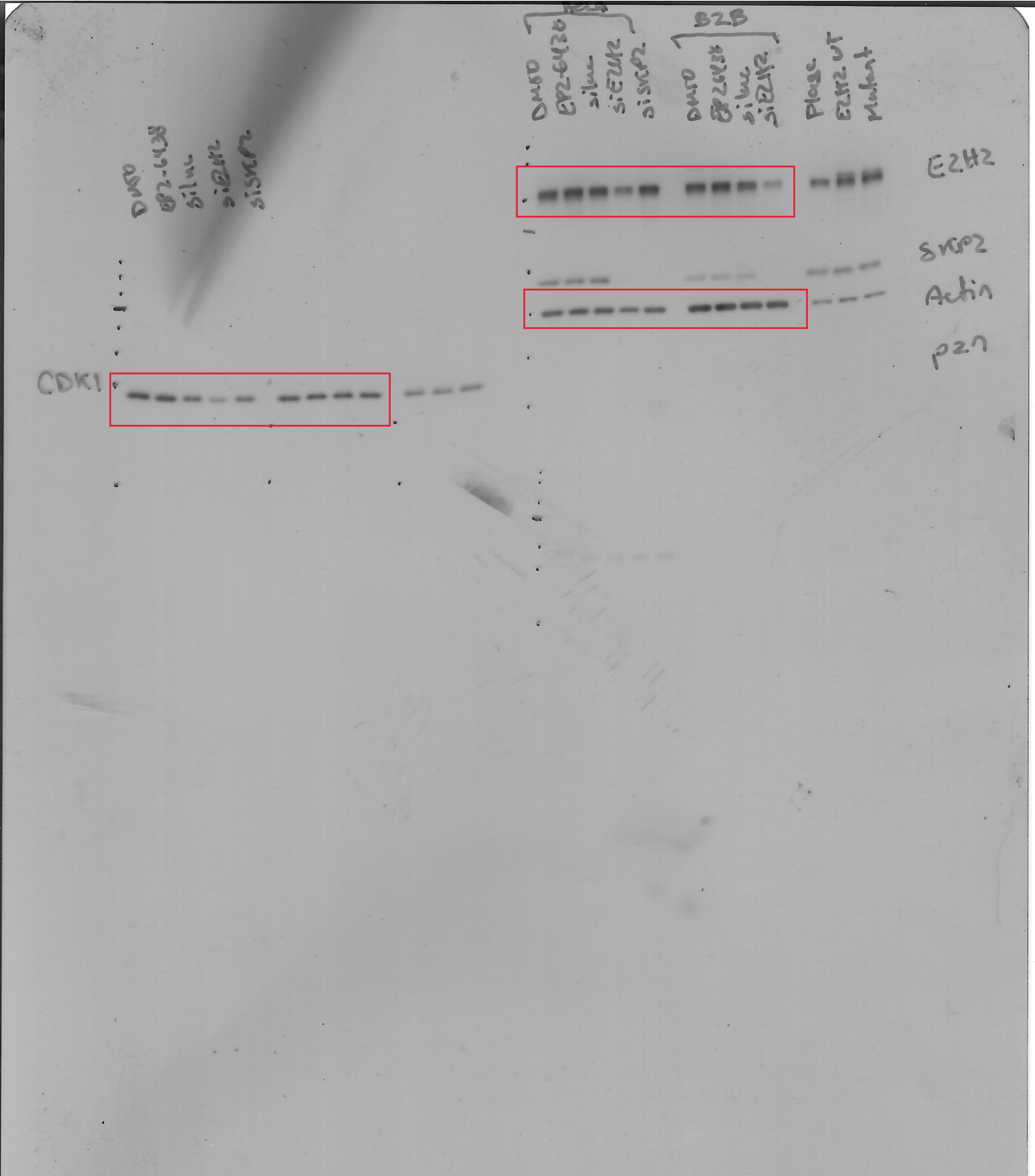

Fig. 2C. HeLa and BEAS-2B cells were treated with EPZ-6438 or DMSO or transfected with siEZH2, siSKP2 or siLUC for 48 h.
Exposure #1: proteins used in the manuscript from this exposure: EZH2, CDK1,Actin.

Fig. 2C. HeLa and BEAS-2B cells were treated with EPZ-6438 or DMSO or transfected with siEZH2, siSKP2 or siLUC for 48 h.
Exposure #2: proteins used in the manuscript from this exposure: SKP2, p57.

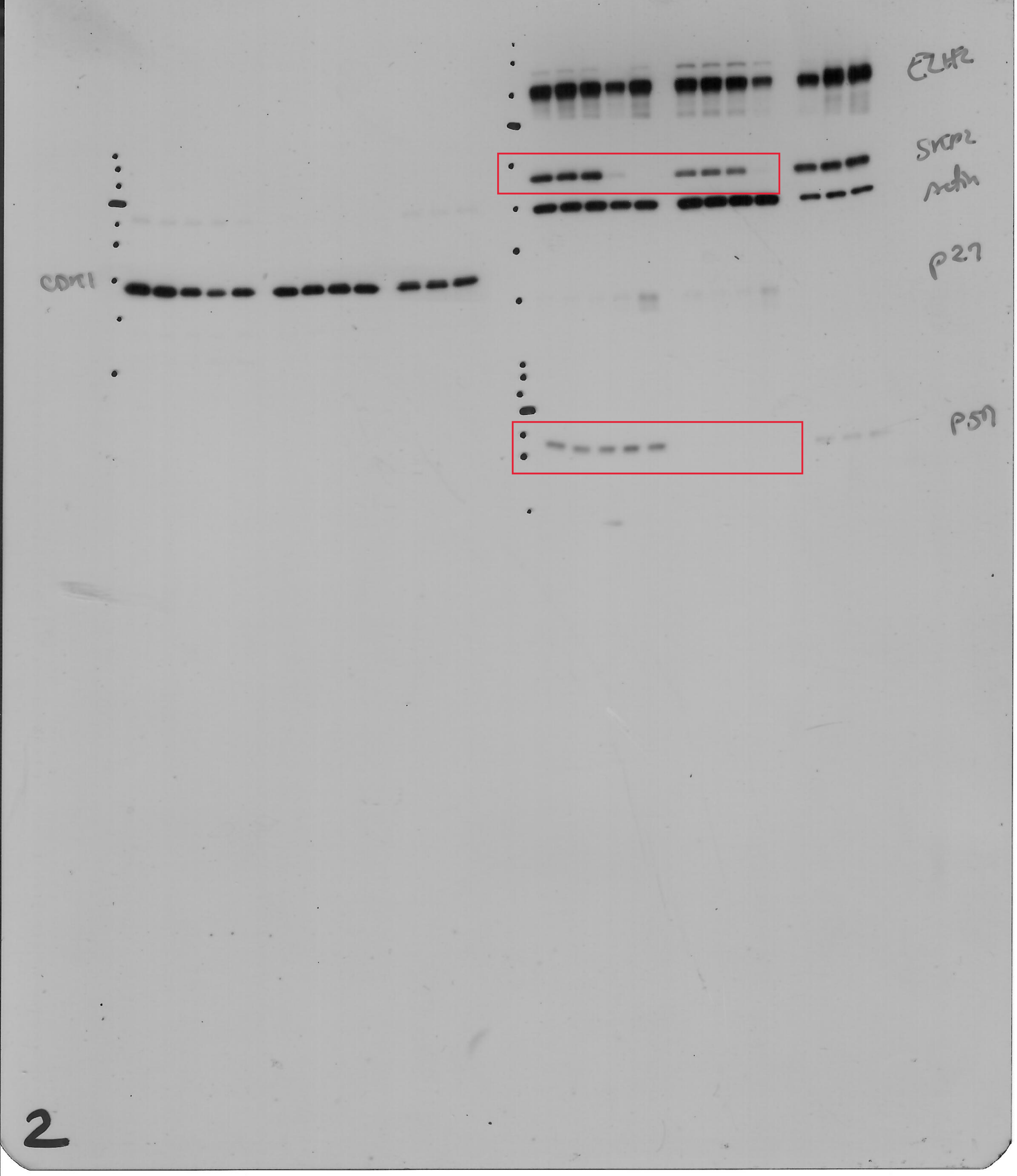

Fig. 2C. HeLa and BEAS-2B cells were treated with EPZ-6438 or DMSO or transfected with siEZH2, siSKP2 or siLUC for 48 h.
Exposure #4: proteins used in the manuscript from this exposure: p27, c-MYC.

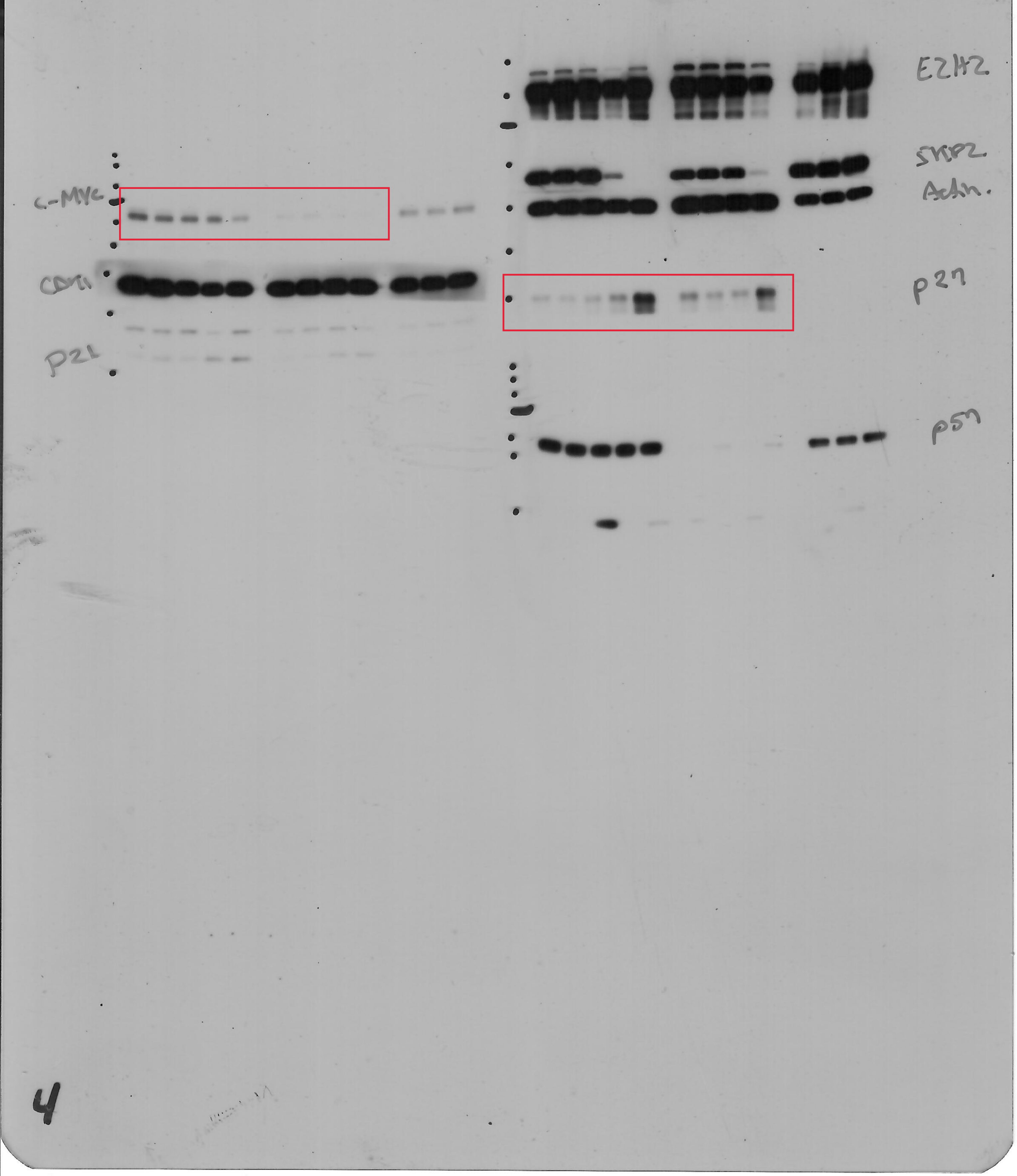

Fig. 2C. HeLa and BEAS-2B cells were treated with EPZ-6438 or DMSO or transfected with siEZH2, siSKP2 or siLUC for 48 h.
Exposure #6: proteins used in the manuscript from this exposure: p21, p57.

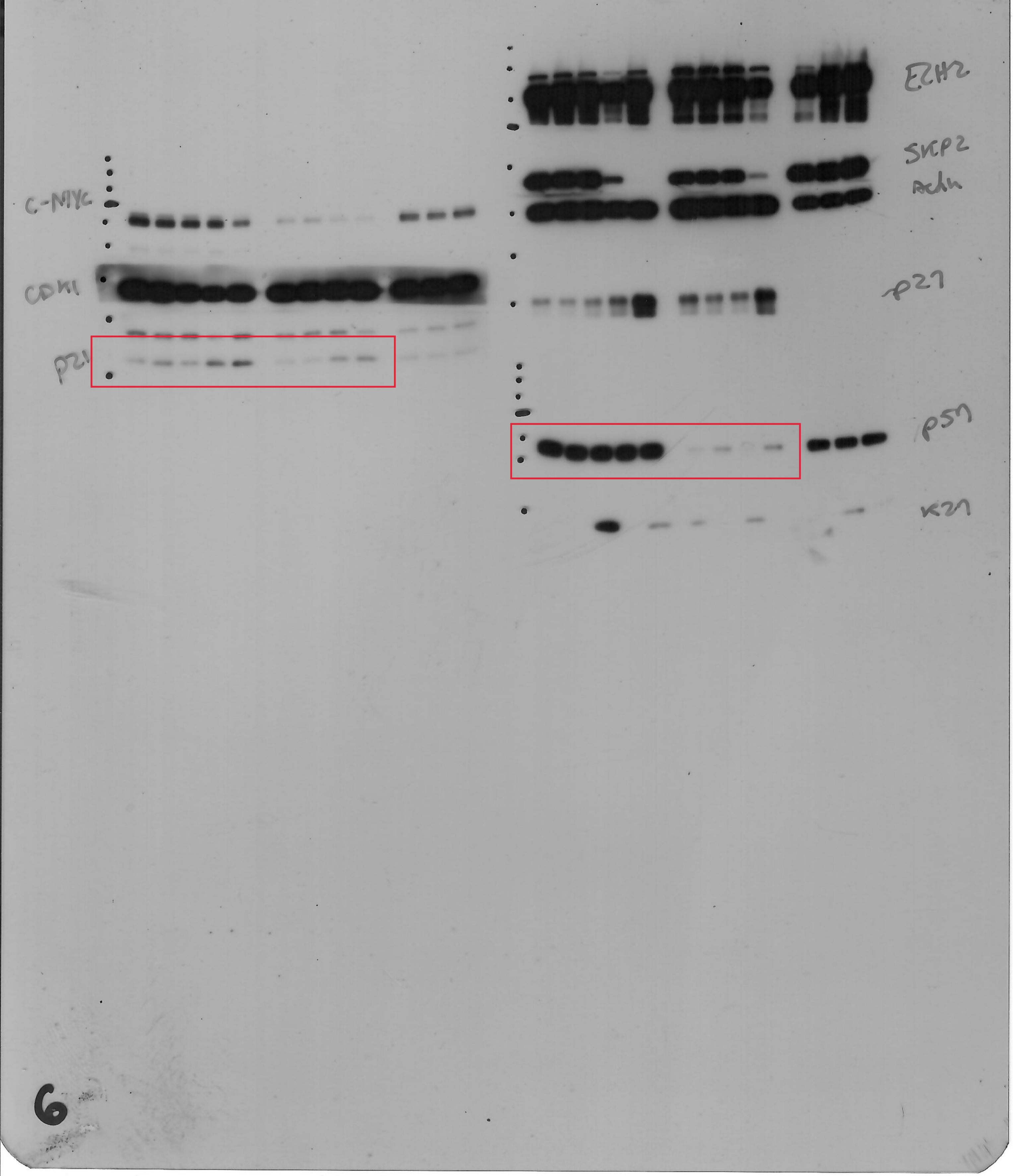

Fig. 3A. HeLa cells were treated with EPZ-6438 or DMSO or transfected with siEZH2 or siLUC for 48 h, or synchronized with the thymidine block or by nocodazole treatment for 18 h.
Exposure #1: proteins used in the manuscript from this exposure: EZH2, SKP2, H3K27me3.

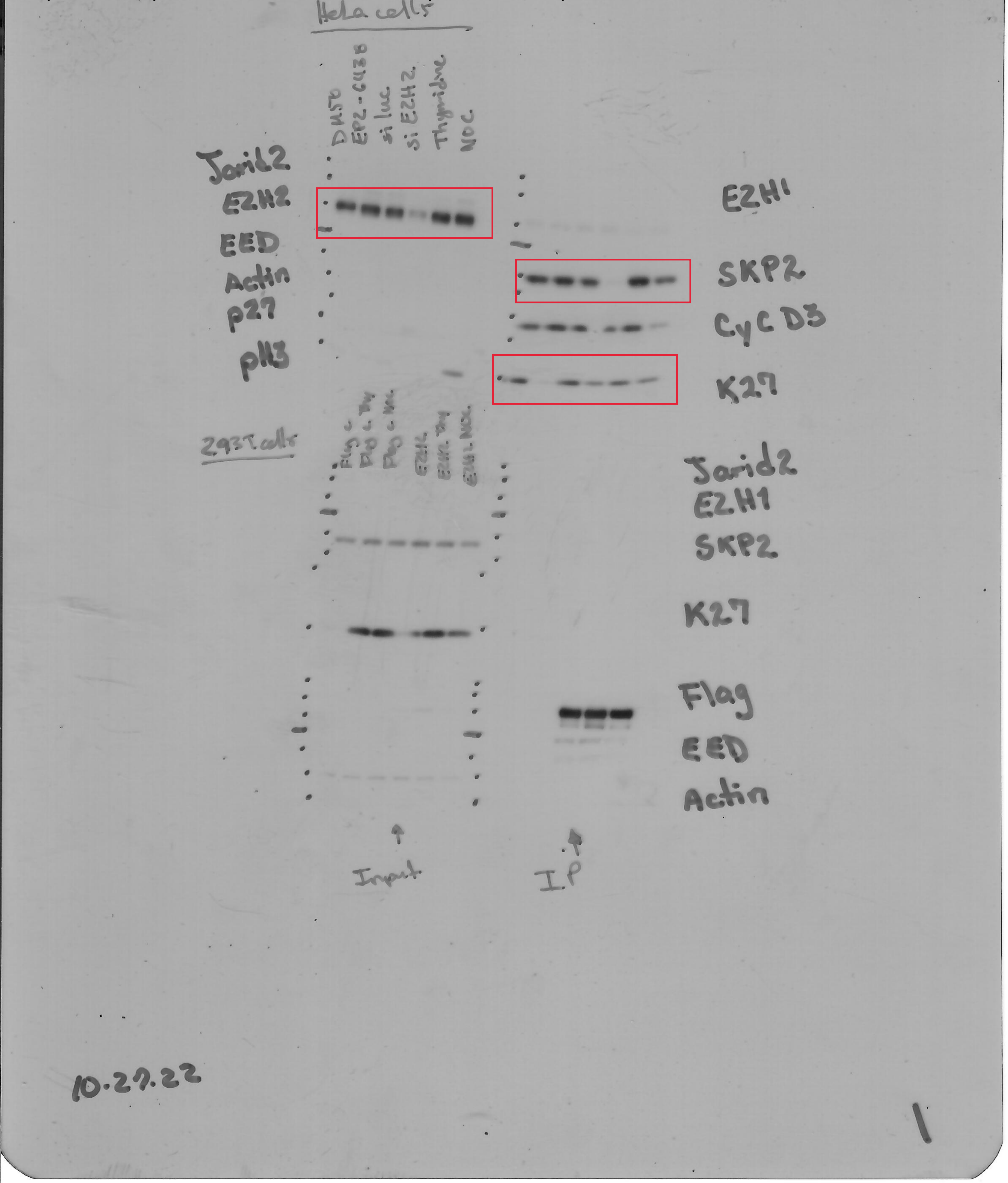

Fig. 3A. HeLa cells were treated with EPZ-6438 or DMSO or transfected with siEZH2 or siLUC for 48 h, or synchronized with the thymidine block or by nocodazole treatment for 18 h.
Exposure #2: proteins used in the manuscript from this exposure: Cyclin D3.

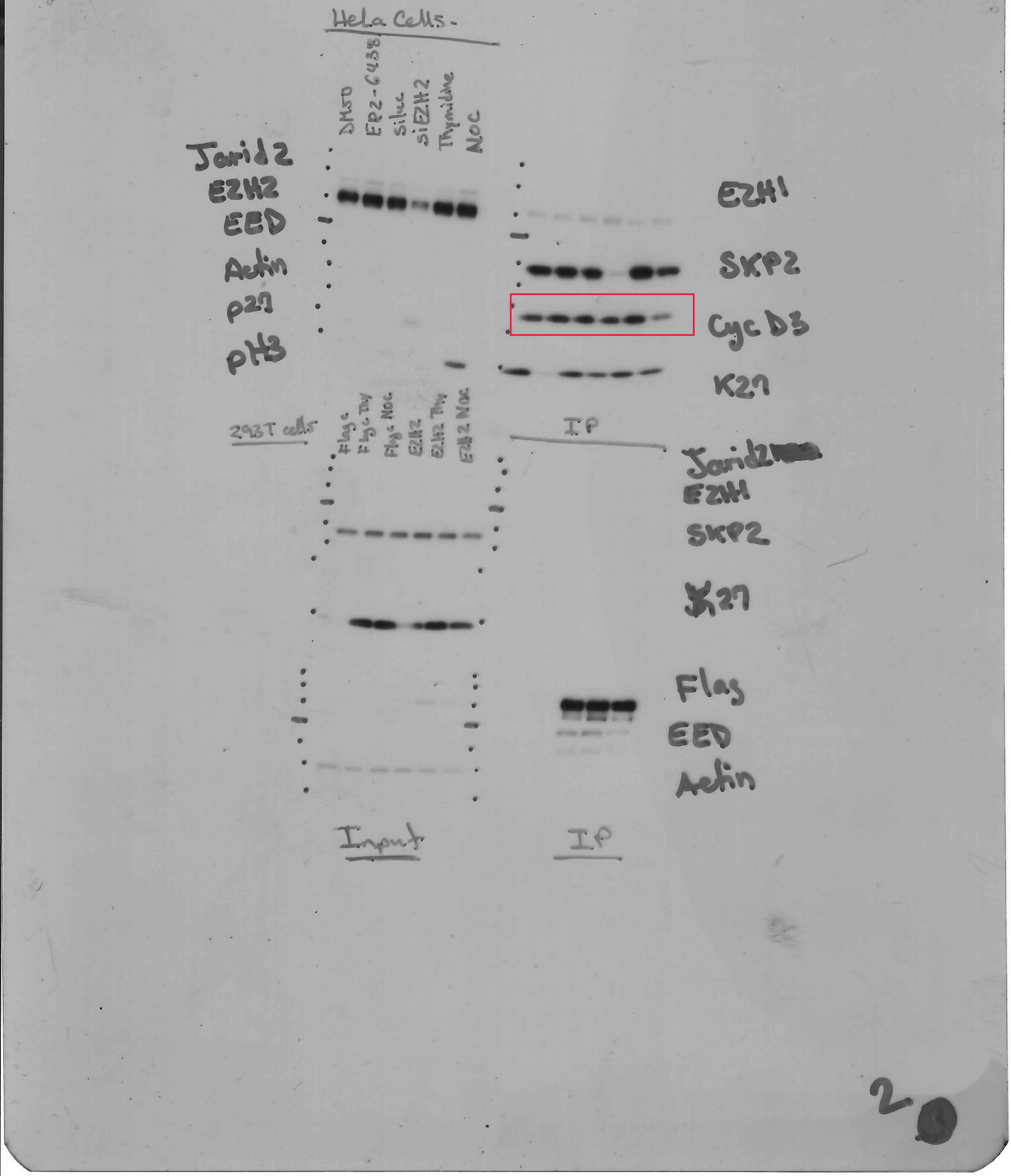

Fig. 3A. HeLa cells were treated with EPZ-6438 or DMSO or transfected with siEZH2 or siLUC for 48 h, or synchronized with the thymidine block or by nocodazole treatment for 18 h.
Exposure #3: proteins used in the manuscript from this exposure: p-H3.

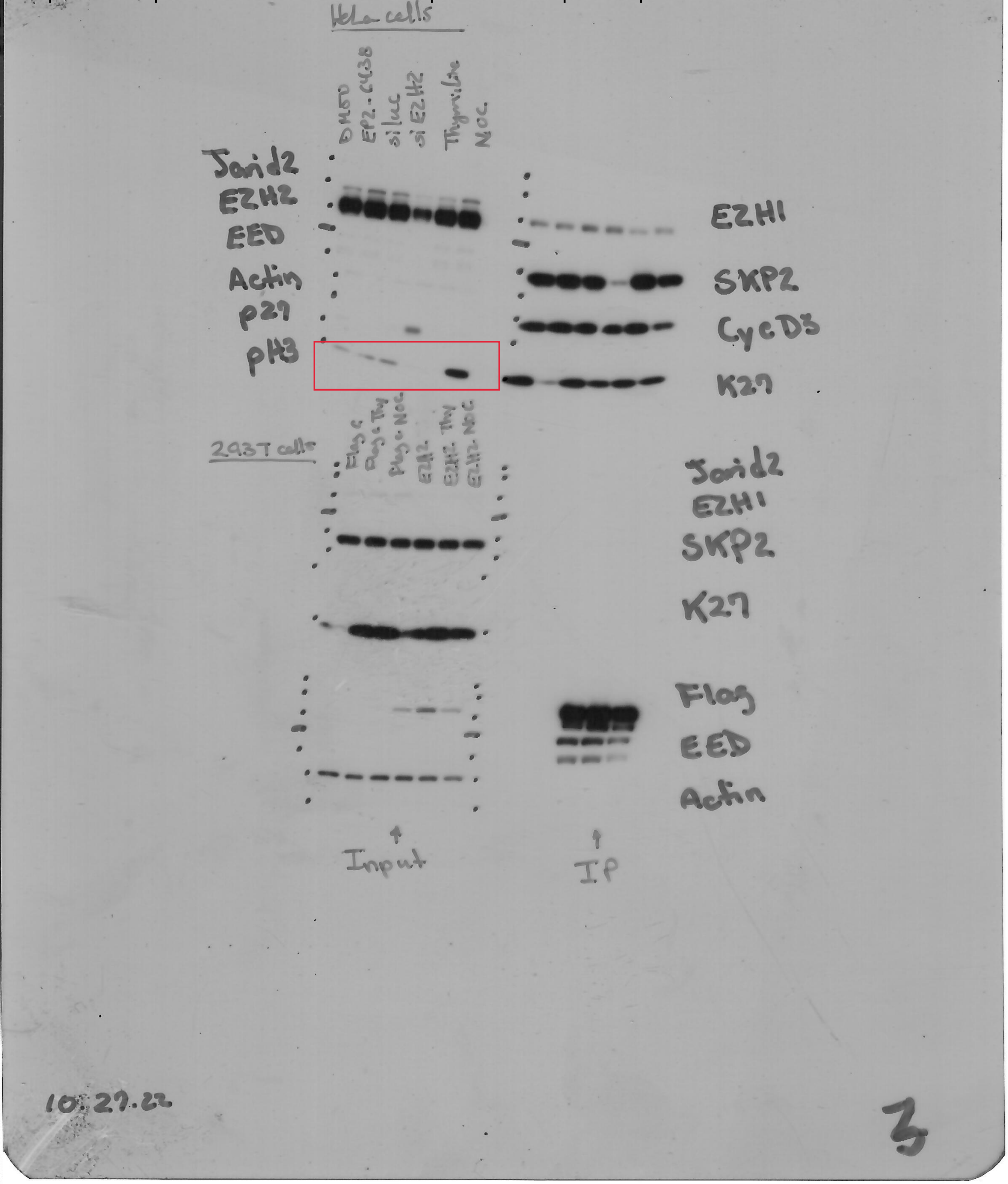

Fig. 3A. HeLa cells were treated with EPZ-6438 or DMSO or transfected with siEZH2 or siLUC for 48 h, or synchronized with the thymidine block or by nocodazole treatment for 18 h.
Exposure #4: proteins used in the manuscript from this exposure: p27.

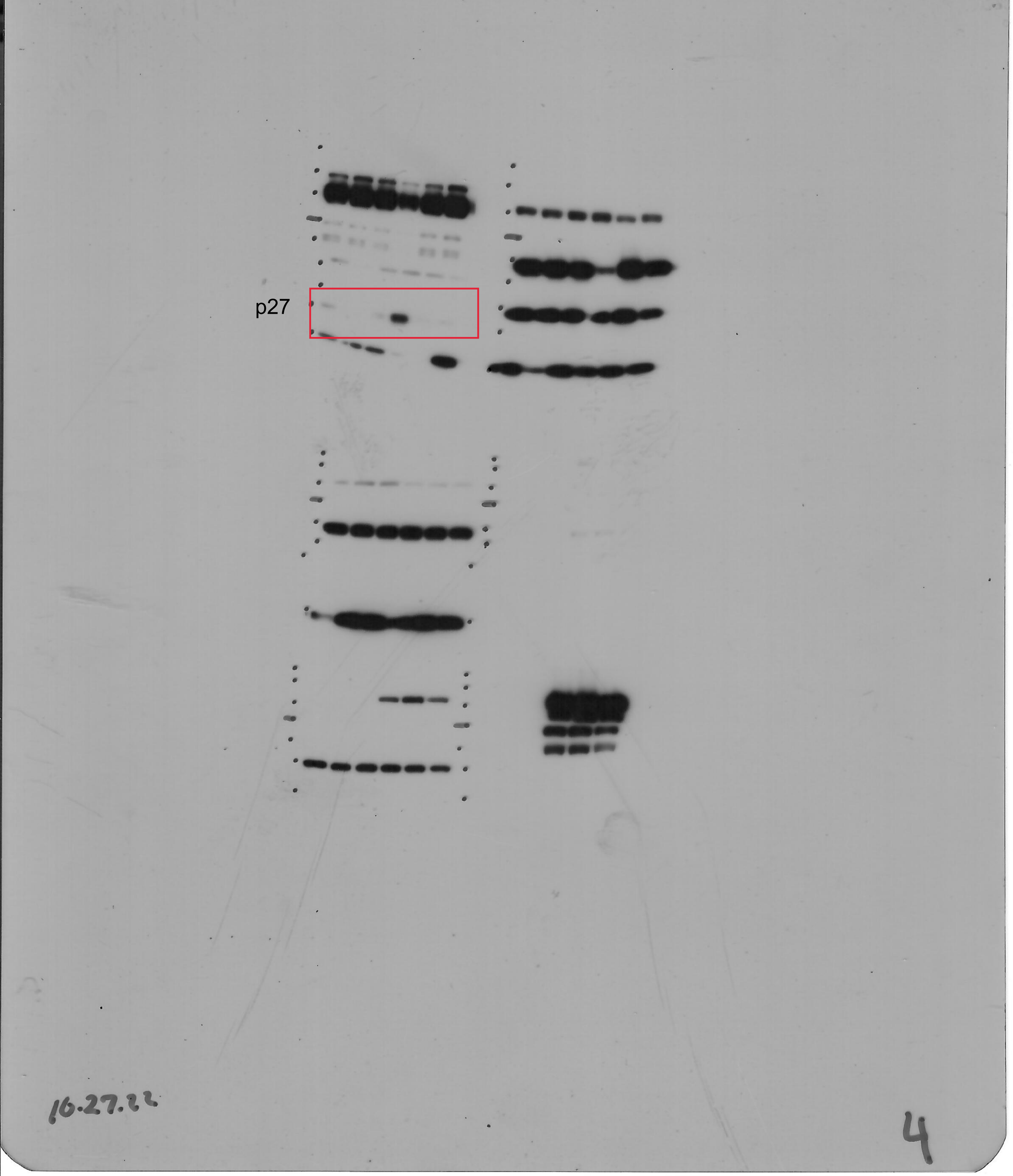

Fig. 3A. HeLa cells were treated with EPZ-6438 or DMSO or transfected with siEZH2 or siLUC for 48 h, or synchronized with the thymidine block or by nocodazole treatment for 18 h.
Exposure loading control: Actin.

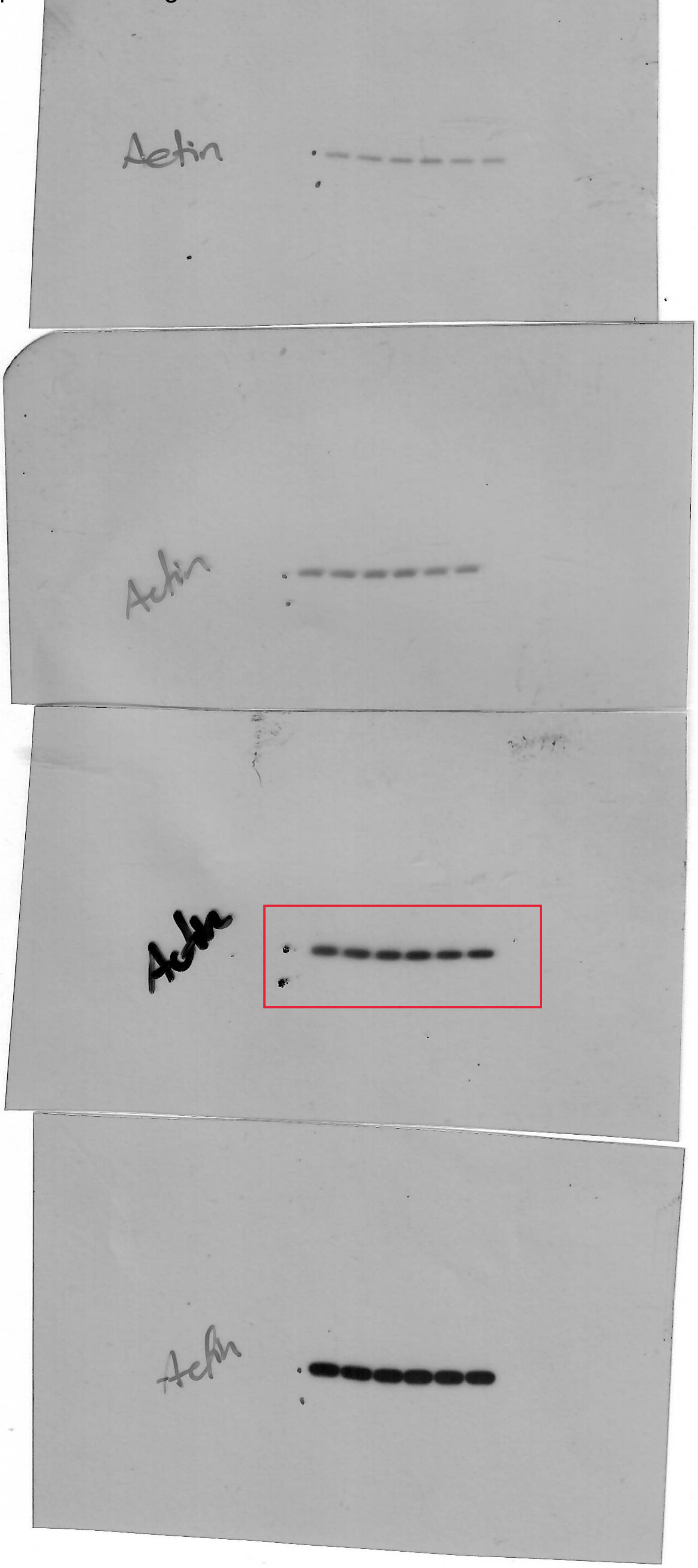

Fig. 3B. HeLa cells were transfected with siEZH1 or siEZH2 or both for 48 h.
Exposure #1: proteins used in the manuscript from this exposure: EZH2, SKP2, Cyclin D3.

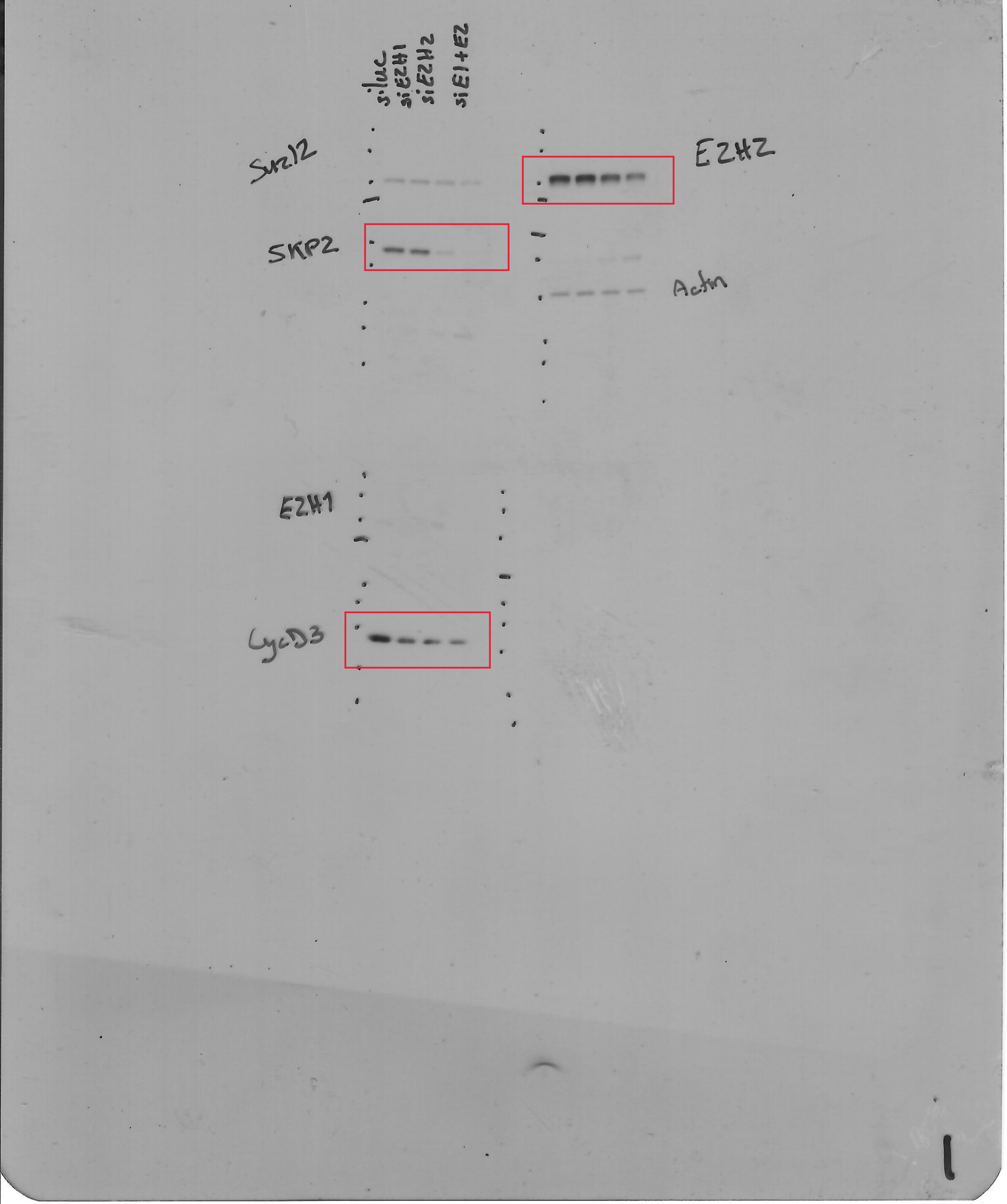

Fig. 3B. HeLa cells were transfected with siEZH1 or siEZH2 or both for 48 h.
Exposure #2: proteins used in the manuscript from this exposure: SUZ12, EZH1, p27,Actin,p57.

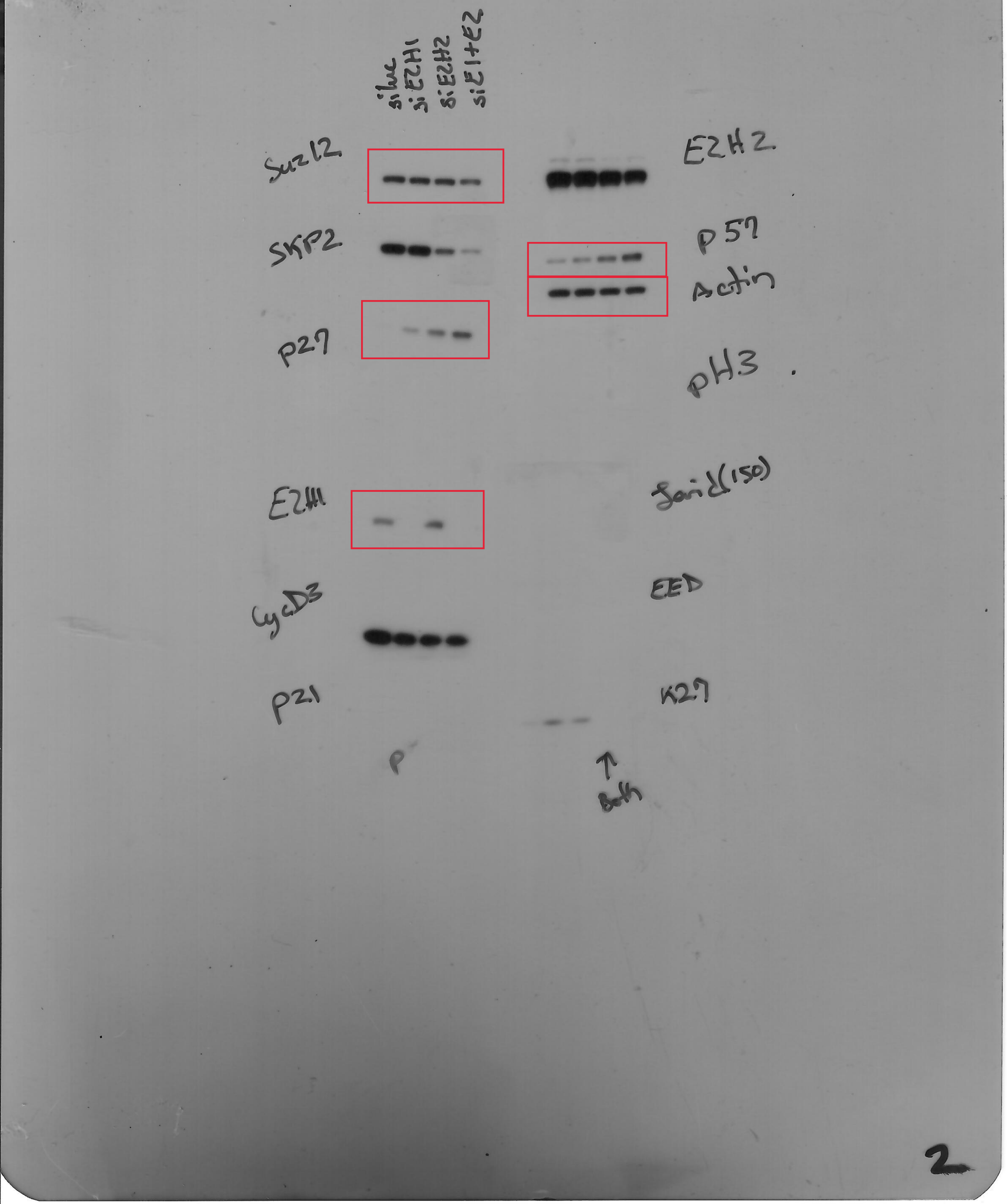

Fig. 3B. HeLa cells were transfected with siEZH1 or siEZH2 or both for 48 h.
Exposure #5: proteins used in the manuscript from this exposure: H3K27me3, p-H3, EED.

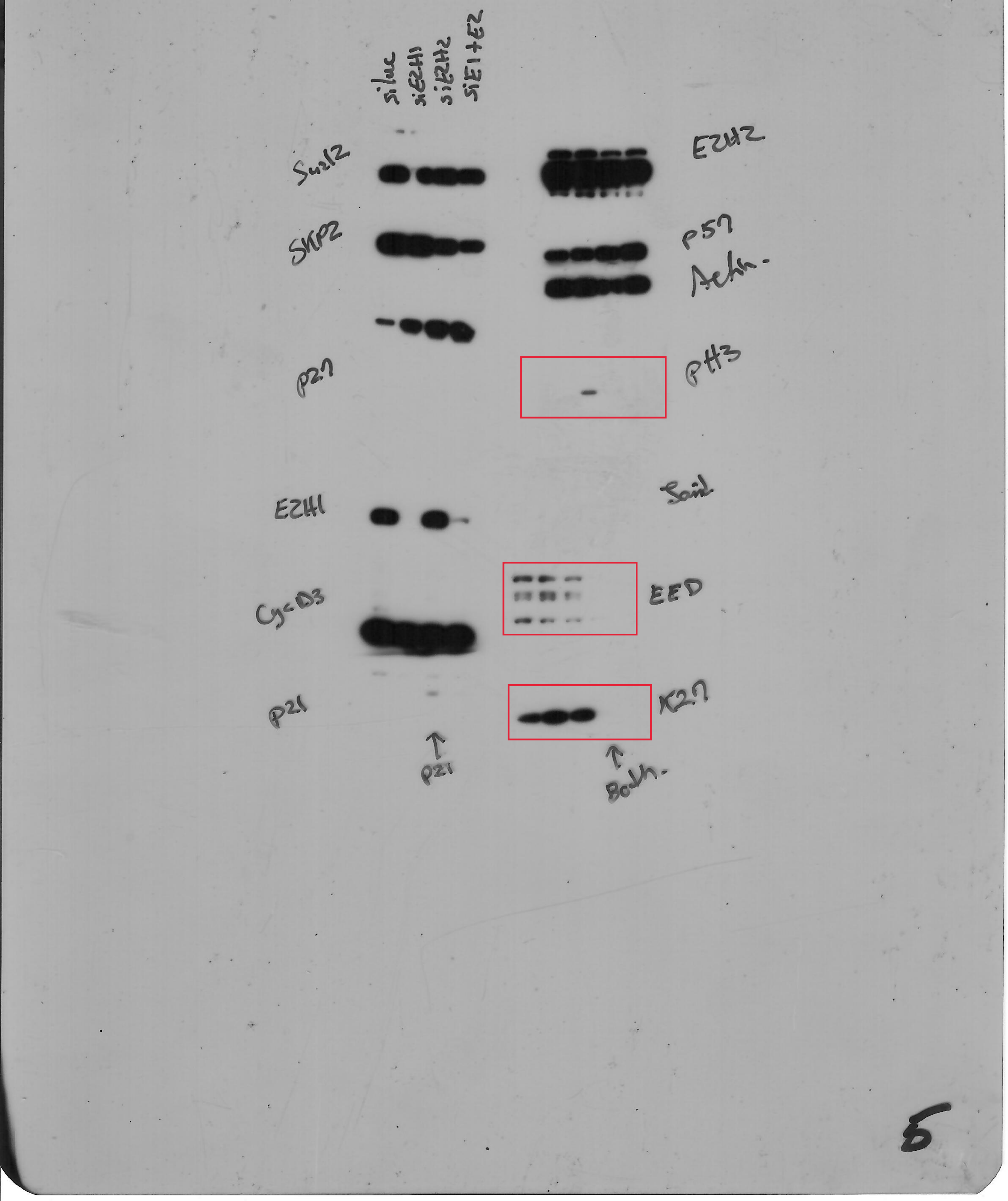

Fig. 3C. HeLa cells were transfected with Flag-tagged EZH2 or vector control (Flag c) for 24 h, followed by thymidine block or treatment with nocodazole.
Exposure #1: proteins used in the manuscript from this exposure:
Flag c, Actin, SUZ12, SKP2 (Input).

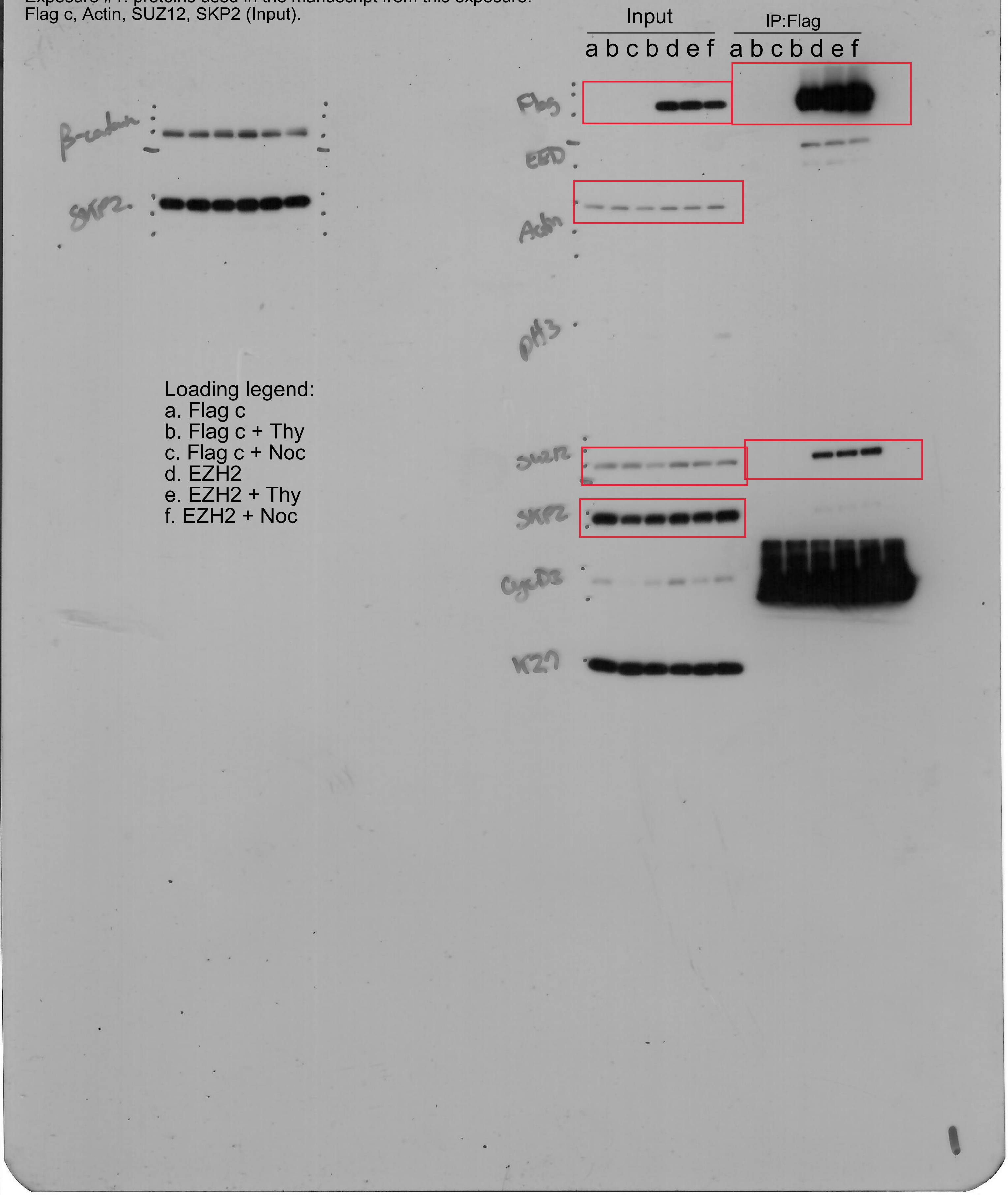

Fig. 3C. HeLa cells were transfected with Flag-tagged EZH2 or vector control (Flag c) for 24 h, followed by thymidine block or treatment with nocodazole.
Exposure #2: proteins used in the manuscript from this exposure: Cyclin D3.

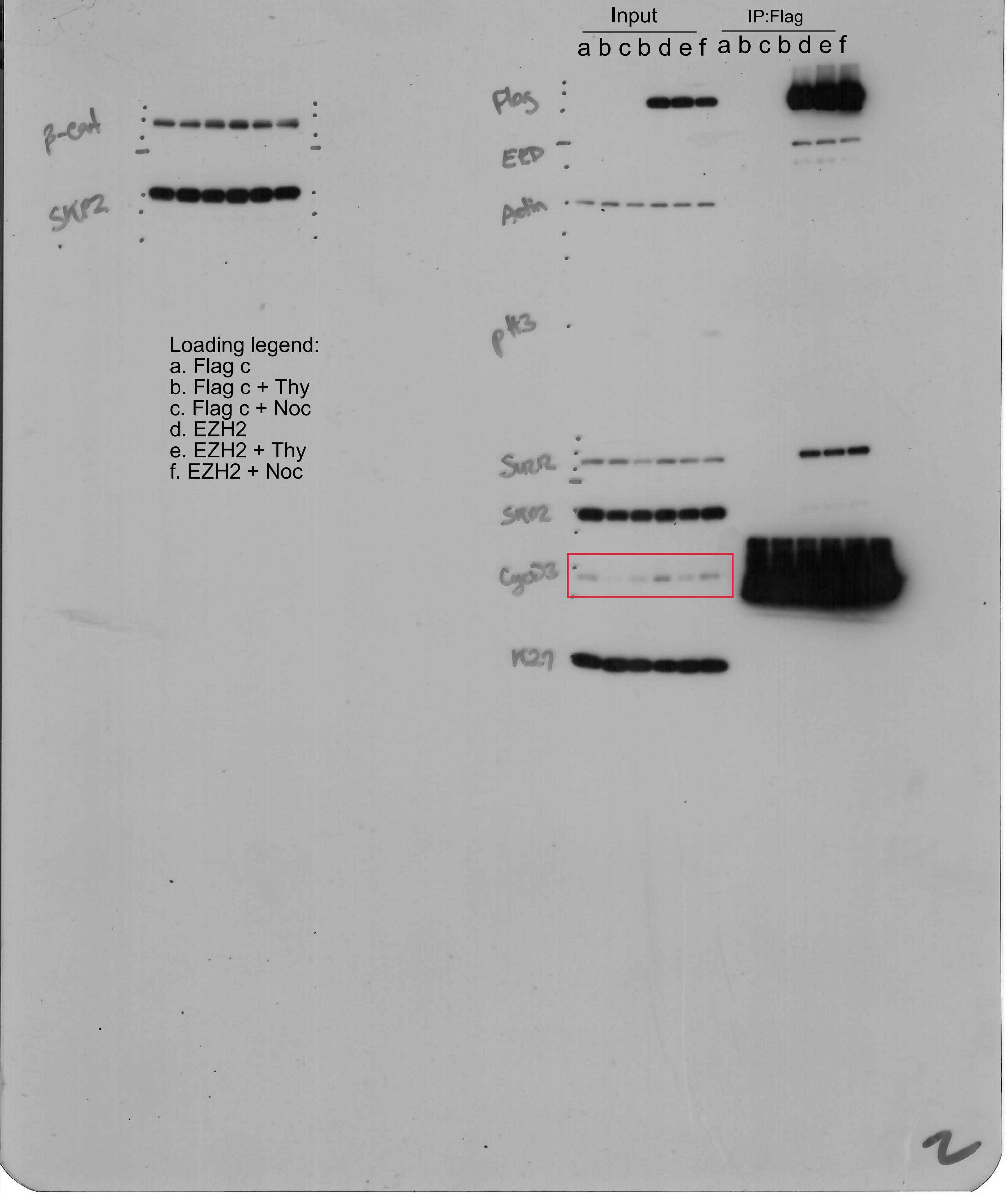

Loading legend:
a. Flag c
b. Flag c + Thy
c. Flag c + Noc
d. EZH2
e. EZH2 + Thy
f. EZH2 + Noc

Fig. 3C. HeLa cells were transfected with Flag-tagged EZH2 or vector control (Flag c) for 24 h, followed by thymidine block or treatment with nocodazole.
Exposure #4: proteins used in the manuscript from this exposure:
p-H3, SKP2 (IP).

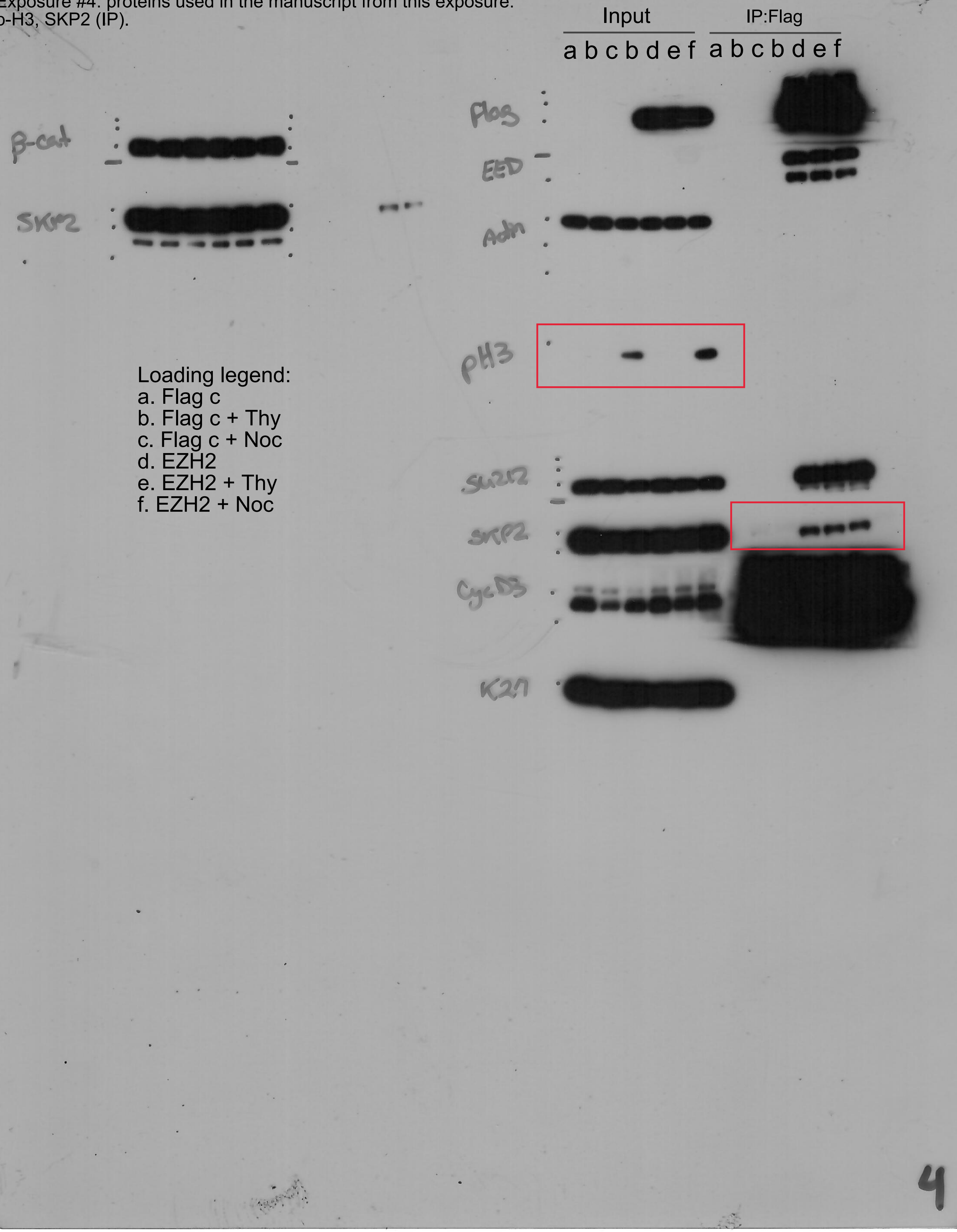

Fig. 3C. HeLa cells were transfected with FLAG-tagged EZH2 or vector control (Flag c) for 24 h, followed by thymidine block or treatment with nocodazole.
Exposure #5: proteins used in the manuscript from this exposure: none.

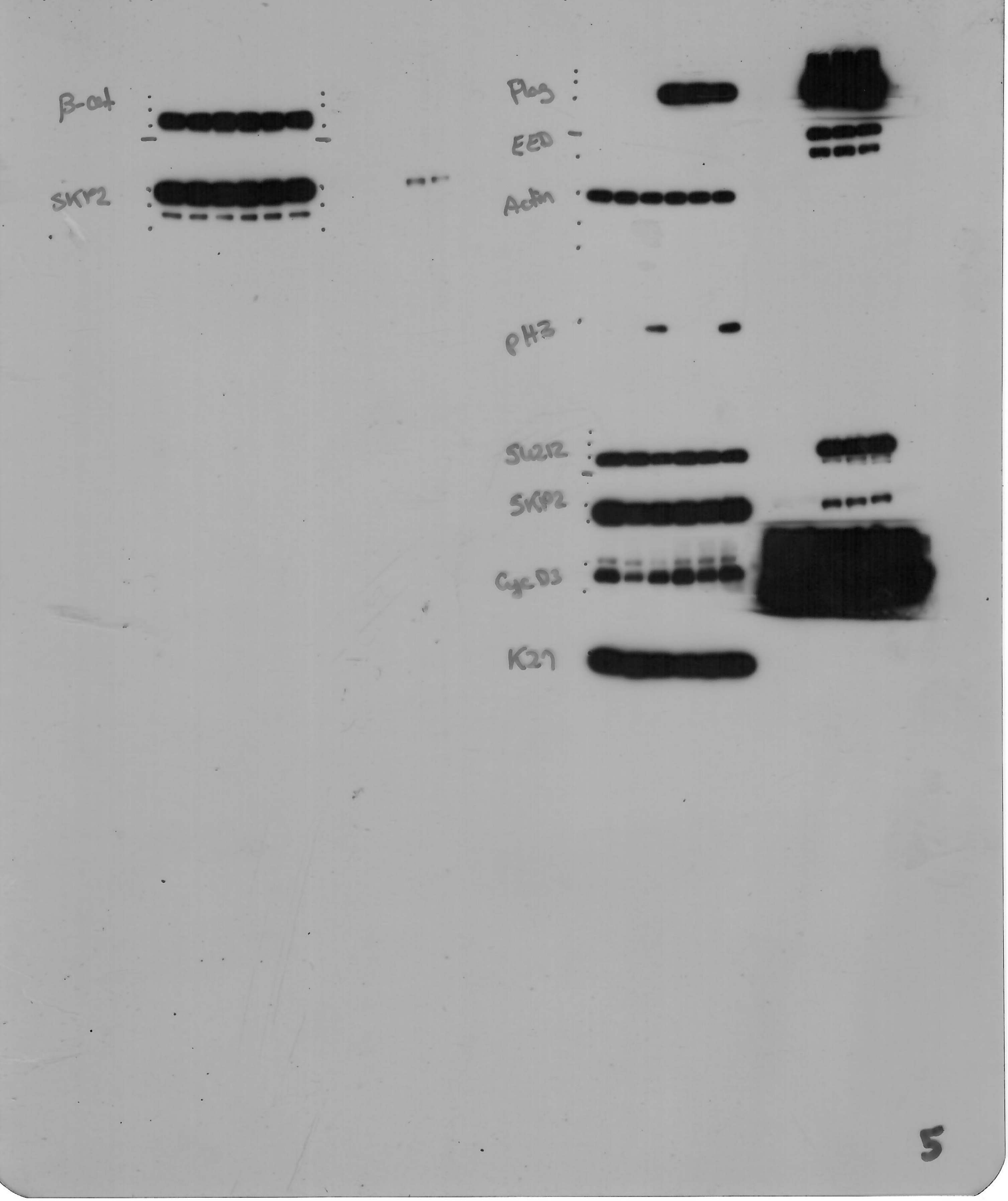

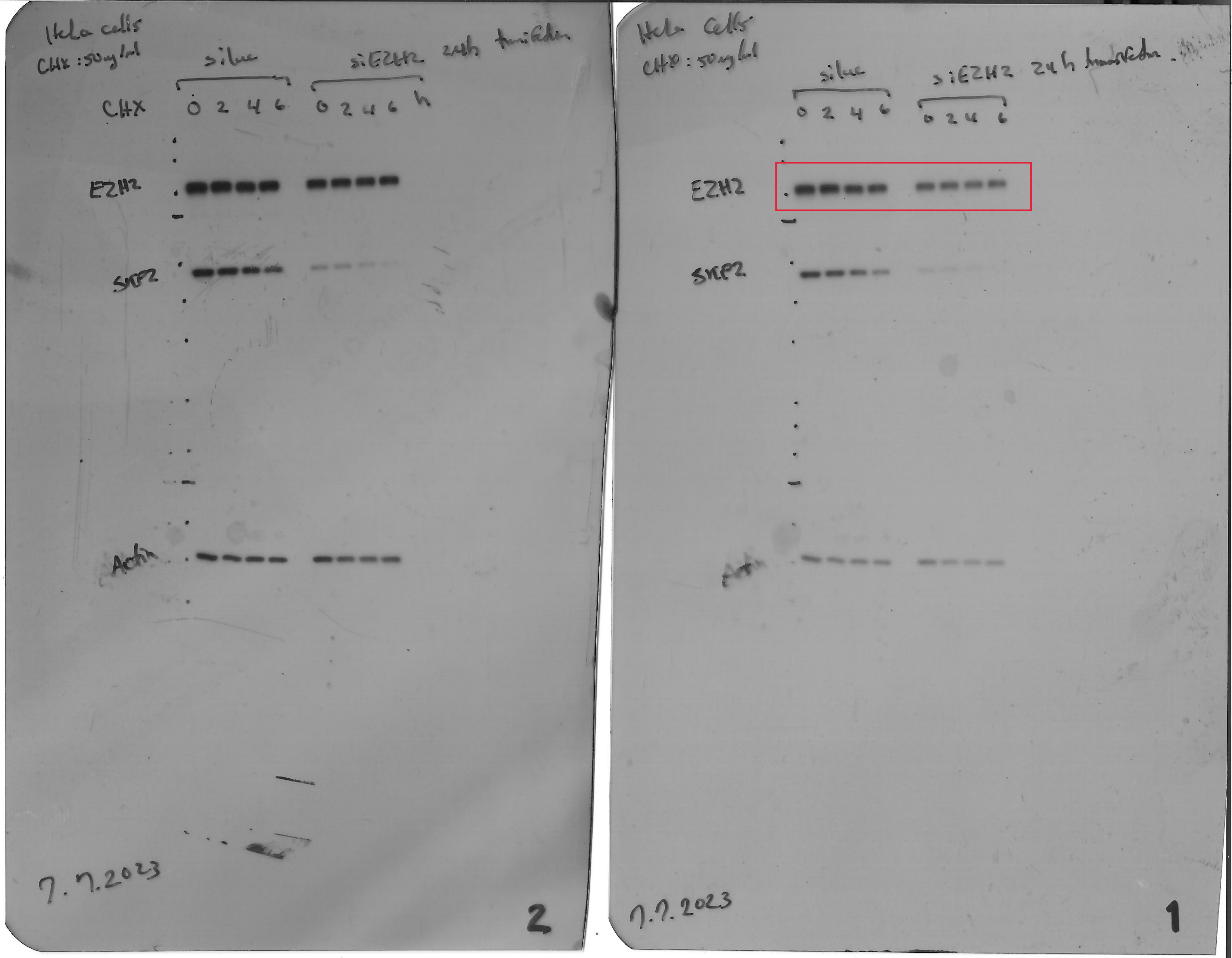

Fig. 3D. HeLa cells transfected with siEZH2 or siLUC for 24 h, then treated with 50 μg/ml of cycloheximide (CHX).
Exposures #1 and #2: proteins used in the manuscript from these exposure: EZH2.

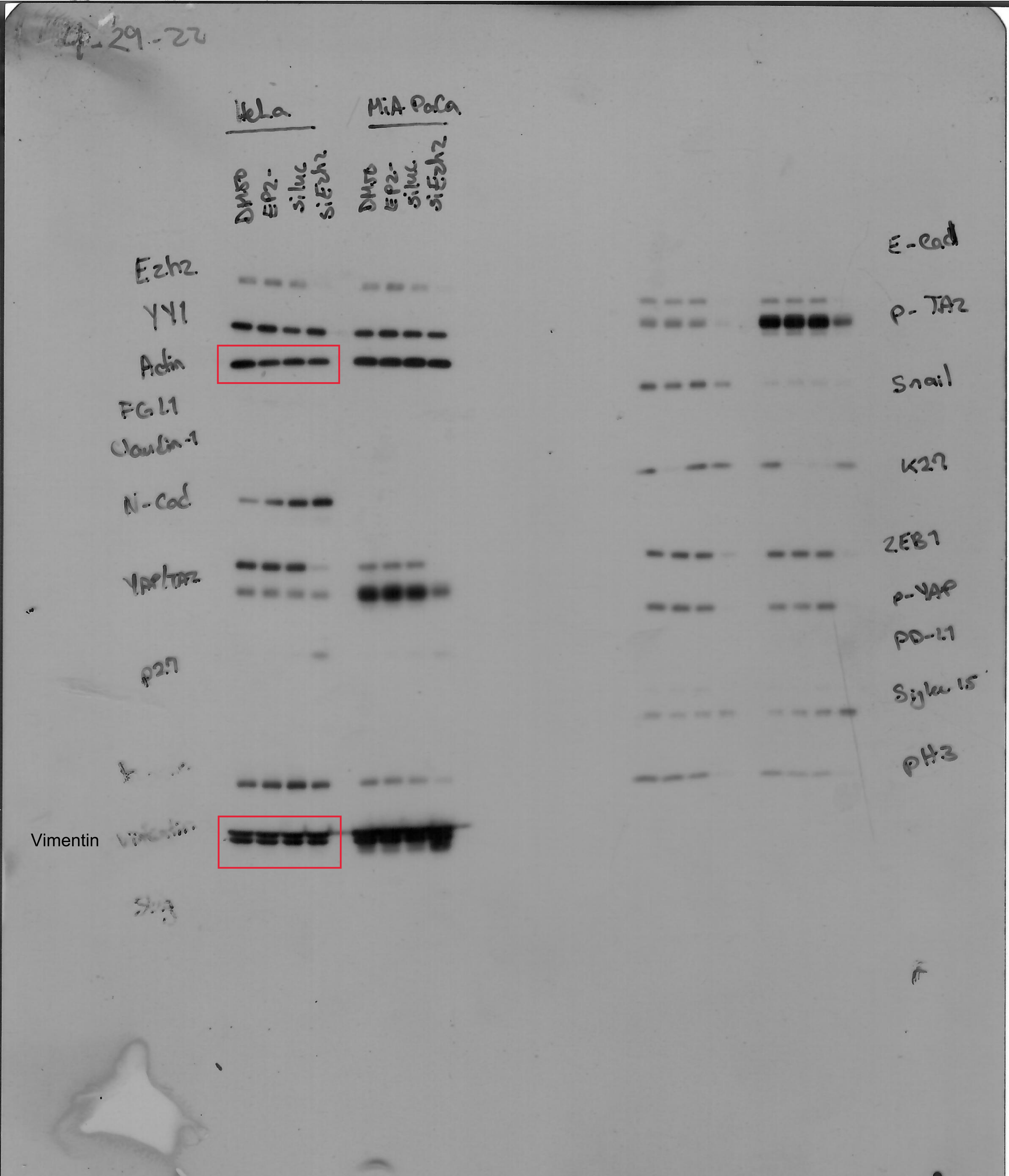

Fig. 4F. HeLa cells were treated EPZ-6438 or DMSO, transfected or trasnfected with siEZH2 or siLUC for 48h. Cell lysates were then blotted with various EMT markers.
Exposure #1: proteins used in the manuscript from this exposure: Vimentin, Actin.

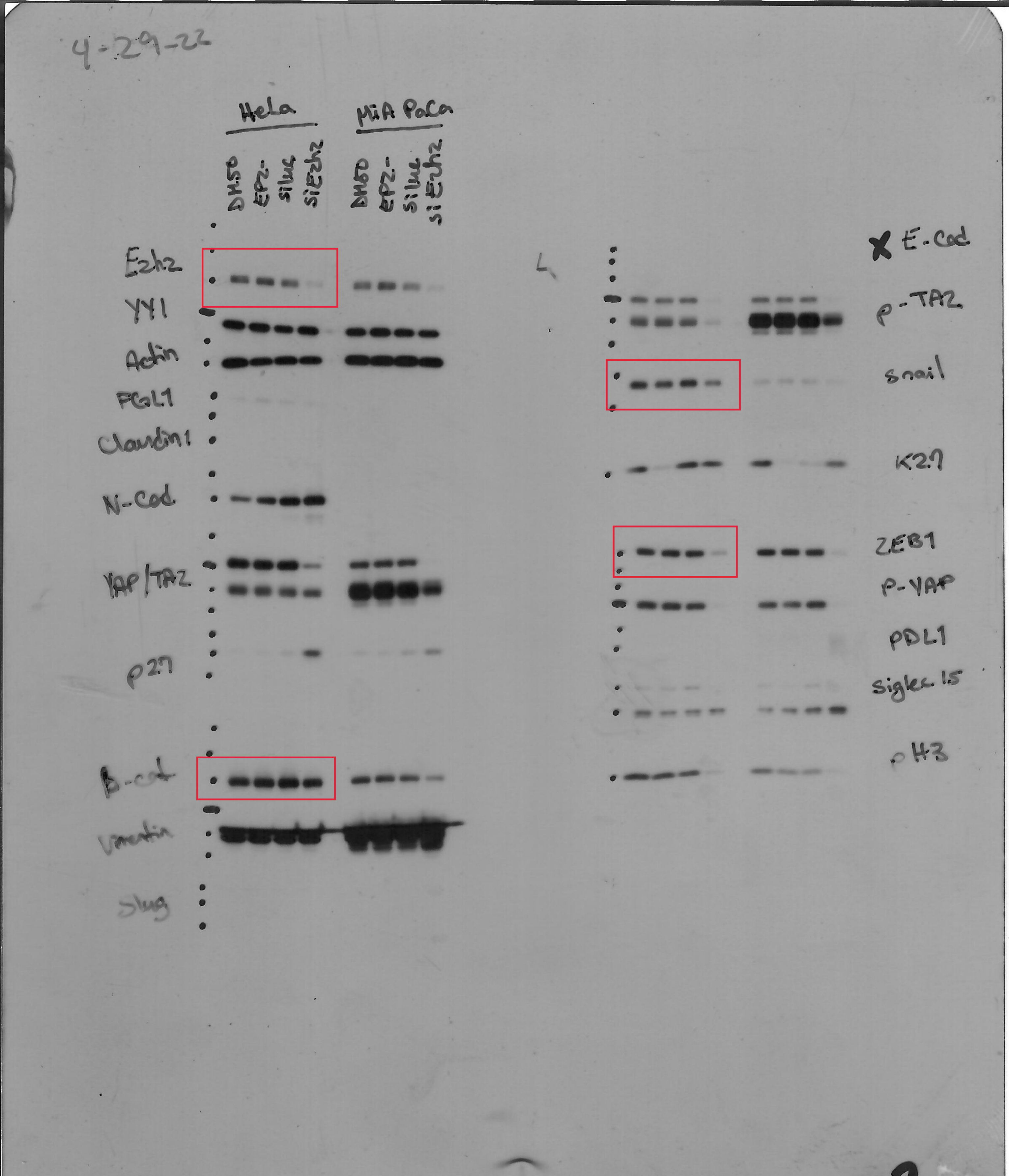

Fig. 4F. HeLa cells were treated EPZ-6438 or DMSO, transfected or trasnfected with siEZH2 or siLUC for 48h.
Cell lysates were then blotted with various EMT markers.
Exposure #2: proteins used in the manuscript from this exposure: EZH2, ZEB1, Snail, b-catenin.

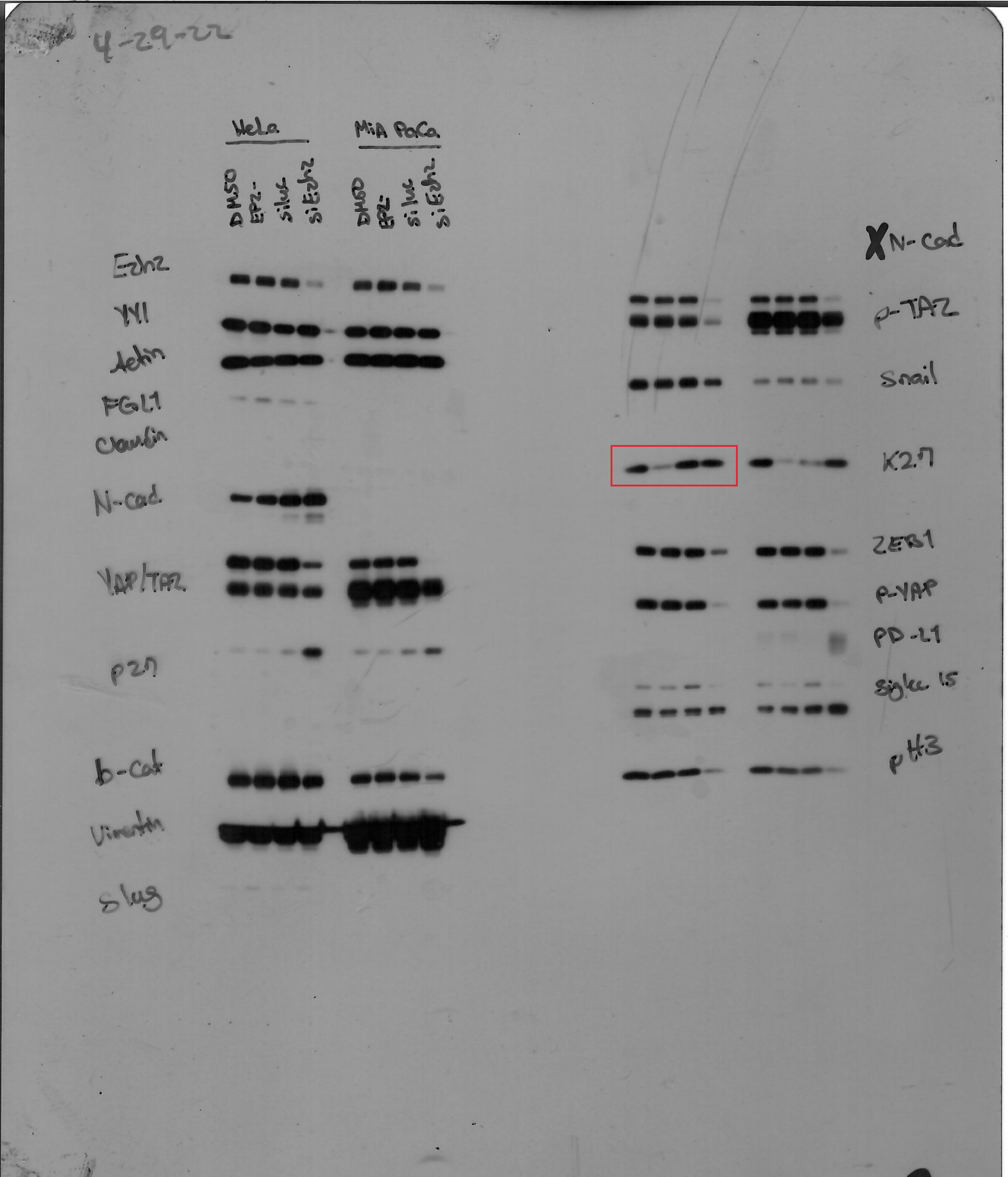

Fig. 4F. HeLa cells were treated EPZ-6438 or DMSO, transfected or trasnfected with siEZH2 or siLUC for 48h.
Cell lysates were then blotted with various EMT markers.
Exposure #3: proteins used in the manuscript from this exposure: H3K27me3.

4-29-22

Fig. 4F. HeLa cells were treated EPZ-6438 or DMSO, transfected or trasnfected with siEZH2 or siLUC for 48h.
Cell lysates were then blotted with various EMT markers.
Longest exposure: proteins used in the manuscript from this exposure: Claudin-1, SLUG.

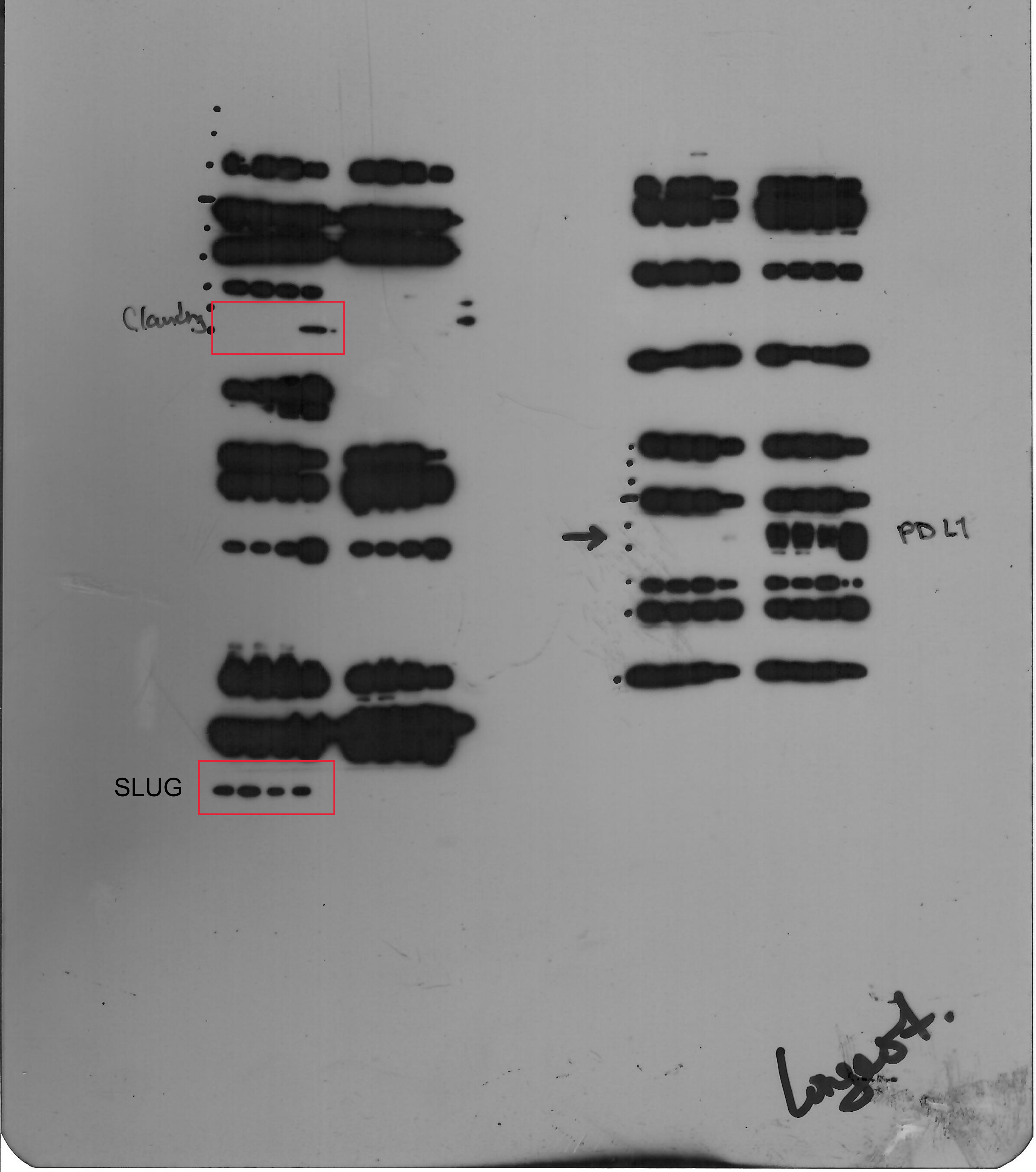

Fig. S3. Ectopic expression of EZH2 is associated with increased SKP2.
HeLa cells transfected with EZH2 WT or Flag c for 24 h.
Proteins used in the manuscript from this exposure: EZH2, SKP2, Actin.

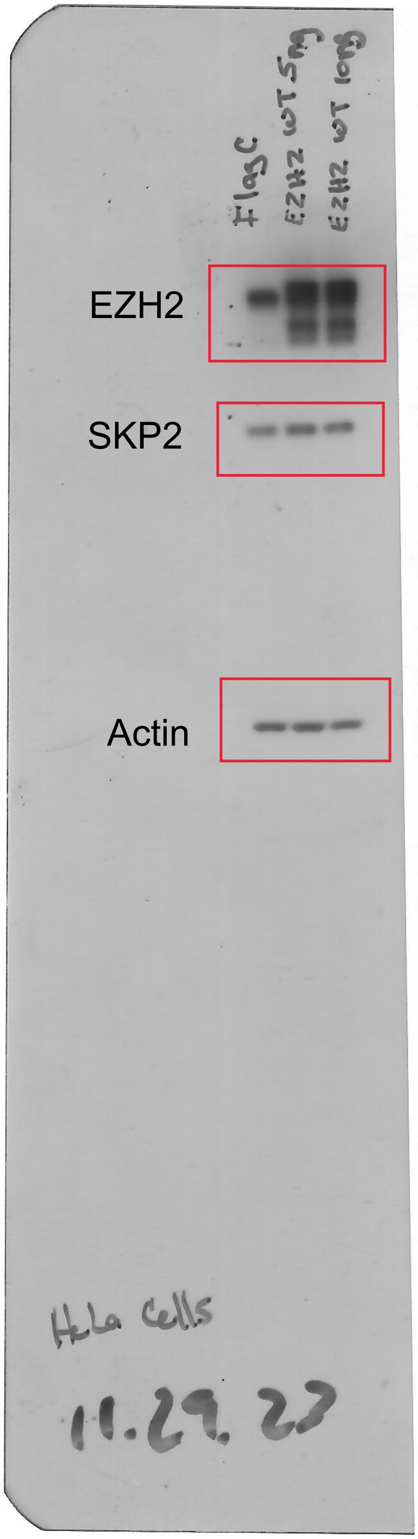

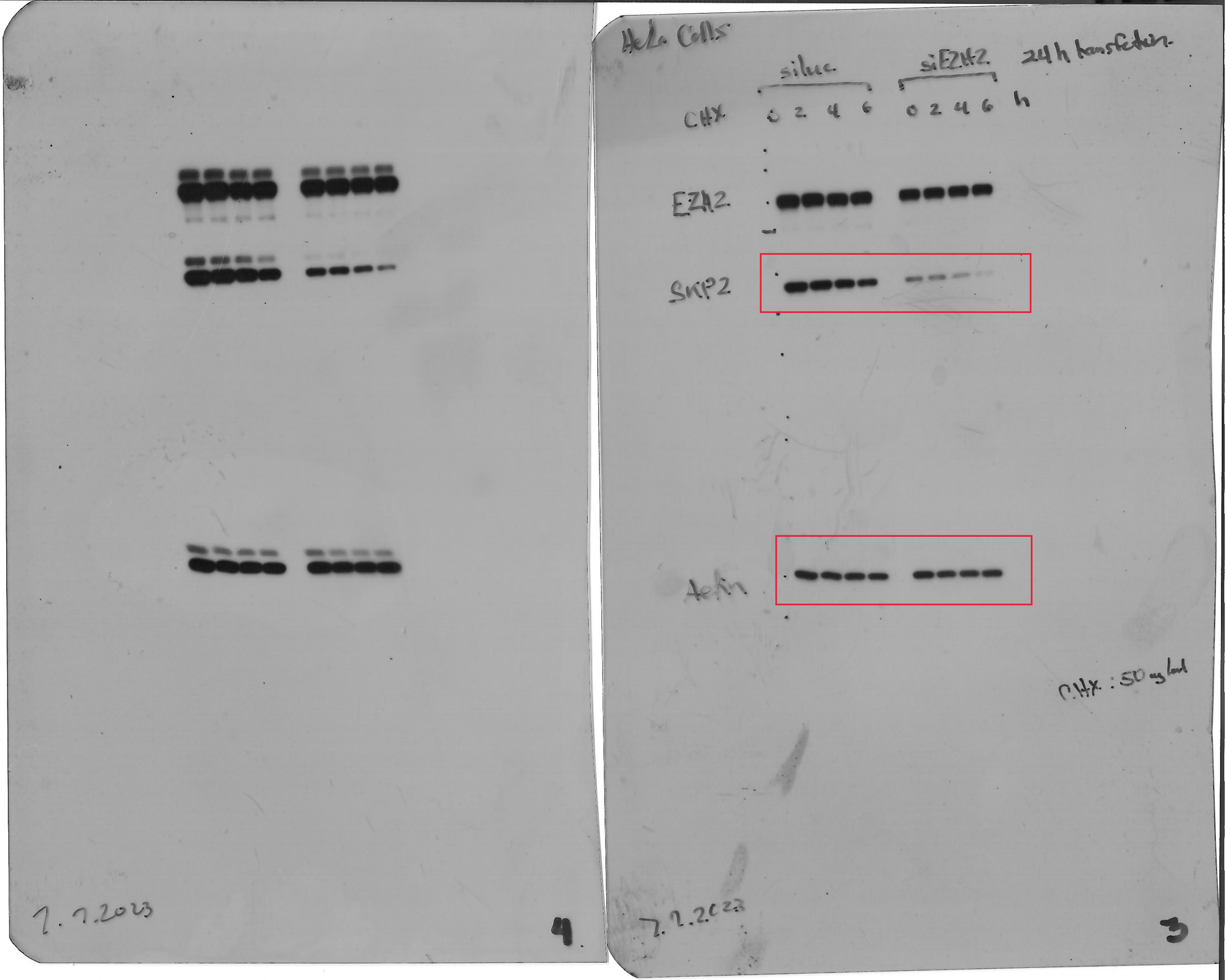

Fig. 3D. HeLa cells transfected with siEZH2 or siLUC for 24 h, then treated with 50 µg/ml of cycloheximide (CHX). Exposures #3 and #4: proteins used in the manuscript from these exposure SKP2 and Actin.
